# Conformational preference in difluoroacetamide oligomers: probing the potential for foldamers with C–H⋯O hydrogen bonds†

**DOI:** 10.1039/d3ob00811h

**Published:** 2023-06-27

**Authors:** Matej Žabka, Jonathan Clayden

**Affiliations:** a School of Chemistry, University of Bristol Cantock's Close Bristol BS8 1TS UK j.clayden@bristol.ac.uk

## Abstract

The C–H bond of a difluoroacetamide group, acidified by two adjacent fluorine atoms, could in principle provide conformational organisation for foldamers based on C–H⋯O hydrogen bonds. We find that in model oligomeric systems, this weak hydrogen bond leads only to partial organisation of the secondary structure, with the conformational preference of the difluoroacetamide groups being predominantly governed by dipole stabilisation.

## Abstract 2

The conformation of bisdifluoroacetamides is governed by dipole effects, rather than CH⋯O hydrogen bonds.

## Graphical abstract

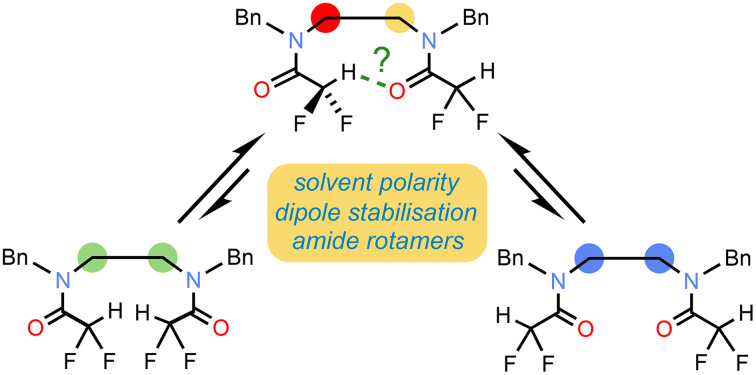

Hydrogen-bonded foldamers,^1,2^ and more recently polarity-coherent but conformationally flexible oligoureas based on ethylenediamine linkers 1 (Fig. 1A),^3^ have found widespread use in structural chemistry^4^ and catalysis,^5,6^ and as switchable devices,^7^ and have demonstrated potential biological applications.^8^ Hydrogen-bonded foldamer structures incorporate a range of structural motifs, but the most common are oligoamides and oligoureas or thioureas.^9^ These functional groups provide conformational control through strong N–H⋯O=C hydrogen bonds, similar to those governing secondary structures in peptides.^10^

**Fig. 1:**
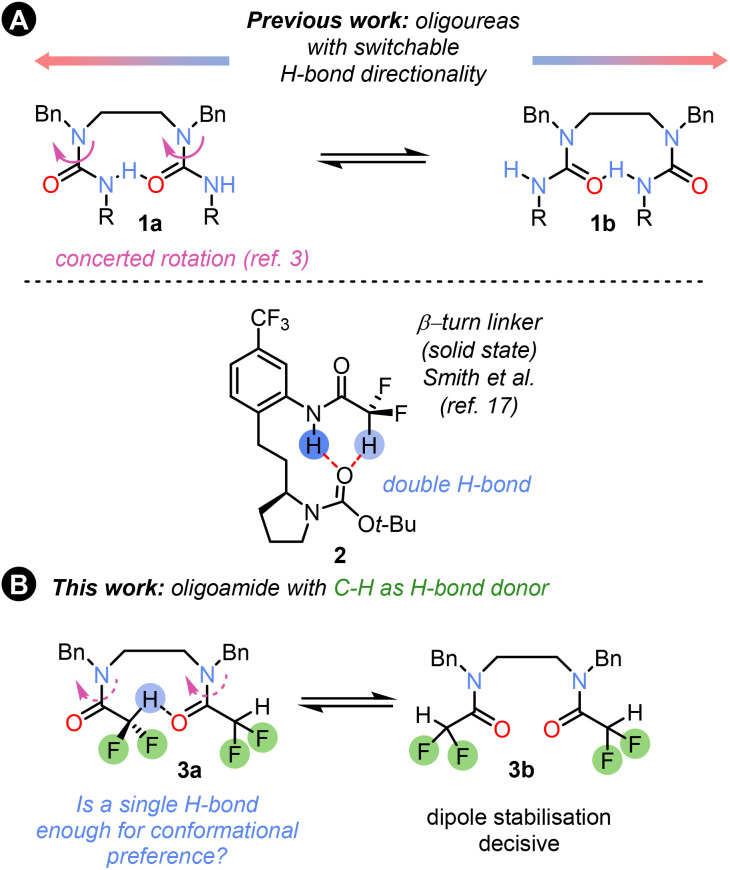
(A) Flexible oligoureas 1a can switch hydrogen bond directionality *via* concerted rotation of the individual ureas. A solid-state structure of β-turn linker with a C–H hydrogen bond donor. (B) Using a single C–H hydrogen bond for conformational control in oligomers 3 containing difluoroacetamide units.

The corresponding use of C–H⋯O=C hydrogen bonds remains almost unexplored. A simple C–H bond which can act as a hydrogen bond donor is found in the difluoromethyl group,^11,12^ which has been proposed as a bioisostere for OH and other functional groups.^13^ The CF_2_H group has recently become a target for synthetic strategies due to the valuable pharmacological properties of CF_2_H analogues of both known and new drug molecules.^14^ The different fluorination patterns and modulation of the CH–π stacking and hydrogen-bonding properties of CF_2_H groups have been used for sugar recognition^15^ and for peptide bond geometry tuning in peptides,^16^ and dual NH/CH hydrogen bonding has been used to control the solid-state conformation of β-turn linker mimics 2 (Fig. 1A).^17^

We set out to explore the conformational properties in solution of a difluoroacetamide group embedded within flexible oligomers, and its potential as a H-bond donor and conformation controller in foldamer chemistry, as well as factors influencing the conformation (Fig. 1B). In this communication we describe our findings.

First, we investigated the properties of amide 4, the mono-difluoroacetamide derivative of *N*,*N*′-dibenzylethylenediamine. The NMR of this compound revealed 1 : 1 ratio of two conformers 4a and 4b in slow exchange at room temperature in CDCl_3_. The two isomers could be easily distinguished by ^1^H, ^19^F-HOESY NMR. However, the appearance of the ^1^H and ^19^F NMR spectra was substantially affected by the amount of water present in the CDCl_3_. In anhydrous CDCl_3_, multiple peaks were present in ^19^F{^1^H} spectrum (see ESI†). By contrast, in the presence of traces of water, two sharp singlets were observed in the ^19^F{^1^H} spectrum, corresponding to the two conformers. The rate of interconversion between the conformers was quantified by 1D EXSY NMR. The rate in the presence of water was higher than in anhydrous deuterated chloroform or methanol, a solvent which is known to increase the rotational barrier.^3^ A possible explanation for this behaviour is that reversible nucleophilic addition of water molecule to the electrophilic amide C=O bond catalyses rotation around the C–N bond through a change in carbon hybridization. Amide migration from one nitrogen to the other was rejected as an explanation due to the absence of the corresponding EXSY cross-peaks. We determined the rotational barrier of the difluoroacetamide unit to be ∼73 kJ mol^−1^; for comparison, related (oligo)ureas have C–N rotational barriers in the range 49–59 kJ mol^−1^ as determined by VT-NMR.^3^

We computed the rotational barrier of a simple difluoroacetamide 5 at the DLPNO-CCSD(T1)/def2-QZVPP//B3LYP-D3(BJ)/def2-TZVP+SMD(chloroform) level of theory^18^ in ORCA computational software.^19^ The computed value matched exactly the experimental exchange rate in anhydrous chloroform. Computational exploration of the conformational preference of the difluoromethyl group within the amide showed that in the gas phase the conformer 5a with C–H bond *syn*-periplanar to the C=O bond is strongly favoured (10 kJ mol^−1^ lower in energy: Fig. 2B) over the conformer 5b with the C–F bond *syn*-periplanar to the C=O bond. However, the preference is reversed upon the inclusion of a solvent (SMD model). This is mainly due to the stabilisation of the conformer with the greater dipole moment by the solvent polarity, or rather polarizability. For an intramolecular hydrogen bond to develop between the amides, conformation 5a must be disfavoured, which would be the case in a sufficiently polar solvent.

**Fig. 2:**
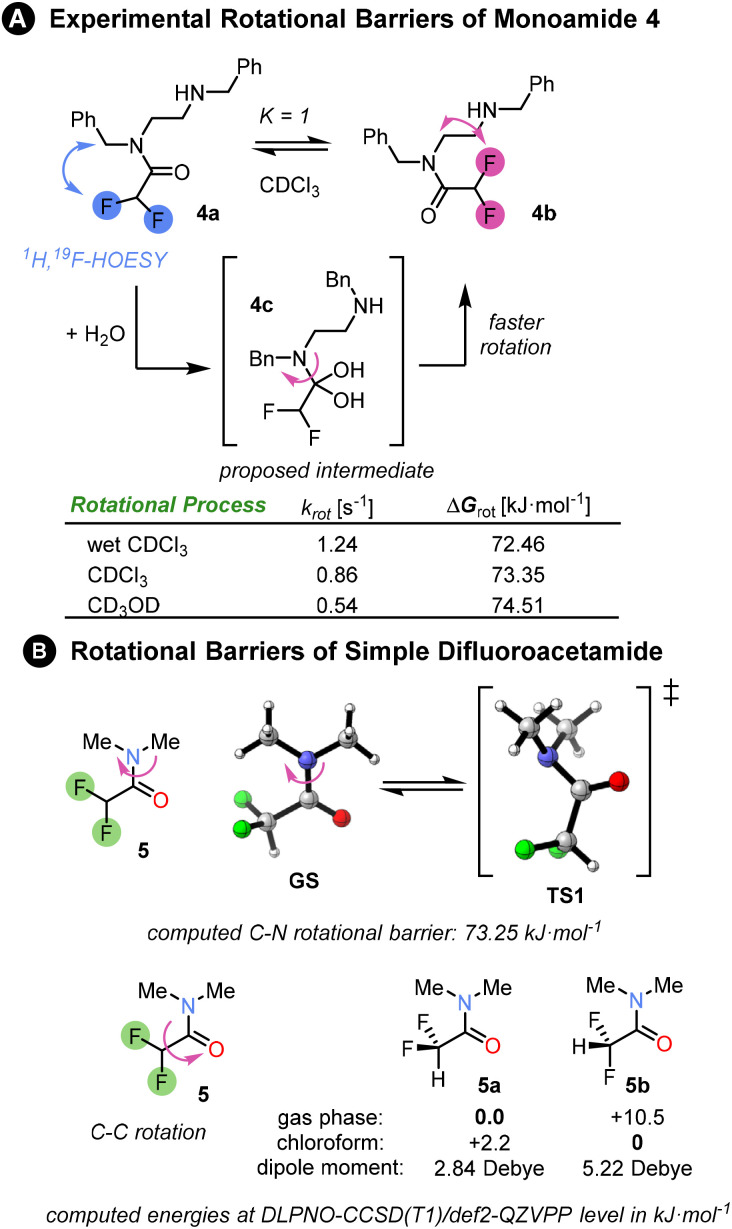
(A) Experimental rotational barriers between two monoamide isomers 4a and 4b were determined by a series of 1D EXSY experiments in different solvents at 298 K (see ESI Chapter 7†). The two isomers were populated in a 1 : 1 ratio and their structures were elucidated *via* HOESY spectrum. The fastest rotation was measured in wet CDCl_3_, whereas the slowest rotation was in deuterated methanol. The experimental rate constants and free energies of rotation are summarised in the table. (b) Rotational barriers in a simple difluoroacetamide 5 as well as conformational preference of difluoromethyl group matches the experimental barrier in chloroform. The Gibbs free energies Δ*G*_298_ were computed at DLPNO-CCSD(T1)/def2-QZVPP level of theory based on B3LYP-D3(BJ)/def2-TZVP structures and include SMD solvation correction in chloroform.

To probe interactions between the difluoroacetyl groups, we synthesized bis(amide) 3 from a diamine using excess difluoroacetic anhydride and pyridine in DCM. The ^1^H and ^19^F NMR spectra at 298 K indicated three principal conformational states – one unsymmetrical (*E*,*Z*-3a with the ethylene linkage appearing as ^1^H multiplets) and two symmetrical (*Z*,*Z*-3b and *E*,*E*-3c with the ethylene linkage appearing as a singlet) in a ratio 3a : 3b : 3c 49 : 41 : 10. These are the three possible combinations of the amide rotamers. Again, the NOESY and HOESY analysis in CDCl_3_ enabled assignment of the individual conformers. The ratio remained the same in benzene-*d*_6_ or in an 80% CS_2_ in CDCl_3_ solvent mixture with almost identical polarizabilities, but population of the unsymmetrical conformer increased when acetone-*d*_6_ was used (for more solvents see ESI Chapter 6†). The origin of this preference could lie either in the formation of a hydrogen bond or in cooperative alignment of the amides, or in a change in the dipole stabilising capacity of the solvent. The effect of solvent polarity on conformation has also been noted in trifluorocyclohexanes.^20^ The exchange between the conformers was detected at 298 K in a 2D EXSY spectrum. The ^19^F, ^19^F-EXSY spectrum revealed the sequential rotation of each individual amide, but only extremely slow concerted rotation of both amide groups (see Fig. 3A). All these pieces of evidence point towards a minor role for any possible weak hydrogen bond formed from the C–H hydrogen-bond donor.

**Fig. 3:**
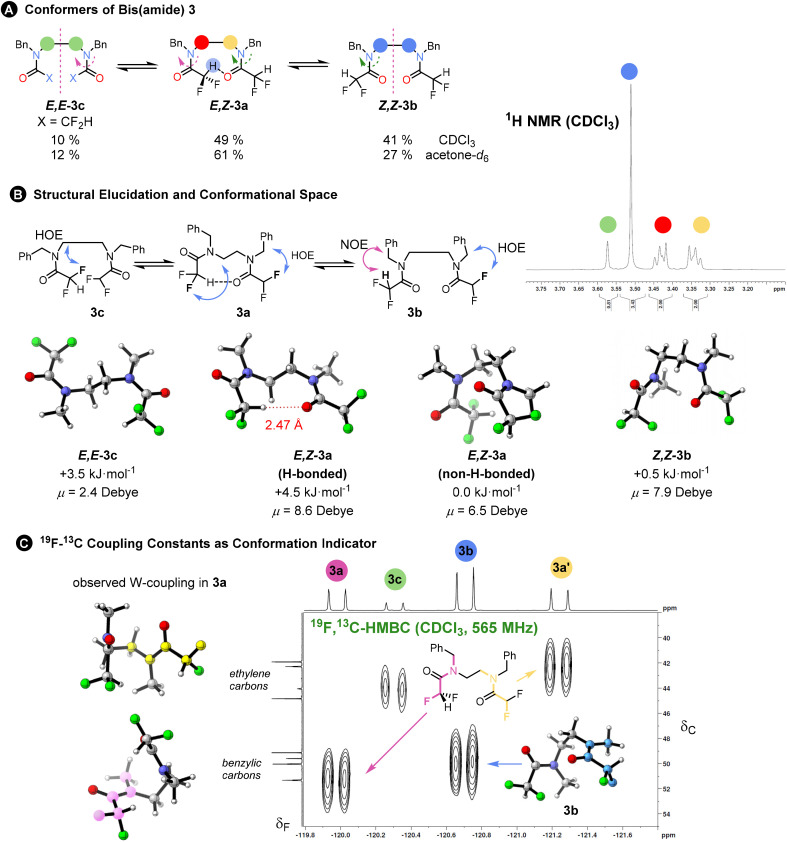
(A) Single rotations are responsible for interconversion between isomers 3a–c as determined by a 2D ^19^F, ^19^F-EXSY experiments. The isomer ratio changed in more polar acetone with higher preference for the unsymmetric isomer *E*,*Z*-3a. The ^1^H NMR (400 MHz, CDCl_3_) inset shows the signal of the ethylene linkage for the different isomers: symmetric isomers exhibit singlet, while unsymmetric conformer two multiplets. (B) ^1^H, ^19^F-HOESY NMR was instrumental in structural elucidation of the conformers. The calculated 3D structures of the most populated conformers are shown. The structures were simplified for the sake of the computations; *N*,*N*′-dimethyl derivatives were used instead of *N*,*N*′-dibenzyl derivatives. They have been shown to reproduce the actual populations accurately.^3^ The computed Gibbs free solvation energies as well as dipole moments in the gas phase are given. (C) Further indication of conformational preference and not conformational averaging of the difluoromethyl group rotation: unusually large ^4^*J*_CF_ coupling constants suggest strong W-type coupling. The connectivity was confirmed by a ^19^F, ^13^C-HMBC spectrum and two W-couplings are shown for 3a (right): fluorine to benzylic carbon (magenta) and fluorine to ethylene carbon (yellow). A large coupling was observed for 3b (blue; inset). The individual coupling constants were also computed using DFT.

Within each of these three principal states, further conformations exist that interconvert too rapidly to allow detection by NMR. A computational search found at least 17 conformations within a 7 kJ mol^−1^ threshold mostly due to difluoromethyl group rotations. The overall computed ratio of 3a : 3b : 3c is 37 : 33 : 30 and the major populated conformers are shown in Fig. 3B.

The freely rotating CF_2_H group appears as a triplet in the ^1^H and a doublet in the ^19^F NMR spectra, with ^2^*J*_HF_ ∼ 54 Hz, which remains invariant between different conformers. However, certain benzylic or aliphatic ^13^C{^1^H} resonances appeared as triplets, due to an unusually large ^4^*J*_CF_ of around 4 Hz.

These correlations were confirmed by a ^19^F, ^13^C-HMBC spectrum and suggest W-coupling across the coplanar F–C–C(O)–N–C motif evident in some of the computed conformers (see Fig. 3C).

We reproduced the magnitude of these ^4^*J*_CF_ coupling constants by computing them at the DSD-PBEP86/pcSseg-3 level of theory. The computed couplings are around 2 Hz, which might exclude conformational averaging and rather a conformational preference. The experimental value is larger than any computed coupling(s), which are slightly overestimated. For the main conformation of 3b, we found the largest computed constant (4 Hz) for the atom arrangement in blue shown in the inset (Fig. 3C). This computational method gave also correct numbers for other observed couplings of varying magnitudes (^1^*J*_CF_, ^2^*J*_CF_, ^2^*J*_HF_, *etc*. see Table 1 and ESI Chapter 10†).

**Table 1:** Experimental (in CDCl_3_) and averaged calculated *J* couplings of 3a (non-H-bonded; conf23b) at DSD-PBEP86/pcSseg-3 level of theory

Coupling type	Experimental [Hz]	Calculated [Hz]
^1^ *J*_CF_	253	264
^2^ *J*_CF_	25	28
^4^ *J*_CF_	3.8	1.9/−1.1
^1^ *J*_CH_	192	217
^2^ *J*_HF_	54	61

Single-crystal X-ray structure of 3 showed a symmetric conformation 3b′, H-bonded to solvent CHCl_3_ molecules. Intermolecular CF_2_H–phenyl noncovalent interactions are evident in the crystal packing (see Fig. 4 and ESI Chapter 9†). However, in solution this structure accounts for only ∼5% population.

**Fig. 4:**
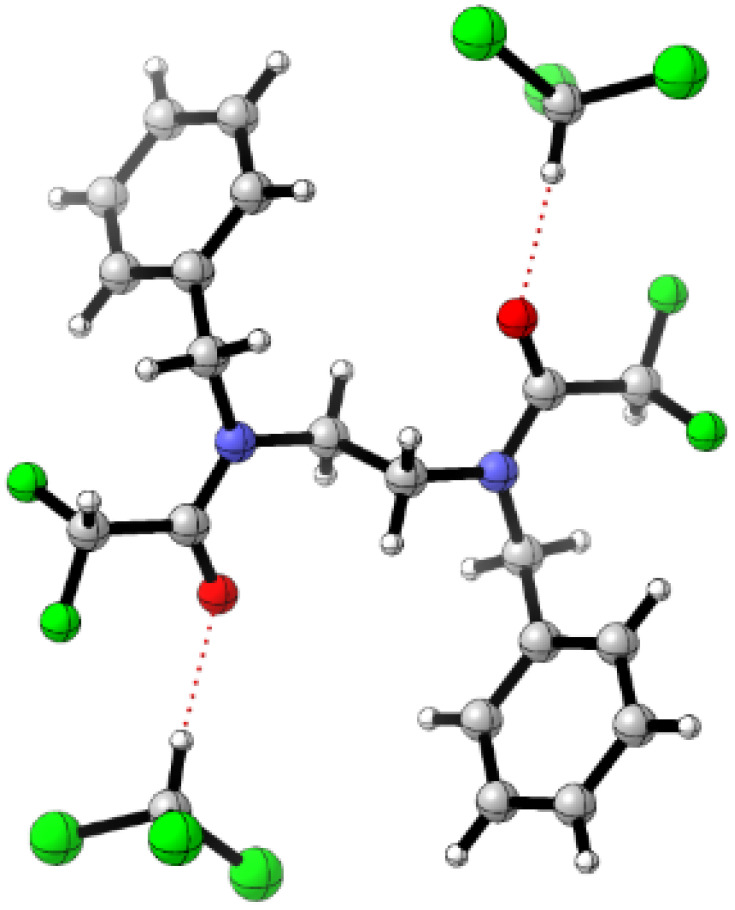
Single crystal X-ray structure of 3, adopting conformation 3b′·(CHCl_3_)_2_ stabilised by intermolecular CF_2_H–phenyl interactions (see ESI Chapter 9†). The computed dipole moment is zero.

Because ureas are excellent H-bond donors and acceptors, we synthesised compound 6 to test its ability to form either an NH or CH H-bond with a single amide unit. In CDCl_3_, a major conformation 6a with urea acting as the H-bond donor was identified (Fig. 5). A weak NH exchange peak indicated the presence of urea rotamer 6b, where the urea is no the longer H-bond donor. In contrast, in acetone-*d*_6_, the increased solvent polarity affected the populations.

**Fig. 5:**
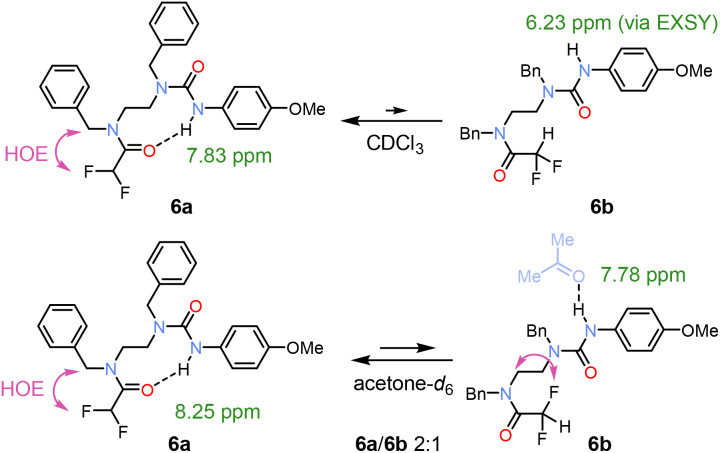
Conformers of urea 6 in CDCl_3_ and acetone-*d*_6_. In CDCl_3_, the major H-bonded urea 6a shows downfield shift of an NH proton, whereas the minor urea conformation 6b was observed in EXSY spectrum after NH irradiation. The higher population of 6b in acetone was the result of increased polarity and H-bond accepting ability of the solvent.^4^

The two conformations were observed in 2 : 1 ratio, which allowed us to identify the isomer 6b by ^1^H, ^19^F-HOESY interactions.

Taken together, these data show that there exists a conformational preference in difluoroacetamide oligomers that can be influenced by the solvent, but which is not solely due to the H-bond as the controlling element.

## Conclusions

Overall, we found that a unidirectional chain of hydrogen bonds is not readily attained in an oligomer composed of difluoroacetamide units acting as C–H hydrogen bond donors, due to population of unfavourable conformations of the difluoromethyl group. However, some preference is clearly visible, and the conformational space can be partially controlled using more polar solvents, which disfavour the conformation where the C–H bond is coplanar with the amide C=O bond. NMR exchange experiments shed light on the rotational processes and showed that a single rotation is much faster than the geared rotation of two amide units. Unusually large ^4^*J*_CF_ coupling constants were detected at room temperature, suggesting slight conformational preference as supported by the DFT calculations. We believe these findings will help inform future design of molecular devices that rely on fine-tuning of non-covalent interactions.

## Conflicts of interest

There are no conflicts to declare.

## Supplementary Material

OB-021-D3OB00811H-s001

OB-021-D3OB00811H-s002
