# Supplementary material for: Conformational preference in difluoroacetamide oligomers: probing the potential for foldamers with C–H⋯O hydrogen bonds
Source: Org Biomol Chem. 2023 Jun 27;21(29):5939–43. doi: 10.1039/d3ob00811h (PMC10369364; doi:10.1039/d3ob00811h)
Supplement: OB-021-D3OB00811H-s001 [file OB-021-D3OB00811H-s001.pdf]

This journal is © The Royal Society of Chemistry 2023

Conformational Preference in Difluoroacetamide Oligomers: Probing the Potential for Foldamers with C-H···O Hydrogen Bonds

Matej Žabka and Jonathan Clayden*

School of Chemistry, University of Bristol, Cantock's Close, Bristol BS8 1TS, UK

Email: j.clayden@bristol.ac.uk

Supplementary Material:

Contents

1. General Information 2
2. NMR Spectrometer Data 2
3. NMR Pulse Sequences 3
4. Experimental Procedures 4
N-Benzyl-*N*-(2-(benzylamino)ethyl)-2,2-difluoroacetamide (4) 4
N,N'-(Ethane-1,2-diyl)bis(*N*-benzyl-2,2-difluoroacetamide) (3) 5
N-benzyl-*N*-(2-(1-benzyl-3-(4-methoxyphenyl)ureido)ethyl)-2,2-difluoroacetamide (6) 6
5. Structural Identification 7
Assignment of Chemical Shifts 7
NMR Spectra 8
Compound 3 8
Compound 4 20
6. Solvent Effects 25
7. NMR Exchange Studies of Rotational Barriers 26
8. Urea Donor/Acceptor System 28
9. Crystallography 34
10. Computational Details 38
Geometry Optimization 38
Single-Point Calculations 38
Gibbs Free Energies 38
NMR Calculations 38
Computed Energies 39
Computed Dipole Moments 42
Computed NMR *J*-Coupling 42
Coordinates of Optimized Structures 46
11. References 78

1. General Information

All reactions were performed under a nitrogen atmosphere. Chemicals were purchased from Sigma Aldrich (Merck) and used without any further purification. Difluoroacetic anhydride was purchased from Manchester Organics. Deuterated solvents were purchased from Cambridge Isotope Laboratories or Sigma Aldrich (Merck). Anhydrous solvents were dispensed under nitrogen from a solvent purification system (Innovative Technologies PureSolve PS-MP-5) or were purchased from commercial suppliers.

High resolution spectrometry experiments (HRMS) were recorded on an Orbitrap Elite Thermo Scientific for electrospray ionisation experiments.

2. NMR Spectrometer Data

NMR experiments were performed on a Bruker Avance III HD 500 Cryo equipped with 5 mm DCH 13C–1H/D Cryo Probe (500 MHz). The temperature was controlled by Bruker BCU II unit. Additional ^{19}F experiments were performed on a Bruker Neo 600 MHz equipped with 5 mm TXO cryoprobe (cryo-enhanced for ^{19}F) at 298 K; and Bruker Avance 400.

Solvent residual peak (7.26 ppm for ^{1}H and 77.0 ppm for ^{13}C in $CDCl_3$; 2.05 for ^{1}H and 29.84 ppm for ^{13}C in acetone-d_6) were used as references for ^{1}H and ^{13}C chemical shifts. ^{19}F NMR spectra were referenced to internal standard hexafluorobenzene (-161.64 ppm in $CDCl_3$; -164.67 in acetone-d_6) according to Togni et al.[1]

The spectra were plotted in Mestrenova 14.2.1 or TopSpin 4.1.4 using standard processing methods.

3. NMR Pulse Sequences

Standard Bruker pulse sequences with the following parameters were used throughout the work.

^{1}H-NMR: zg30; Relaxation delay = 2 s, Acquisition time = 4.36 s, SW = 30.0 ppm, TD = 64k, NS = 8 – 32.

^{13}C{^{1}H}-NMR: Pulse program: zgpg30; Relaxation delay = 1.00 s, Acquisition time = 1.90 s, SW = 248.0 ppm, TD = 120k, NS = 512.

^{19}F{^{1}H}-NMR: Pulse program: zgig; Relaxation delay = 2.00 s, Acquisition time = 1.15 s, SW = 300.0 ppm, TD = 256k, NS = 32 – 64.

^{19}F-NMR: Pulse program: zg30; Relaxation delay = 2.00 s, Acquisition time = 1.15 s, SW = 300.0 ppm, TD = 256k, NS = 32 – 64.

^{19}F-NMR: Pulse program: zgbs; Relaxation delay = 1.00 s, Acquisition time = 0.5 s, SW = 233.0 ppm, TD = 8k, NS = 4.

1D selective NOESY: Pulse program: selnogpzs with 180° Gaussian shaped pulse; Relaxation delay = 3.0 s, Acquisition time = 3.27 s, SW = 20.0 ppm, TD = 64k, NS = 64, mixing time D8 = 100 – 500 ms.

^{1}H,^{1}H-COSY: pulse program: cosygpmfppqf; Relaxation delay = 0.92 s, Acquisition time = 0.18 s (F2), SW = 12.0 ppm (F2), 12.0 ppm (F1); TD = 4k (F2), 256 (F1), NS = 2.

^{1}H,^{13}C-HSQC: pulse program: hsqcedetgpsp.3; Relaxation delay = 0.8 s, Acquisition time = 0.07 s (F2), SW = 14.0 ppm (F2), 165 ppm (F1); TD = 1k (F2), 256 (F1), NS = 2, cnst2 = 145 Hz.

^{1}H,^{13}C-HMBC: Pulse program: hmbcetgpl3nd; Relaxation delay = 1.5 s, Acquisition time = 0.27 s, SW = 15.0 ppm (F2), 240 ppm (F1), TD = 4k (F2), 512 (F1), NS = 4, cnst13 = 8 Hz.

^{1}H,^{1}H-NOESY: Pulse program: noesygpph; Relaxation delay = 3.0 s, Acquisition time = 0.51 s, SW = 8 ppm (F2), 8 ppm (F1), TD =4k (F2), 256 (F1), NS = 4, mixing time D8 = 500 ms.

^{1}H,^{19}F-HOESY: Pulse program: hoesygpndqn.mo; Relaxation delay = 3.0 s, Acquisition time = 0.09 s, SW = 15 ppm (F2), 6 ppm (F1), TD =1k (F2), 128 (F1), NS = 8 – 12, mixing time D8 = 500 ms.

^{19}F,^{19}F-COSY: pulse program: cosygpppqf; Relaxation delay = 2.0 s, Acquisition time = 0.18 s (F2), SW = 10.0 ppm (F2), 10.0 ppm (F1); TD = 2k (F2), 128 (F1), NS = 2.

^{19}F,^{19}F-EXSY: Pulse program: noesygpphpp; Relaxation delay = 3.0 s, Acquisition time = 0.20 s, SW = 8.9 ppm (F2), 8.9 ppm (F1), TD =2k (F2), 64 (F1), NS = 4, mixing time D8 = 500 ms.

^{19}F,^{13}C-HSQC: pulse program: hsqcetgpsisp2.2; Relaxation delay = 1.0 s, Acquisition time = 0.1 s (F2), SW = 14.0 ppm (F2), 165 ppm (F1); TD = 1500 (F2), 128 (F1), NS = 2, cnst2 = 10 – 250 Hz.

^{19}F,^{13}C-HMBC: Pulse program: hmbcgplndqf; Relaxation delay = 1.0 s, Acquisition time = 0.12 s, SW = 14.0 ppm (F2), 220 ppm (F1), TD = 1800 (F2), 128 (F1), NS = 4 – 8, cnst13 = 2.5 – 10 Hz.

4. Experimental Procedures

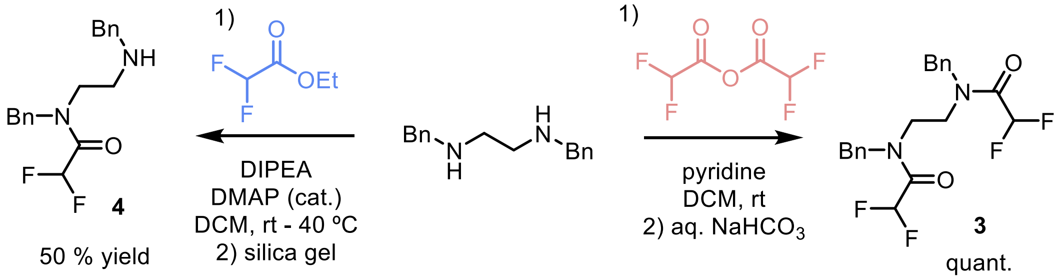

***N*-Benzyl-*N*-(2-(benzylamino)ethyl)-2,2-difluoroacetamide (4)**

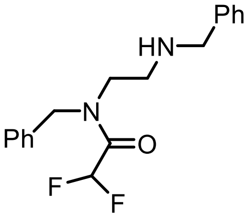

Procedure A: N,N'-Dibenzylethylenediamine (120 mg, 0.5 mmol) and ethyl difluoroacetate (230 µL, 272 mg, 2.2 mmol, 4.4 eq.) were mixed and stirred at rt for 15 min. The flask was then heated to 50 °C in a DrySyn heating block for 2 h. Additional ethyl difluoroacetate (250 µL) was added, the mixture was stirred at 50 °C for 1 h and then stirred at rt overnight. The ester was then evaporated, and the crude mixture was purified by silica gel column chromatography (DCM/MeOH 99:1 – 95:5) to give the product (76 mg, 48 % yield) as a pale-yellow viscous oil.

Procedure B: N,N'-Dibenzylethylenediamine (120 mg, 0.5 mmol) was dissolved in DCM (2 mL). DMAP (10 mg), ethyl difluoroacetate (300 µL, 2.85 mmol, 5.7 eq.), and DIPEA (200 µL, 1.15 mmol, 2.3 eq.) were added. The mixture was stirred and heated to 45 °C in a DrySyn heating block for 2 h, followed by stirring at rt overnight. The solvent was then evaporated, and the crude mixture was purified by silica gel column chromatography (DCM/MeOH 99:1 – 97:3) to give the product (80 mg, 50 % yield) as a pale-yellow viscous oil.

^{1}H NMR (500 MHz, dry $CDCl_3$ as ~1.2:1 mixture of rotamers; major rotamer denoted by *): δ 7.52 – 7.25 (m, 18H, Ph), 7.24 – 7.13 (m, 4H, Ph), 6.37 (t, *J* = 53.7 Hz, 1H*, CF_2H), 6.31 (t, *J* = 53.6 Hz, 1H, CF_2H), 4.68 (s, 2H*, $PhCH_2NCO$), 4.63 (s, 2H, $PhCH_2NCO$), 3.87 (s, 2H*, $PhCH_2$), 3.75 (s, 2H, $PhCH_2$), 3.53 (t, *J* = 6.2 Hz, 2H*, $CONCH_2CH_2$), 3.52 – 3.45 (m, 2H, $CONCH_2CH_2$), 2.85 (t, *J* = 6.2 Hz, 2H*, $CONCH_2CH_2$), 2.80 (t, *J* = 6.6 Hz, 2H, $CONCH_2CH_2$).

^{1}H NMR (500 MHz, wet $CDCl_3$ as ~1.1:1 mixture of rotamers; major rotamer denoted by *): δ 7.40 – 7.15 (m, 20H, Ph), 6.37 (t, *J* = 53.8 Hz, 1H, CF_2H), 6.17 (t, J = 53.6 Hz, 1H*, CF_2H), 4.70 (s, 2H*, $PhCH_2NCO$), 4.64 (s, 2H, $PhCH_2NCO$), 3.75 (s, 2H, $PhCH_2$), 3.74 (s, 2H*, $PhCH_2$), 3.45 (2 x t, *J* = 6.5 Hz, 2H* + 2H), 2.79 (2 x t, *J* = 6.5, 2H*+2H), 1.44 (bs, 2H, XH).

^{13}C NMR (125 MHz, $CDCl_3$ as ~1.1:1 mixture of rotamers;): δ 163.31 (t, $^2J_{CF}$ = 25.1 Hz), 162.84 (t, $^2J_{CF}$ = 25.1 Hz), 136.02, 135.01, 128.99, 128.88, 128.79, 128.69, 128.57, 128.19, 128.13, 127.91, 127.81, 127.39, 127.36, 109.78 (t, *J* = 251.9 Hz), 109.49 (t, $^4J_{CF}$ = 251.8 Hz), 53.60, 53.41, 52.54, 50.49 (t, $^4J_{CF}$ = 3.4 Hz), 49.03, 46.51, 45.74, 44.94, 44.10.

^{19}F NMR (565 MHz, $CDCl_3$): δ -119.72 – -121.05 (m), -121.46 (d, $^2J_{HF}$ = 53.8 Hz), -122.05 (d, $^2J_{HF}$ = 53.8 Hz).

^{19}F NMR (565 MHz, wet $CDCl_3$): δ -120.33 (d, $^2J_{HF}$ = 53.7 Hz), -122.02 (d, $^2J_{HF}$ = 54.2 Hz).

HRMS (ESI$^+$): calcd. for $[C_{18}H_{20}F_2N_2O+H]^+$ ($[M+H]^+$): *m/z* 319.1622; found 319.1609.

N,N'-(Ethane-1,2-diyl)bis(*N*-benzyl-2,2-difluoroacetamide) (3)

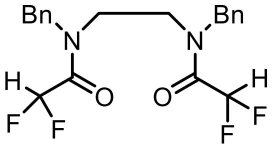

To a solution of *N,N'*-dibenzylethylenediamine (120 mg, 0.5 mmol) and anhydrous pyridine (300 µL) in DCM (2 mL) at 0 °C was added difluoroacetic anhydride (350 µL, 2 mmol, 4 eq.) dropwise. A precipitate formed which dissolved upon complete addition of the reagent. The mixture was stirred at rt overnight (crude mixture ^{1}H NMR showed pyridinium NH proton at 15.34 ppm in $CDCl_3$); then diluted with DCM and quenched with sat. aq. $NaHCO_3$. The mixture was extracted with DCM (3 x 15 mL), washed with sat. aq. NaCl (15 mL) and sat. aq. $KHSO_4$ (15 mL). The organic phase was separated and dried over Na_2SO_4. The solvents were evaporated under reduced pressure to give bis(amide) **3** as a colourless oil in quantitative yield as a mixture of rotamers which solidified to a white solid upon standing at rt.

^{1}H NMR (600 MHz, $CDCl_3$, mixture of 3 conformers; * denotes conformer **3b**; ** conformer **3c**): δ 7.47 – 7.27 (m, 7H), 7.26 – 7.20 (m, 3H), 6.21 (t, $^2J_{HF}$ = 53.5 Hz, 1H), 6.16 (t, $^2J_{HF}$ = 53.7 Hz, 1H*), 6.15 (t, $^2J_{HF}$ = 53.7 Hz, 1H**), 6.11 (t, $^2J_{HF}$ = 53.5 Hz, 1H), 4.71 (s, 4H*), 4.61 (s, 4H**), 4.59 (s, 2H), 4.56 (s, 2H), 3.57 (s, 2H**), 3.51 (s, 2H*), 3.43 (dd, *J* = 9.3, 5.8 Hz, 2H), 3.34 (dd, *J* = 9.3, 5.8 Hz, 2H).

^{13}C NMR (125 MHz, $CDCl_3$): δ 162.86 (t, $^2J_{CF}$ = 25.1 Hz), 162.62 (t, $^2J_{CF}$ = 25.4 Hz), 162.36 (t, $^2J_{CF}$ = 25.8 Hz), 135.70, 135.48, 134.97, 134.62, 129.11, 128.98, 128.89, 128.75, 128.58, 128.37, 128.18, 127.96, 127.94, 127.69, 127.27, 110.88 (t, $^1J_{CF}$ = 255.3 Hz), 110.23 (t, $^1J_{CF}$ = 253.8 Hz), 109.64 (t, $^1J_{CF}$ = 252.8 Hz), 109.39 (t, $^1J_{CF}$ = 252.5 Hz), 51.31 (t, $^4J_{CF}$ = 3.9 Hz), 50.03 (t, $^4J_{CF}$ = 3.6 Hz), 49.59, 49.13, 44.83, 44.04, 42.28 (t, $^4J_{CF}$ = 3.1 Hz), 41.91.

^{19}F NMR (377 MHz, $CDCl_3$): δ -119.95 (d, *J* = 53.4 Hz), -120.29 (d, *J* = 53.8 Hz**), -120.71 (d, *J* = 53.6 Hz*), -121.22 (d, *J* = 53.6 Hz).

^{19}F{^{1}H} NMR (377 MHz, $CDCl_3$): δ -119.95 (s), -120.29 (s)**, -120.71 (s)*, -121.22 (s).

IR (cm^{-1}): 1667, 1661, 1481, 1446, 1367, 1280, 1117, 1056, 725.

HRMS (ESI$^+$): calcd. for $[C_{20}H_{20}F_4N_2O_2+H]^+$ ($[M+H]^+$): *m/z* 397.1539; found 397.1519.

***N*-benzyl-*N*-(2-(1-benzyl-3-(4-methoxyphenyl)ureido)ethyl)-2,2-difluoroacetamide (6)**

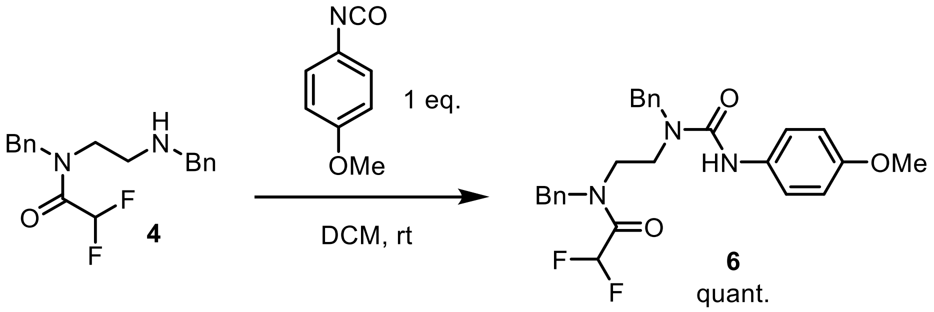

To a solution of **4** (58 mg, 0.18 mmol) in DCM (1 mL) at 0 °C was added a solution of 4-methoxyphenylisocyanate (27 mg, 0.18 mmol, 1 eq.; filtered to remove urea impurity) in DCM (1 mL) and the mixture was stirred for 10 min at 0 °C and then at rt overnight. The solvent was evaporated to give the product in quantitative yield as white crystalline material. Silica gel column chromatography (DCM/MeOH 96:4) did not lead to further purification of **6** due to decomposition.

^{1}H NMR (500 MHz, $CDCl_3$): δ δ 7.83 (s, 1H, NH), 7.52 – 7.45 (m, 2H, ArH), 7.43 – 7.34 (m, 4H, ArH), 7.32 – 7.25 (m, 2H, *o*-ArH), 7.17 – 7.12 (m, 4H, ArH), 6.90 – 6.82 (m, 2H), 6.22 (t, *J* = 53.5 Hz, 1H, CF_2H), 4.56 (s, 2H, $PhCH_2$), 4.43 (s, 2H, $PhCH_2$)), 3.78 (s, 3H, OMe), 3.22 (dd, *J* = 9.6, 5.9 Hz, 2H), 3.14 – 3.07 (m, 2H).

^{13}C NMR (126 MHz, $CDCl_3$): δ 163.07 (t, $^2J_{CF}$ = 25.2 Hz), 155.51, 155.39, 138.14, 134.58, 132.87, 129.10, 128.66, 128.61, 128.04, 128.01, 127.55, 121.44, 113.99, 110.55 (t, $^1J_{CF}$ = 254.4 Hz), 55.48, 51.60 (t, $^4J_{CF}$ = 4.0 Hz), 50.87, 45.68, 43.39.

^{19}F NMR (377 MHz, $CDCl_3$): δ -119.73 (d, *J* = 53.7 Hz).

^{19}F{^{1}H} NMR (377 MHz, $CDCl_3$): δ -119.73 (s),

IR (cm^{-1}): 3336, 3029, 2999, 2955, 2834, 1656, 1645, 1509, 1315, 1237, 1134, 1057, 1039, 823.

HRMS (ESI$^+$): calcd. for $[C_{26}H_{27}F_2N_3O_3+H]^+$ ($[M+H]^+$): *m/z* 468.2099; found 468.2075.

5. Structural Identification

Assignment of Chemical Shifts

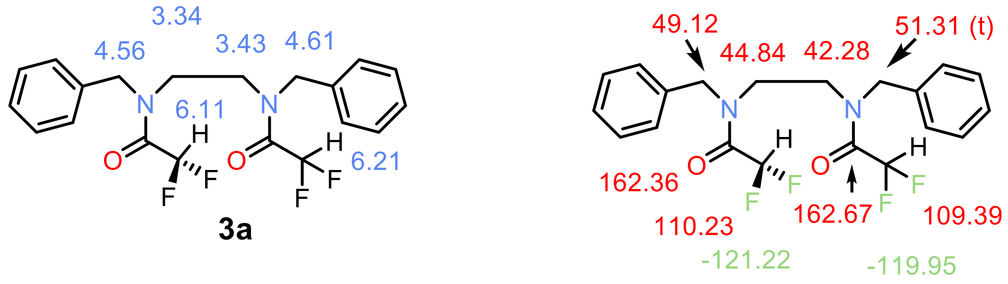

Figure 1. Assignment of ^{1}H and $^{13}C/^{19}F$ NMR shifts ($CDCl_3$) of ***E,Z*-3a** at 298 K.

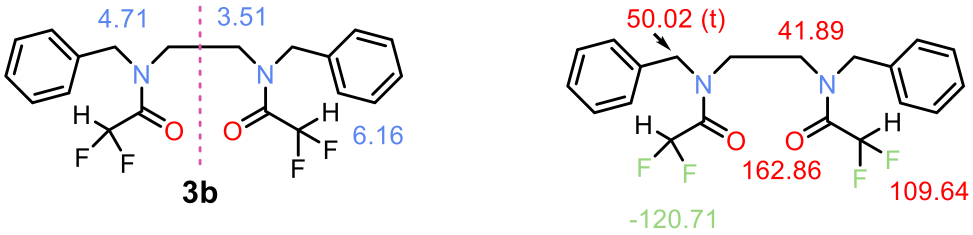

Figure 2. Assignment of ^{1}H and $^{13}C/^{19}F$ NMR shifts ($CDCl_3$) of ***Z,Z*-3b** at 298 K.

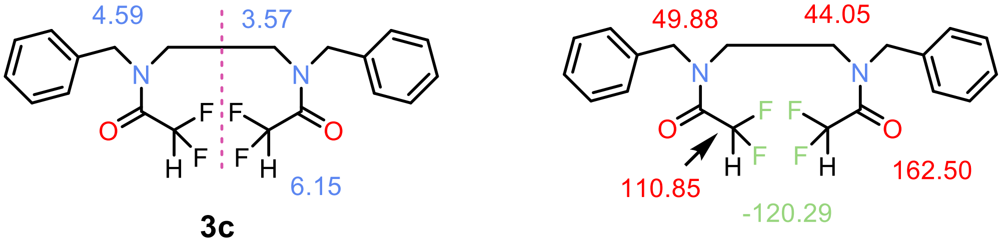

Figure 3. Assignment of ^{1}H and $^{13}C/^{19}F$ NMR shifts ($CDCl_3$) of ***E,E*-3c** at 298 K.

The conformational flexibility and the population of multiple conformations preclude the use of quantitative $^{1}H,^{19}F$-HOESY experiments.[2,3] However, $^{19}F,^{13}C$-correlation experiments proved useful.[4]

NMR Spectra

Compound 3

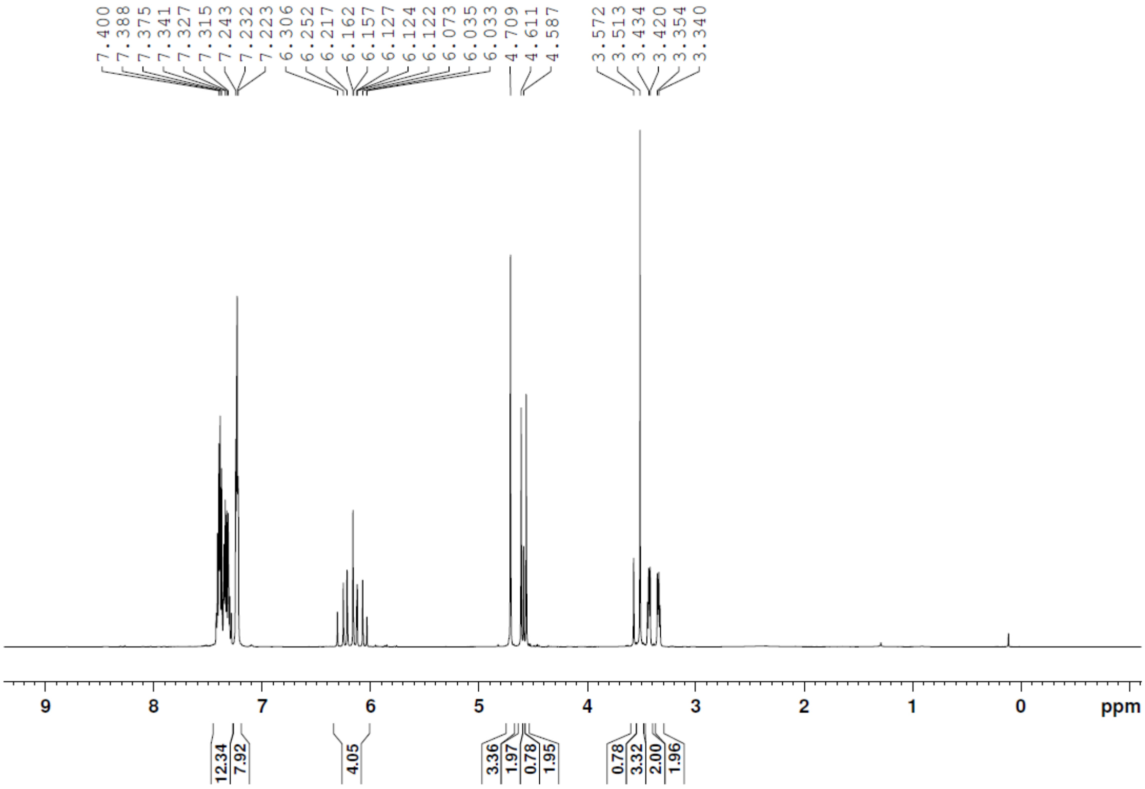

Figure 4. ^1H NMR spectrum (600 MHz, $CDCl_3$) of **3** at 298 K.

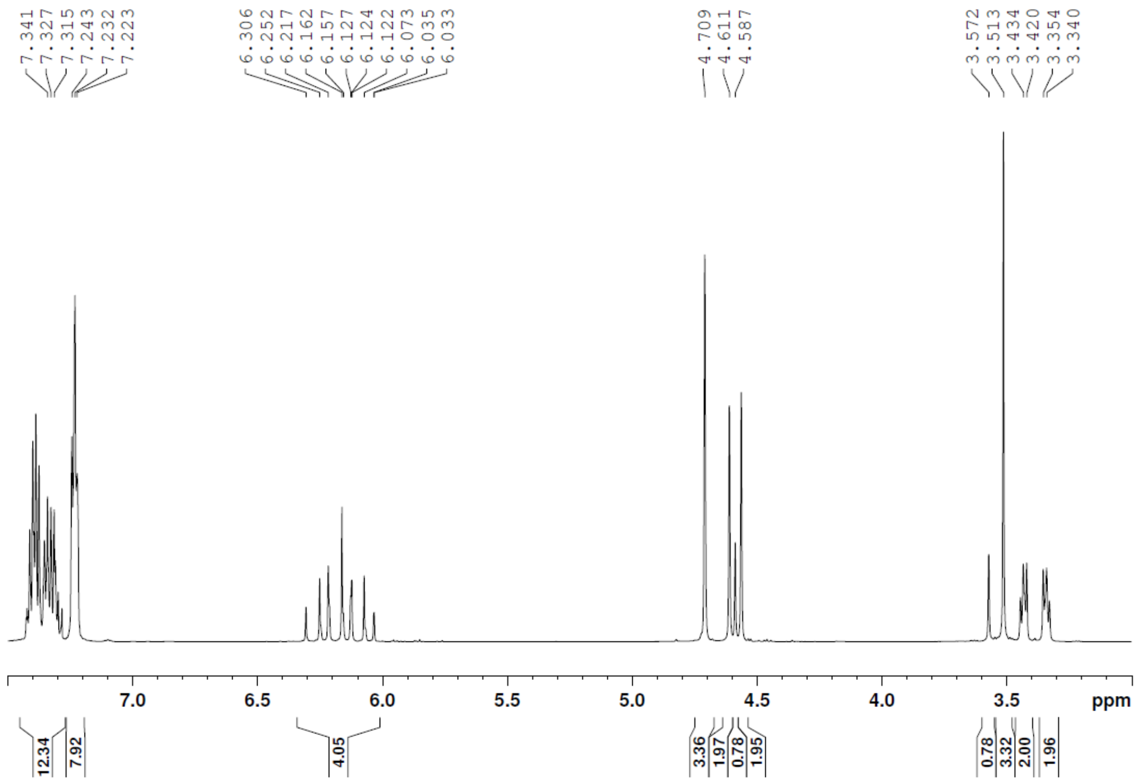

Figure 5. Portion of ^1H NMR spectrum (600 MHz, $CDCl_3$) of **3** at 298 K.

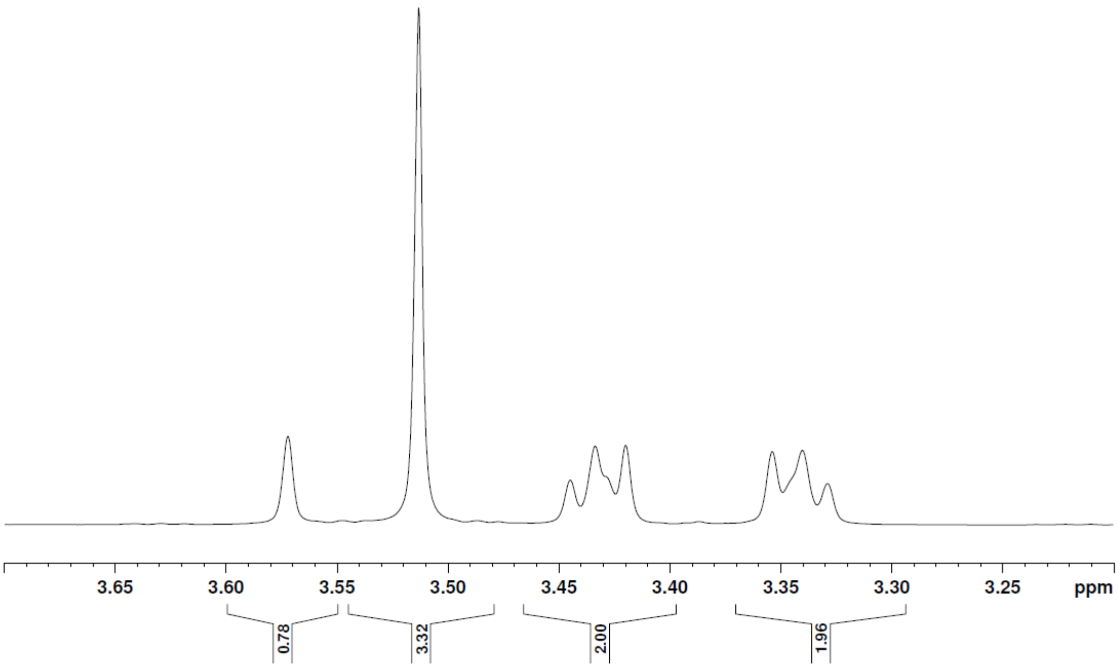

Figure 6. Portion of ^{1}H NMR spectrum (600 MHz, $CDCl_3$) of **3** at 298 K.

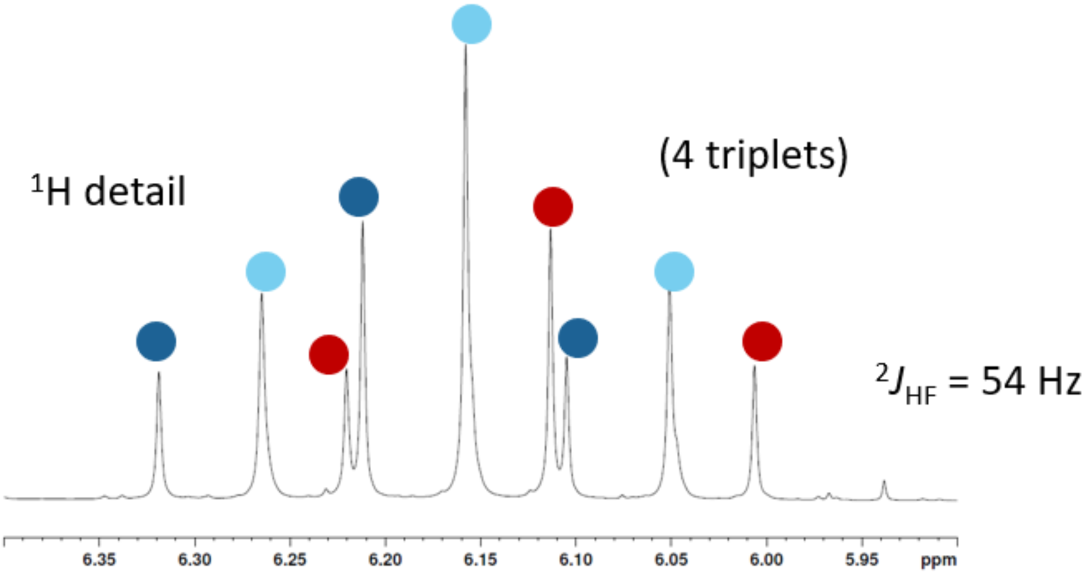

Figure 7. Portion of ^{1}H NMR spectrum (600 MHz, $CDCl_3$, CF_2H protons) of **3** at 298 K.

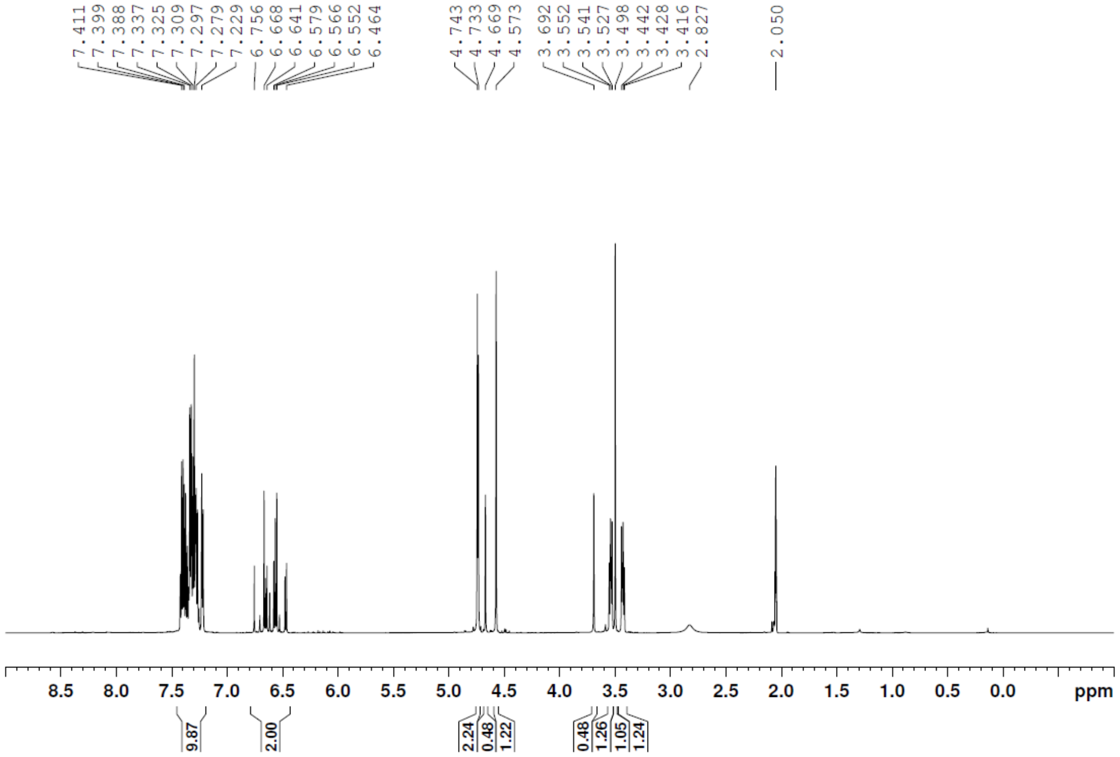

Figure 8. Portion of ^{1}H NMR spectrum (600 MHz, acetone-d_6) of **3** at 298 K.

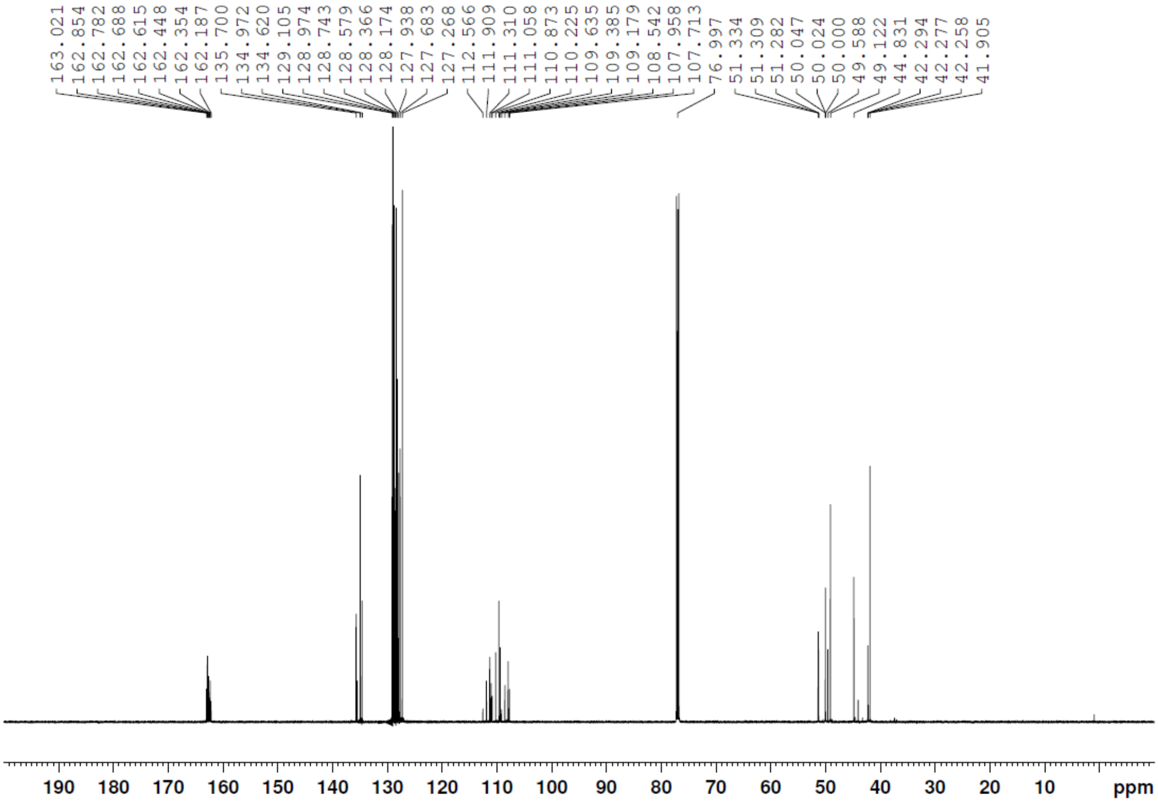

Figure 9. $^{13}C\{^{1}H\}$ NMR spectrum (151 MHz, $CDCl_3$) of **3** at 298 K.

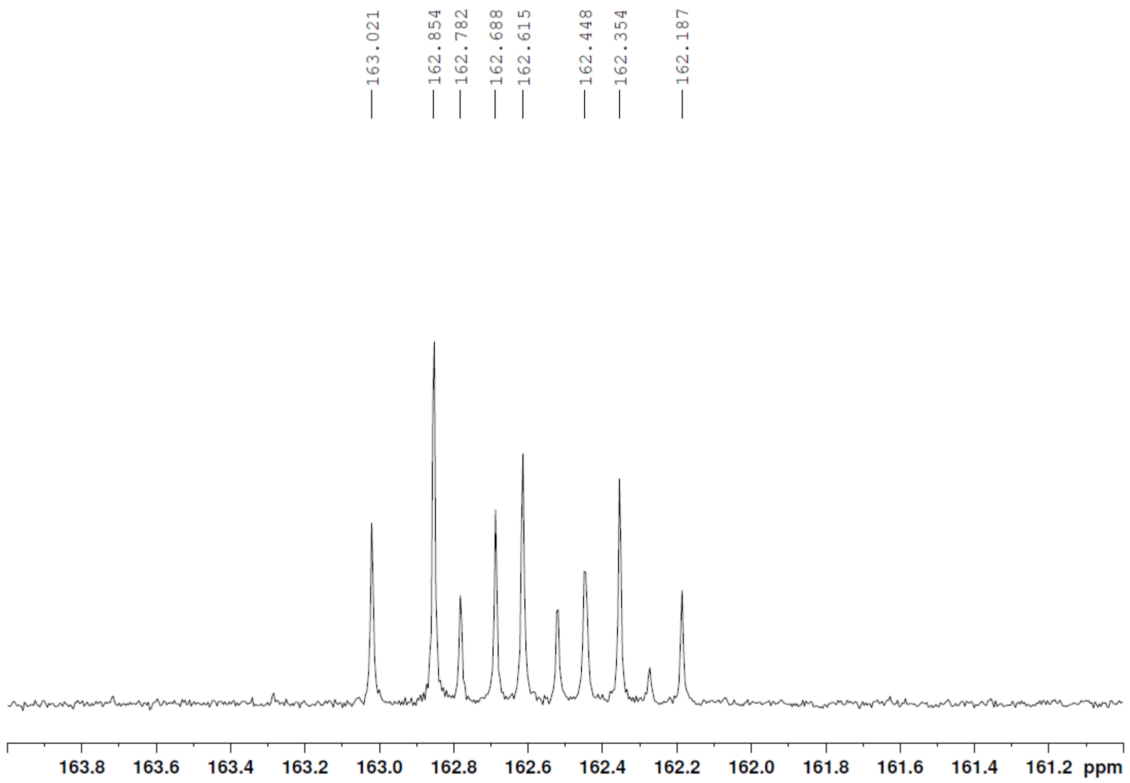

Figure 10. Portion of $^{13}C\{^{1}H\}$ NMR spectrum (151 MHz, $CDCl_3$) of **3** at 298 K (difluoroacetamide carbonyl).

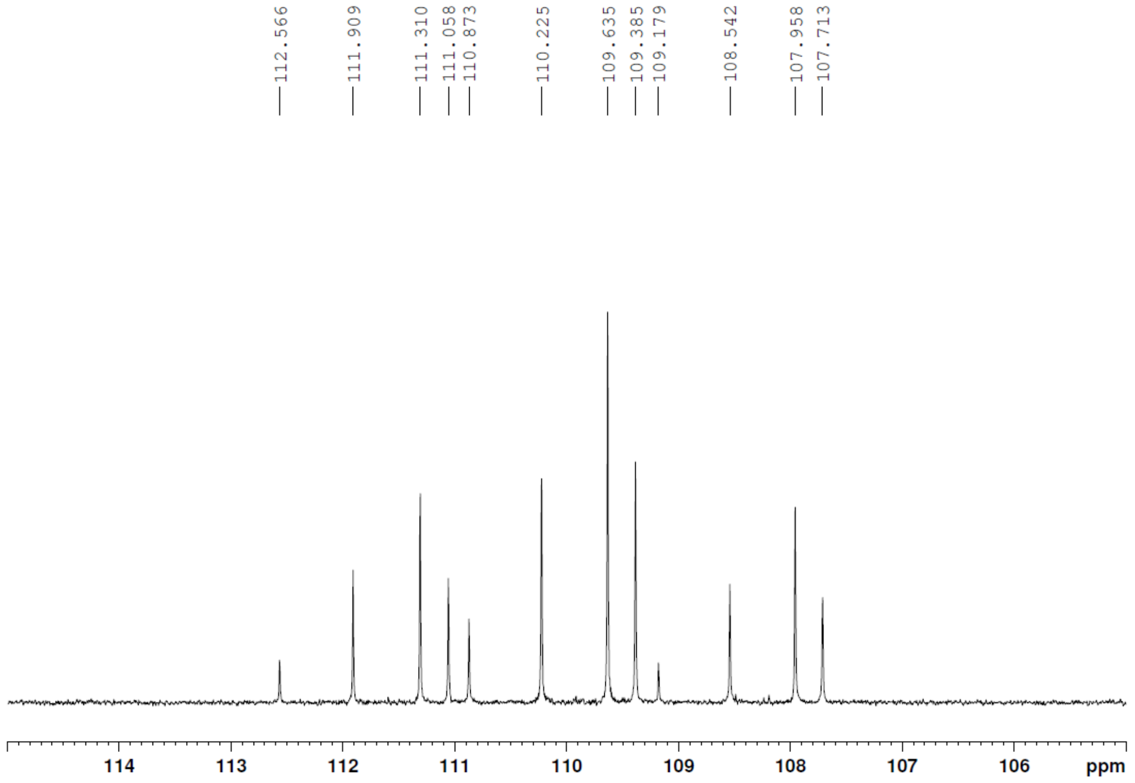

Figure 11. Portion of $^{13}C\{^{1}H\}$ NMR spectrum (151 MHz, $CDCl_3$) of **3** at 298 K (CF_2H region).

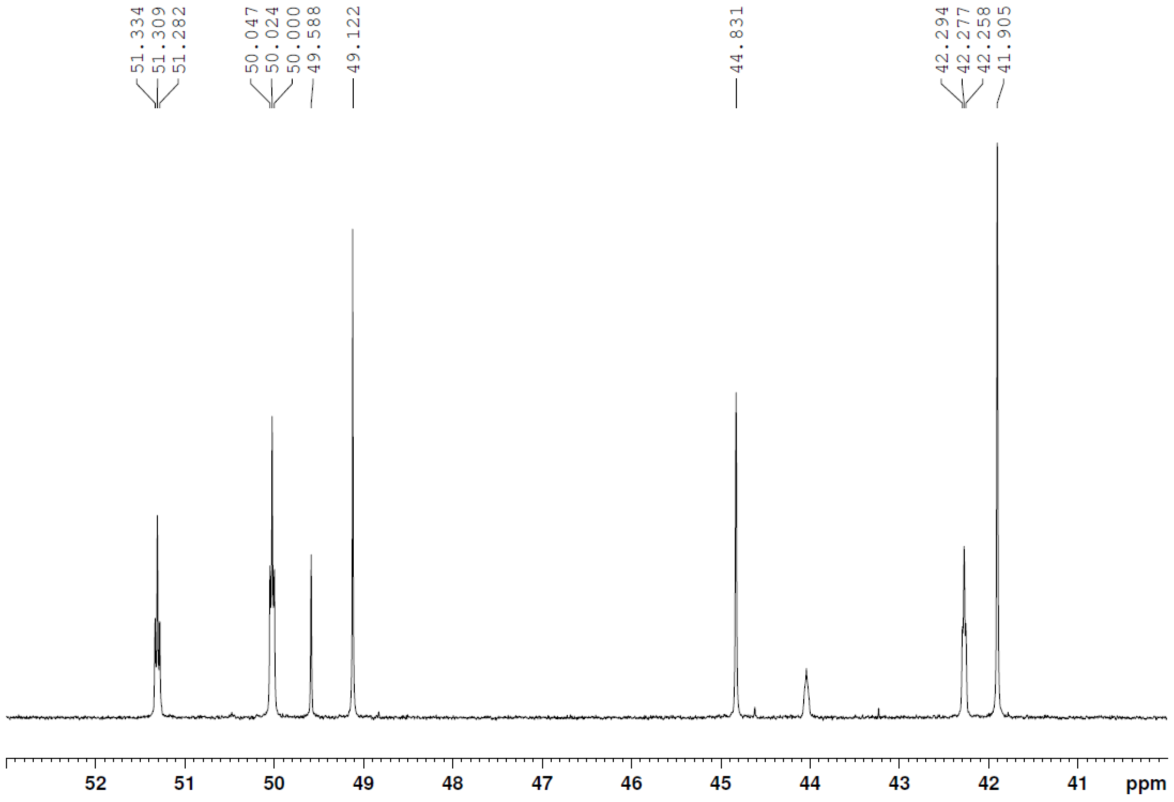

Figure 12. Portion of $^{13}C\{^{1}H\}$ NMR spectrum (151 MHz, $CDCl_3$) of **3** at 298 K (aliphatic region).

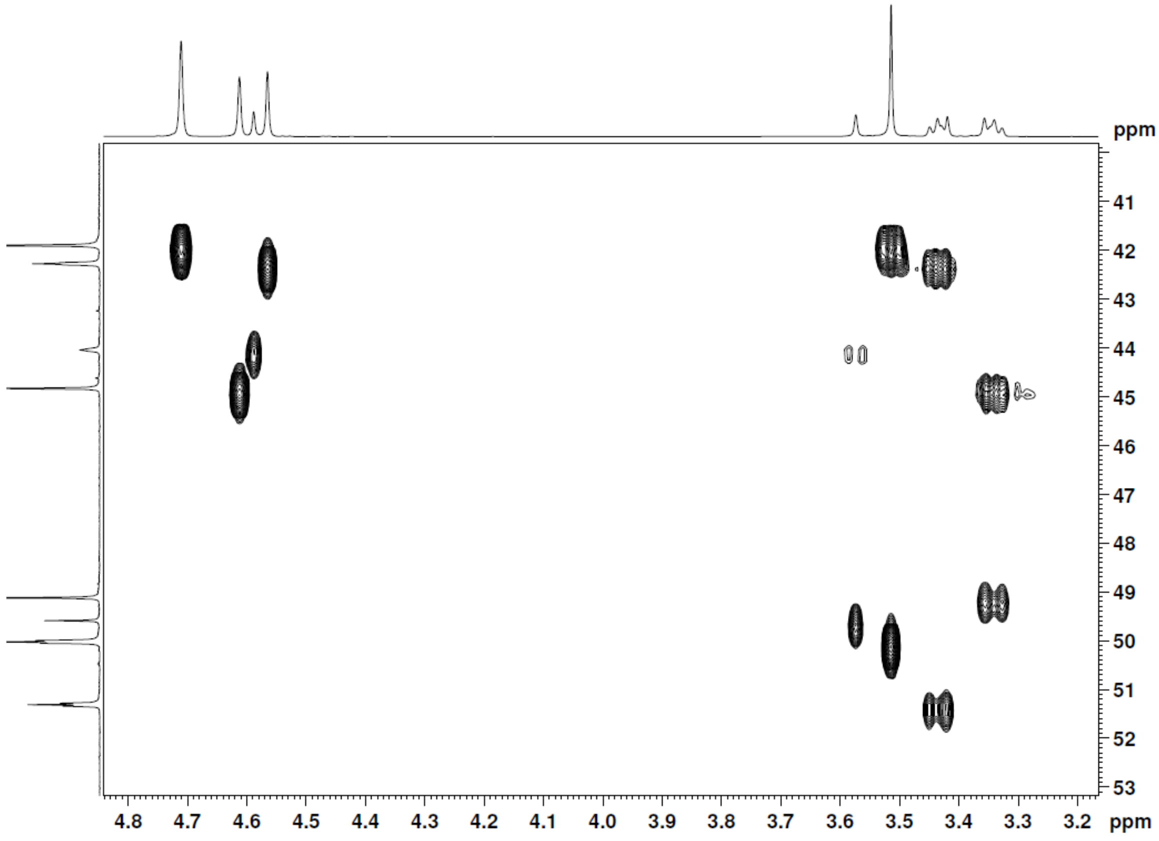

Figure 13. Portion of $^{1}H,^{13}C$-HMBC NMR spectrum (600 MHz, $CDCl_3$) of **3** at 298 K showing correlations it the aliphatic region (benzylic and ethylene bridge).

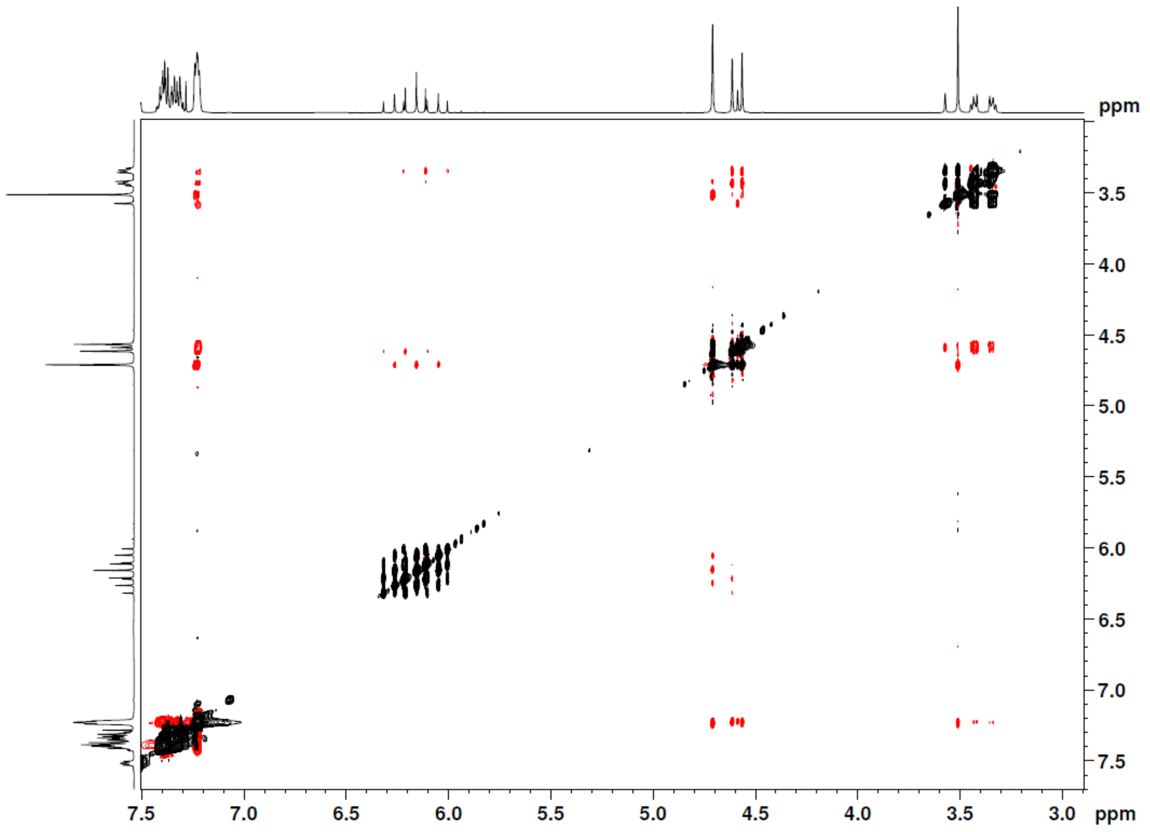

Figure 14. ^{1}H,^{1}H-NOESY NMR spectrum (600 MHz, $CDCl_3$, mixing time 500 ms) of **3** at 298 K showing NOE correlations (red crosspeaks) as well as exchange crosspeaks (black) between isomers **3a – c.**

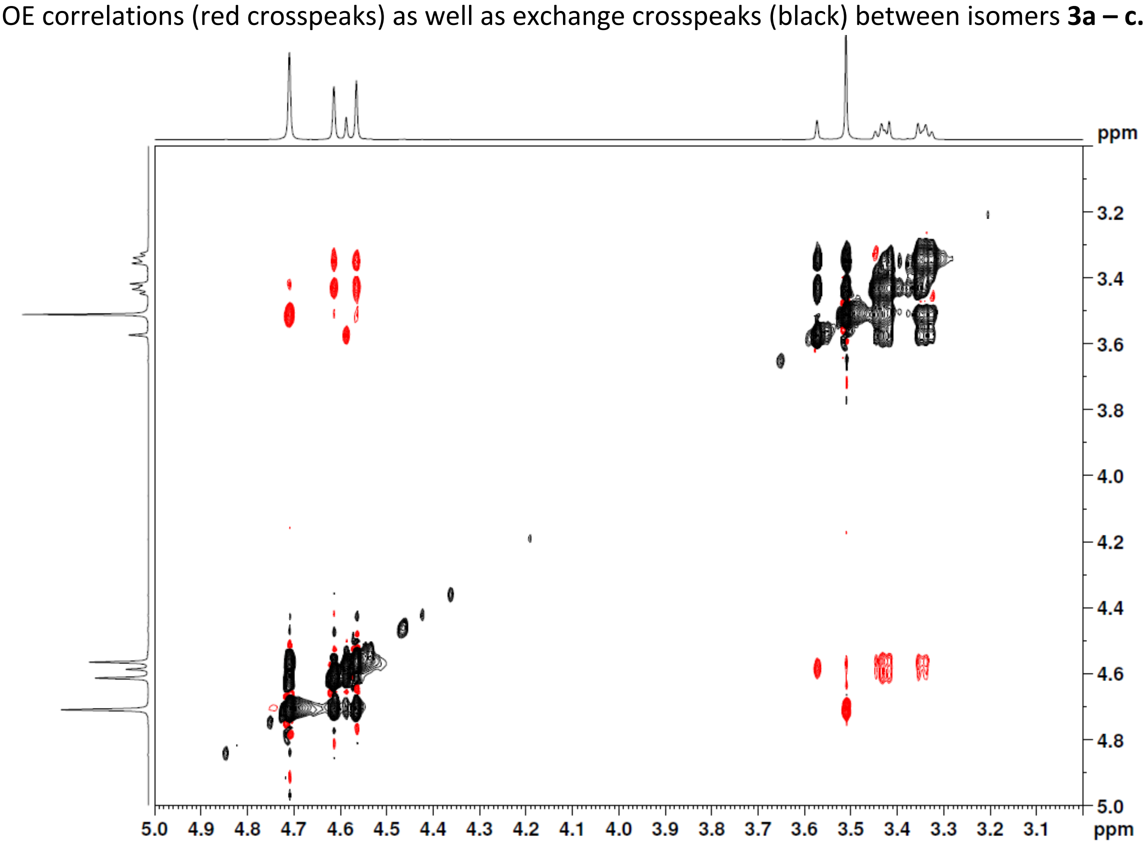

Figure 15. Portion of ^{1}H,^{1}H-NOESY NMR spectrum (600 MHz, $CDCl_3$) of **3** at 298 K.

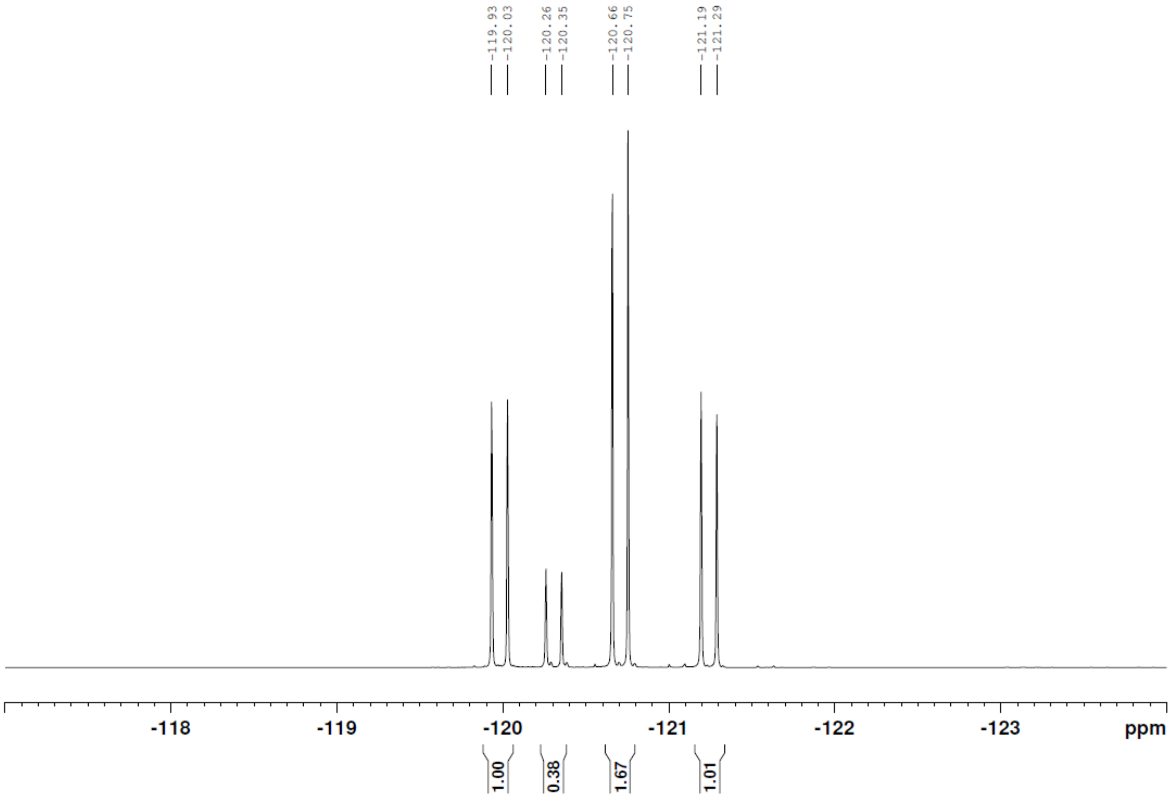

Figure 16. ^{19}F NMR spectrum (565 MHz, $CDCl_3$) of **3** at 298 K.

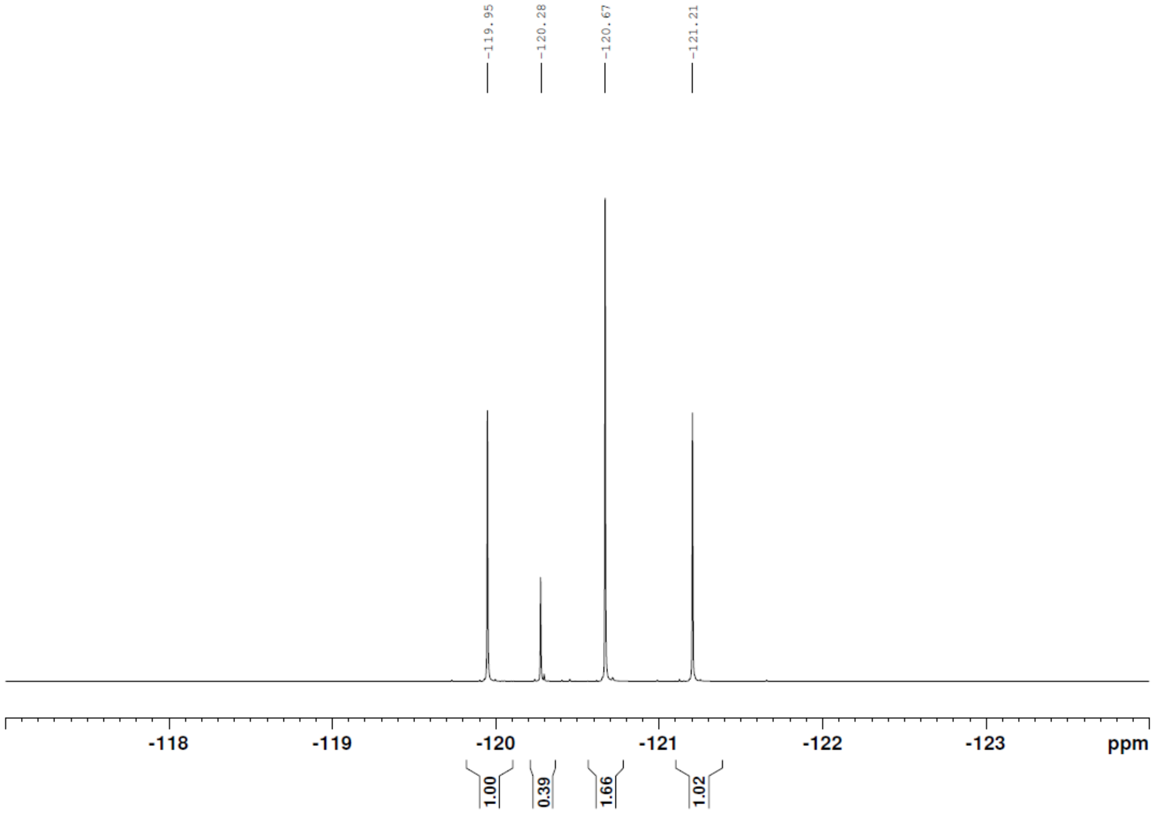

Figure 17. ^{19}F{^{1}H} NMR spectrum (377 MHz, $CDCl_3$) of **3** at 298 K.

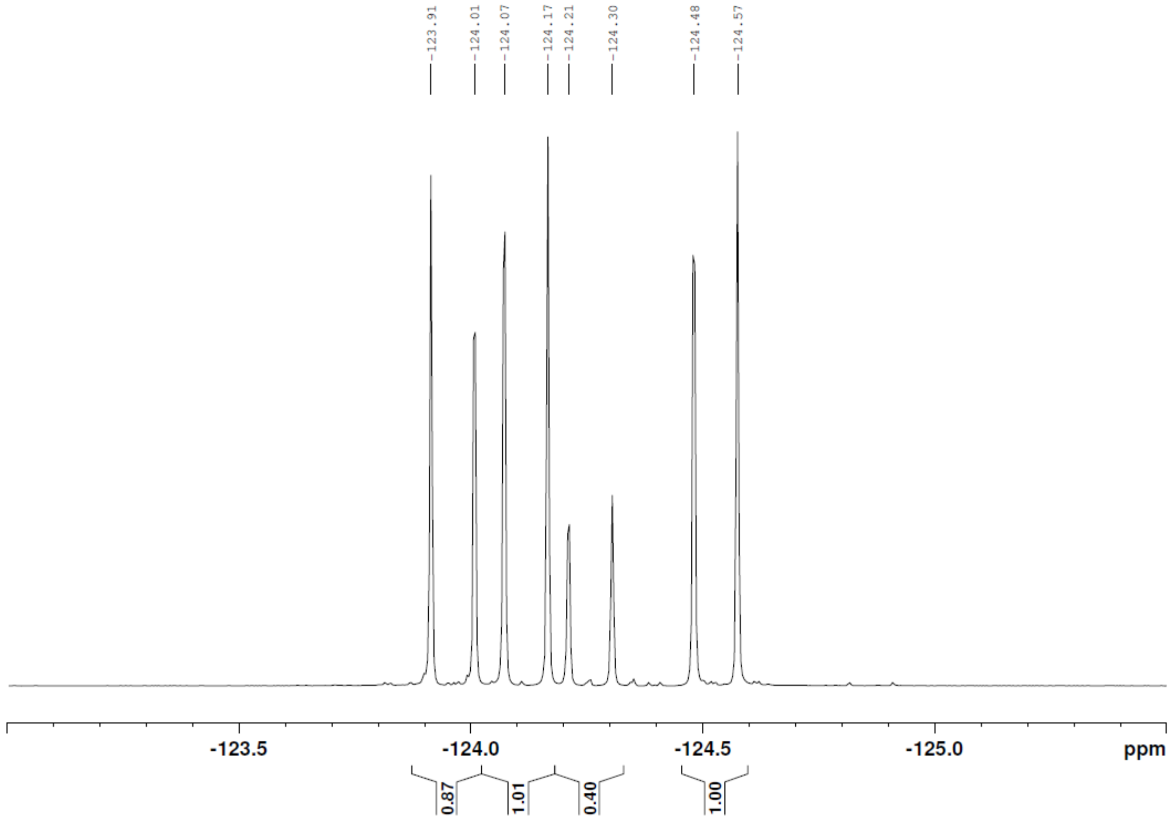

Figure 18. ^{19}F NMR spectrum (565 MHz, acetone-d_6) of **3** at 298 K.

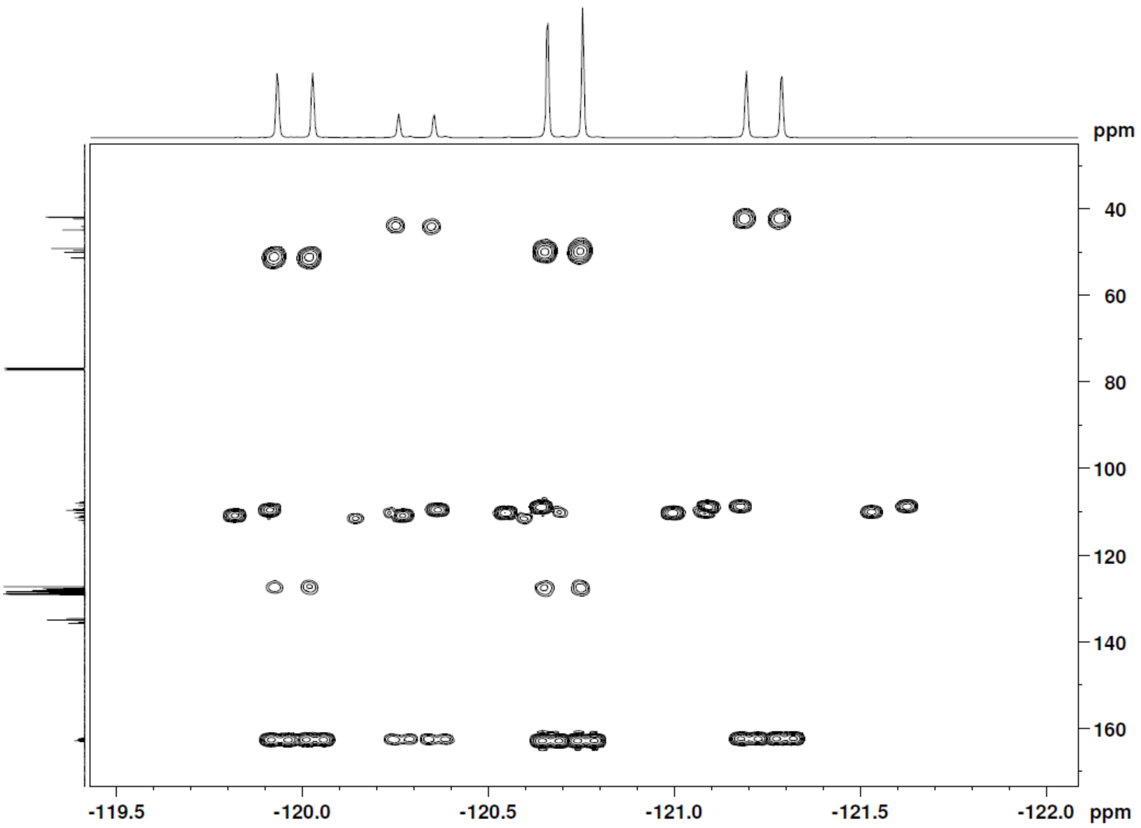

Figure 19. $^{19}F,^{13}C$-HMBC NMR spectrum (565 MHz, $CDCl_3$, optimised for 5 Hz) of **3** at 298 K.

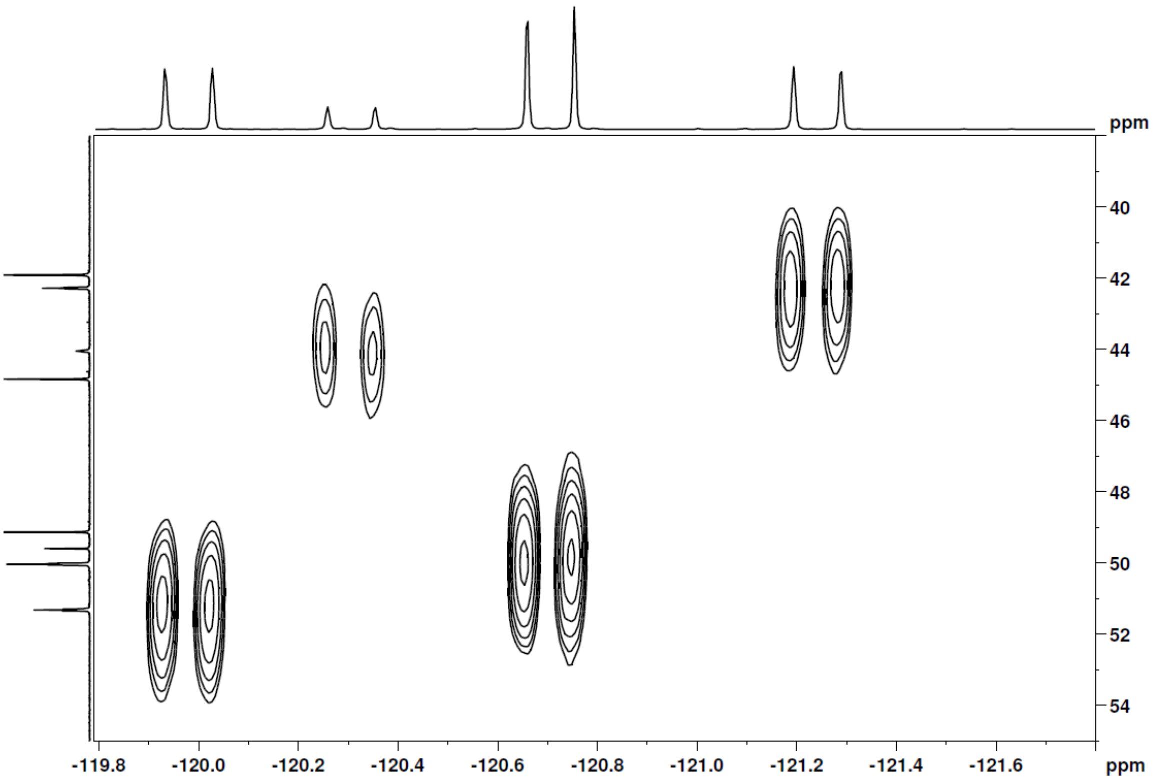

Figure 20. Portion of $^{19}F,^{13}C$-HMBC NMR spectrum (565 MHz, $CDCl_3$) of **3** at 298 K showing correlations it the aliphatic region (benzylic and ethylene bridge).

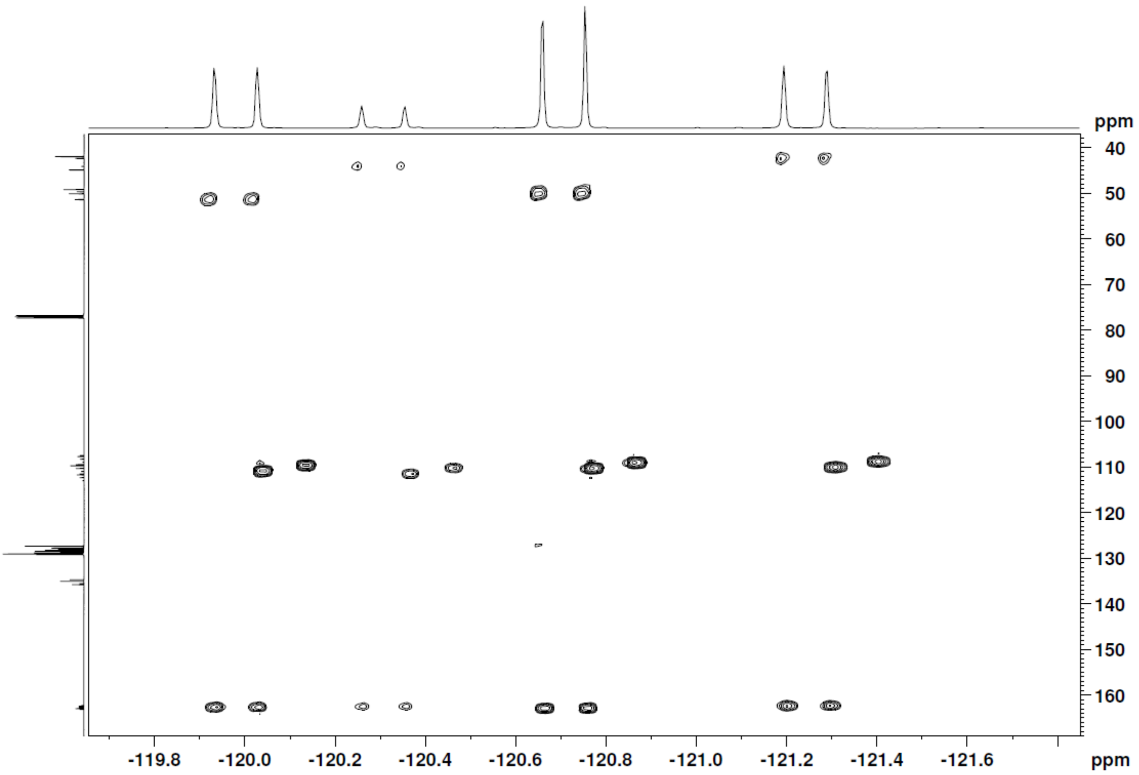

Figure 21. $^{19}F,^{13}C$-HSQC NMR spectrum (565 MHz, $CDCl_3$, optimised for 10 Hz) of **3** at 298 K.

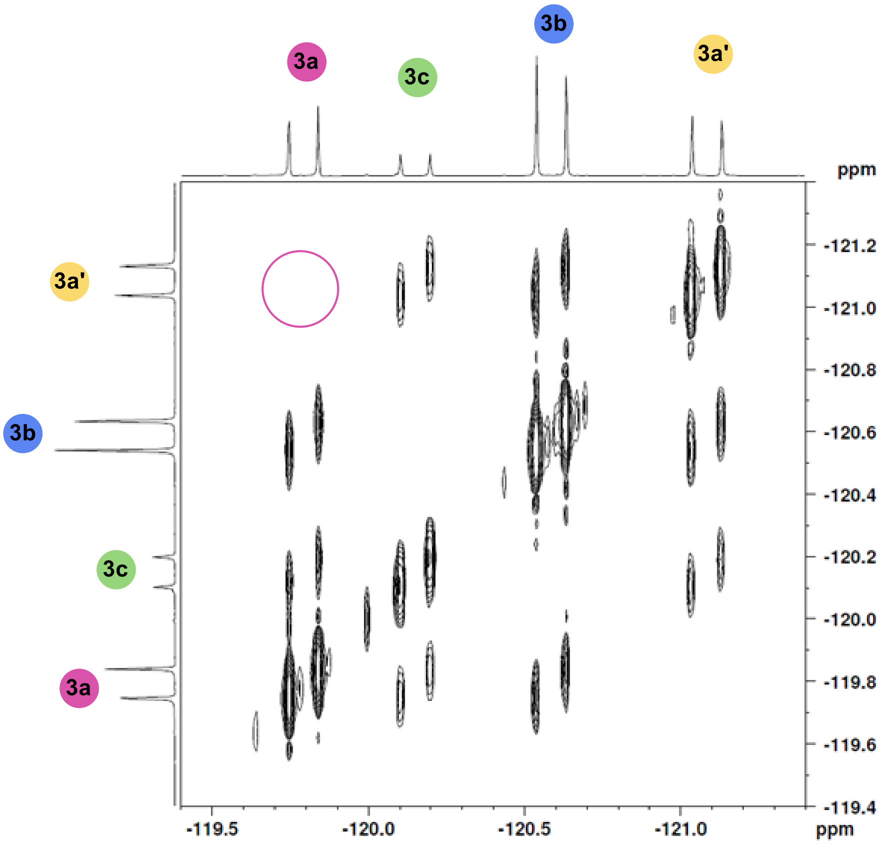

Figure 22. Annotated $^{19}F,^{19}F$-EXSY NMR spectrum (565 MHz, $CDCl_3$, mixing time 500 ms) of **3** at 298 K. Absent or very weak crosspeak between **3a** and **3a'** suggests extremely slow correlated rotation.

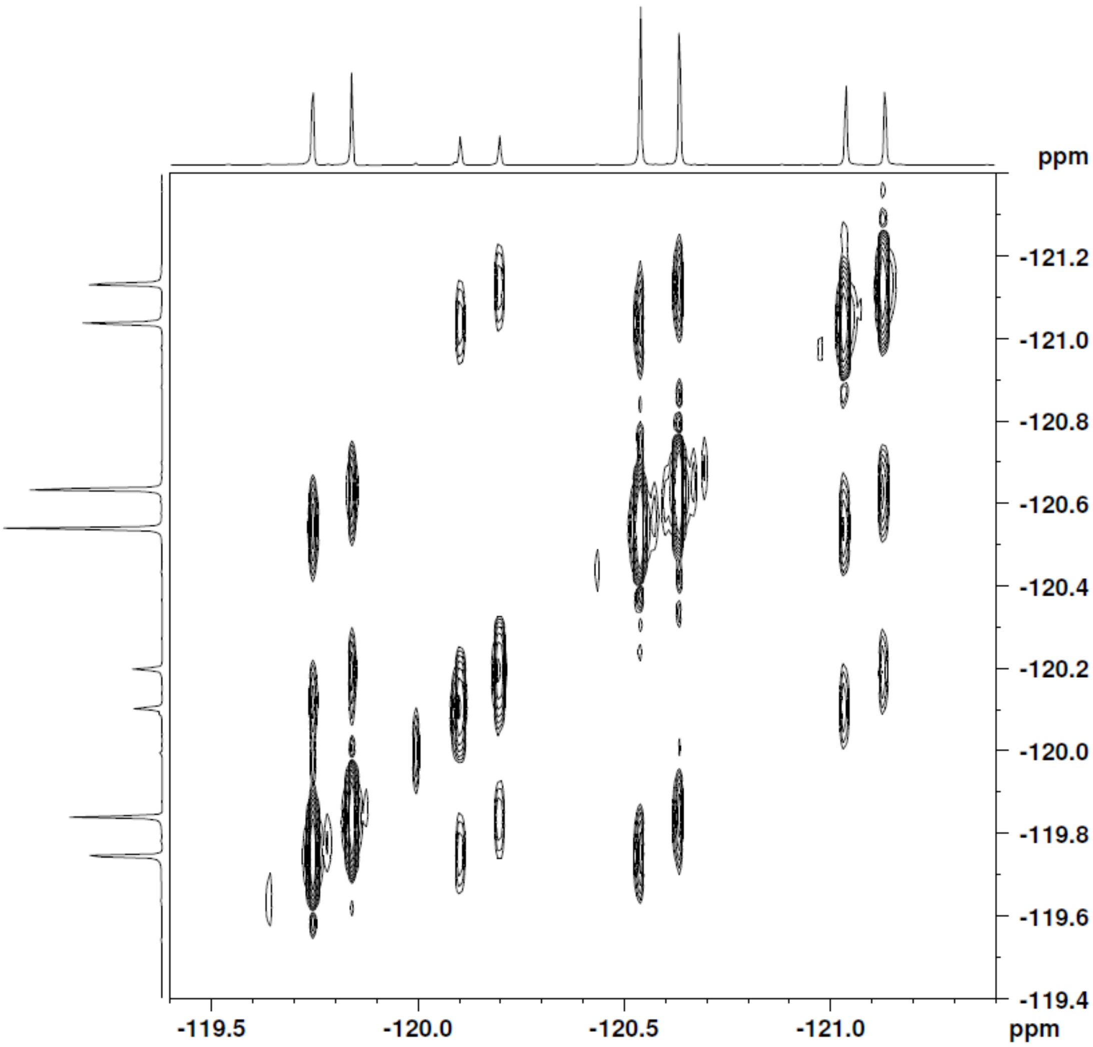

Figure 23. $^{19}F,^{19}F$-EXSY NMR spectrum (565 MHz, $CDCl_3$, mixing time 500 ms) of **3** at 298 K.

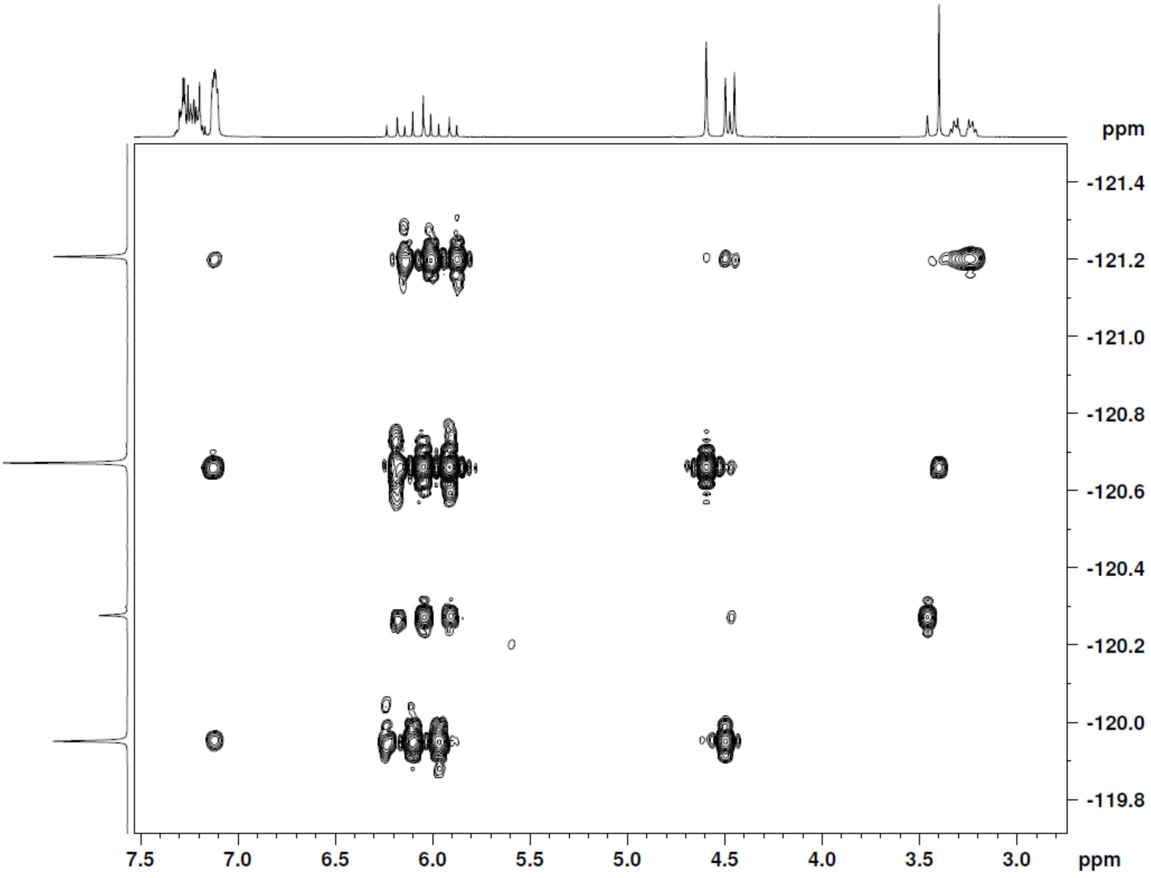

Figure 24. ^{1H}F,^{19}F-HOESY NMR spectrum (400 MHz, $CDCl_3$, mixing time 300 ms) of **3** at 298 K.

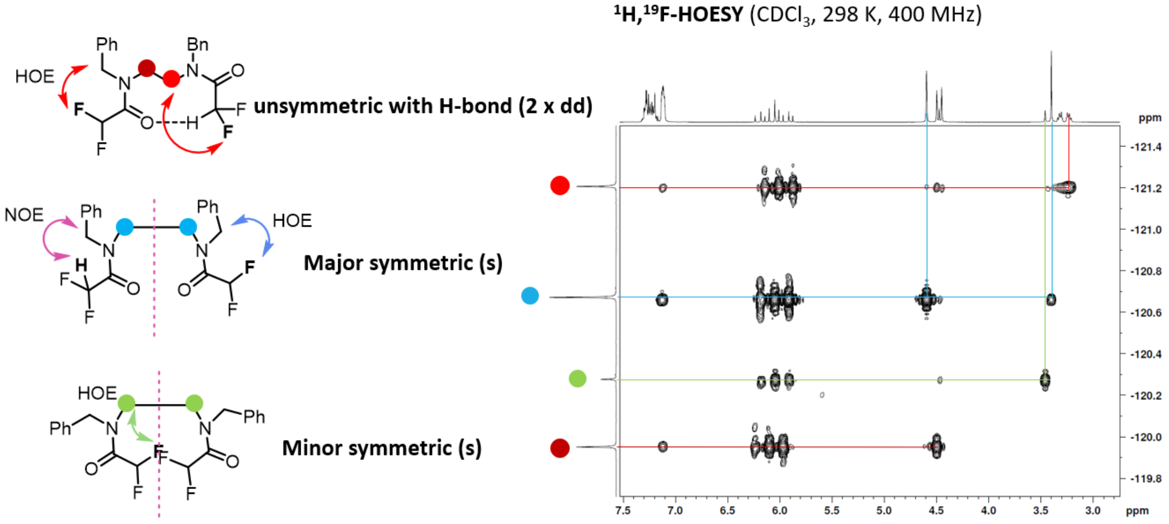

Figure 25. ^{1H}F,^{19}F-HOESY NMR spectrum showing three conformations **3a – c.**

Compound 4

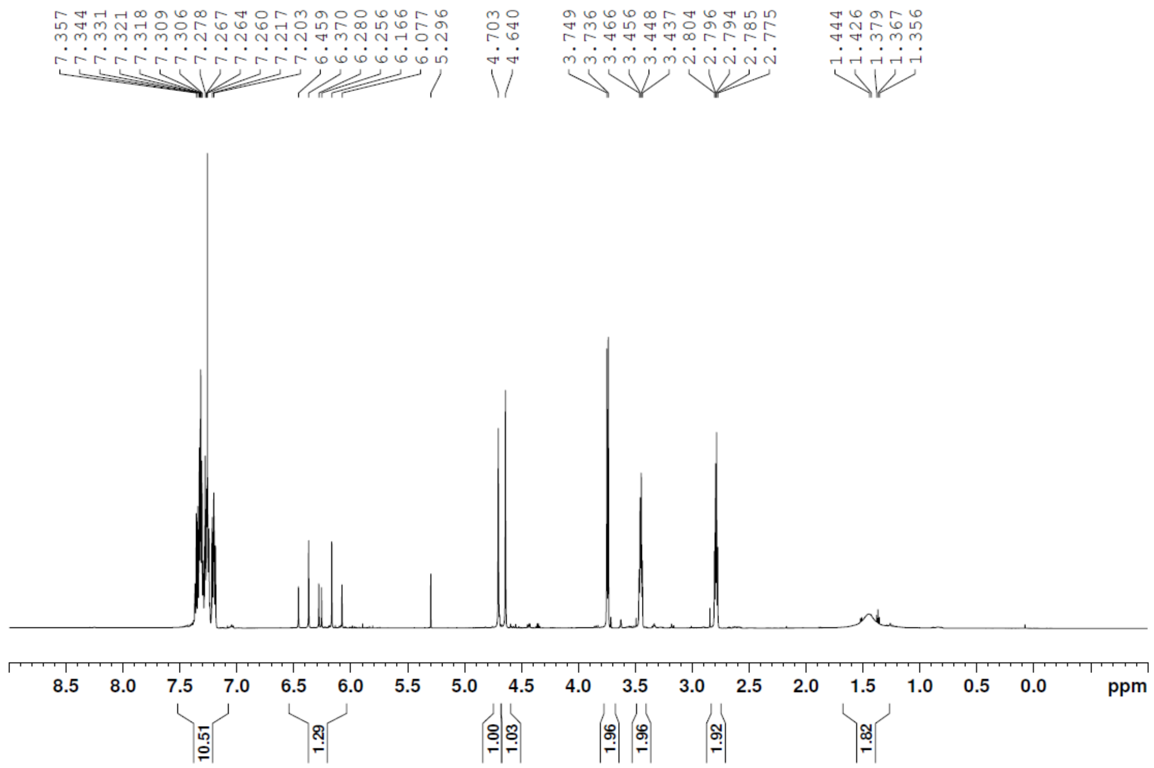

Figure 26. ^{1}H NMR spectrum (600 MHz, wet $CDCl_3$) of **4** at 298 K.

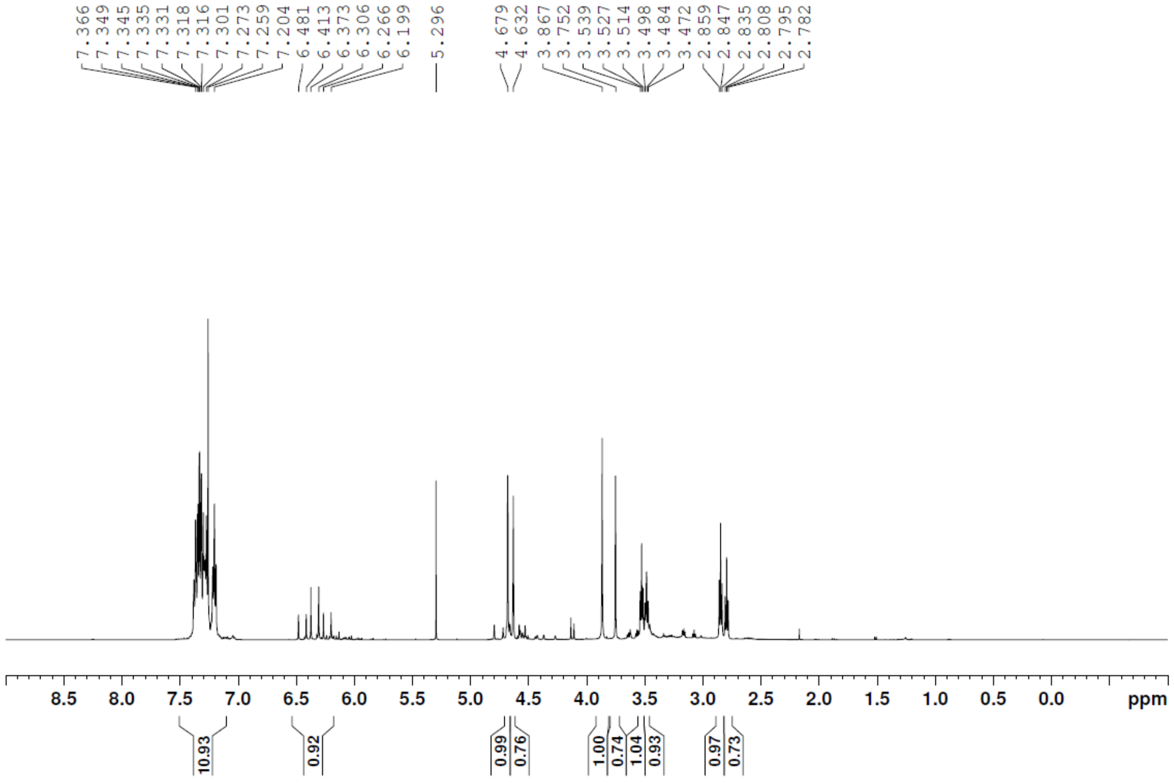

Figure 27. ^{1}H NMR spectrum (600 MHz, $CDCl_3$) of **4** at 298 K.

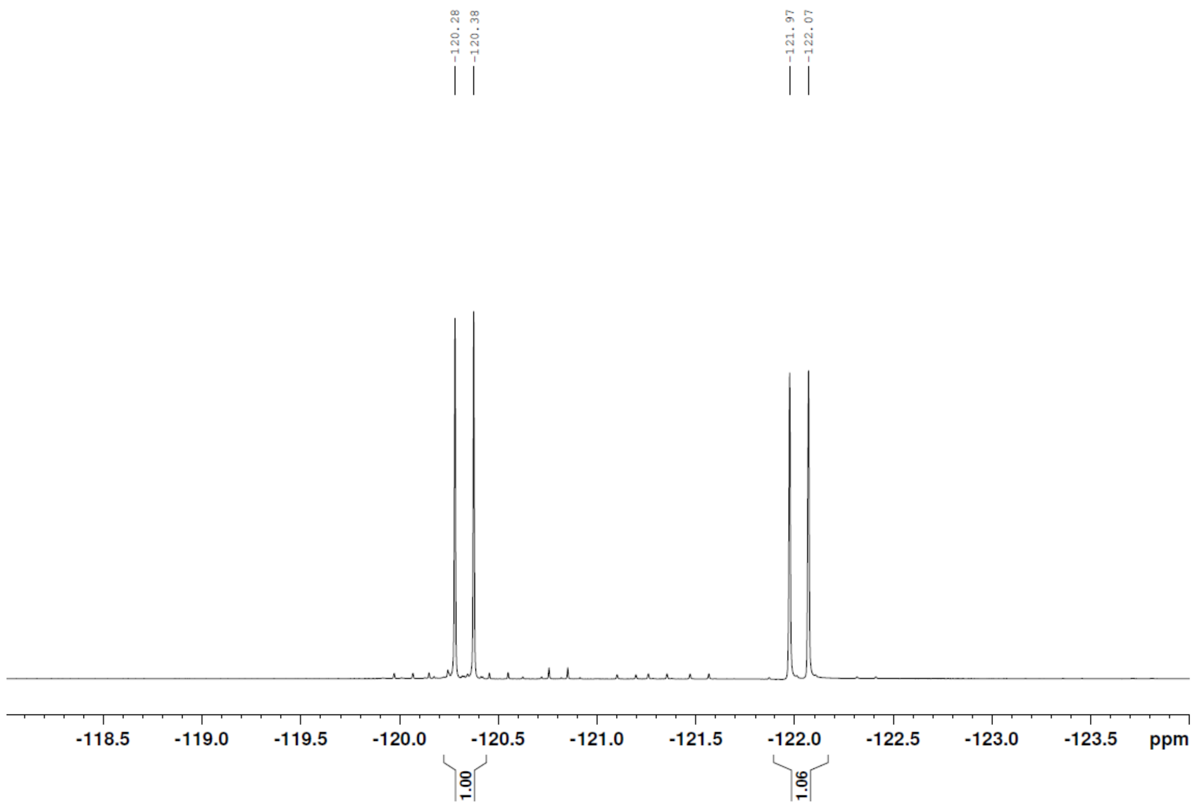

Figure 28. ^{19}F NMR spectrum (565 MHz, wet $CDCl_3$) of **4** at 298 K.

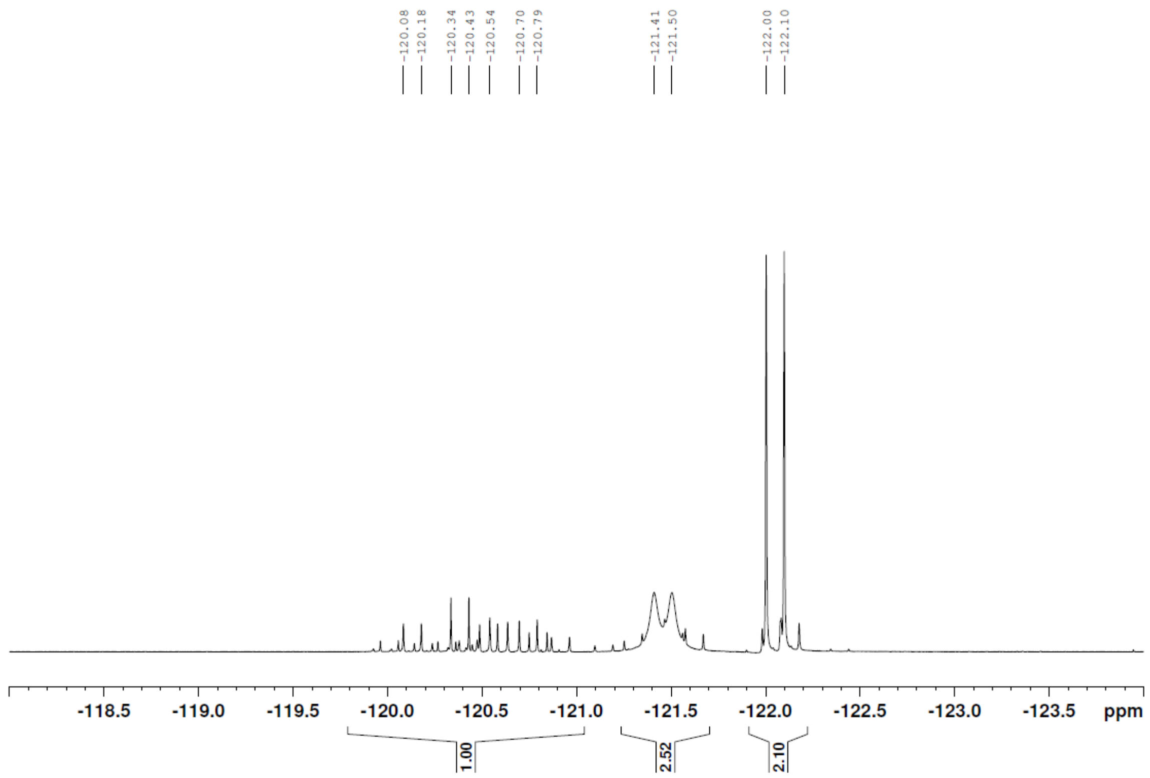

Figure 29. ^{19}F NMR spectrum (565 MHz, $CDCl_3$) of **4** at 298 K.

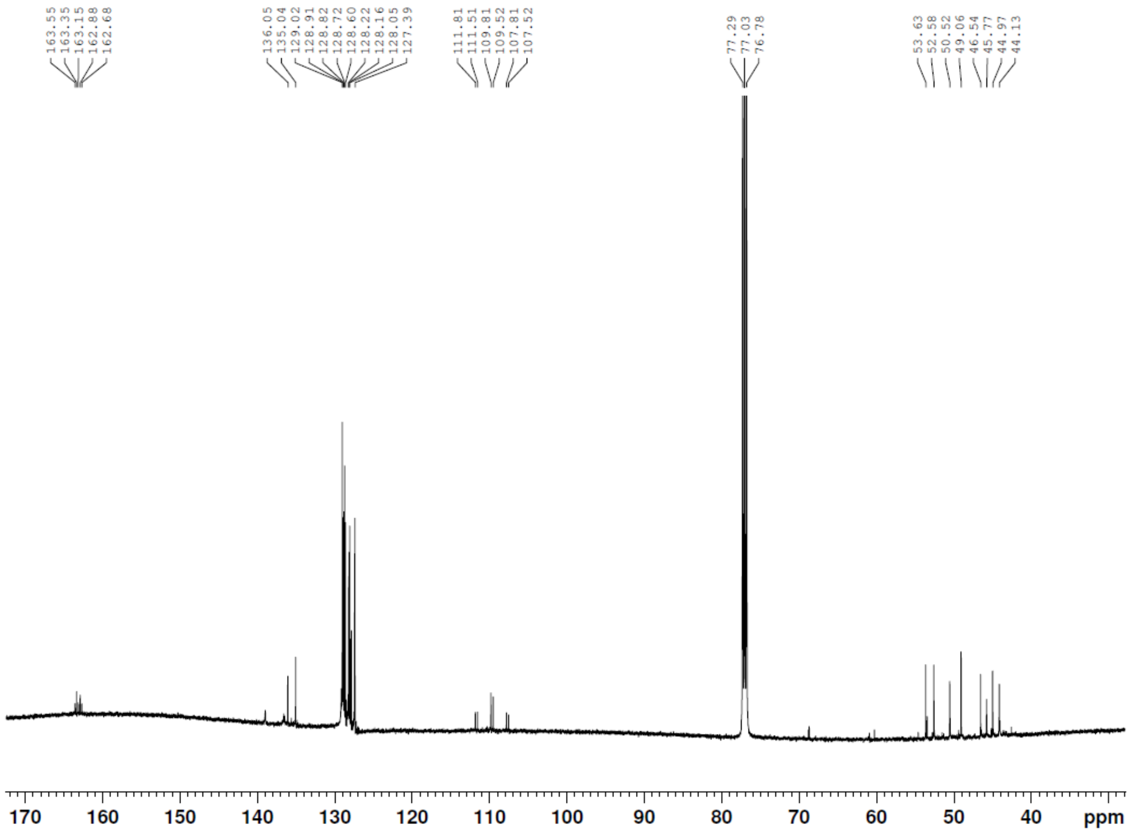

Figure 30. $^{13}C\{^{1}H\}$ NMR spectrum (151 MHz, $CDCl_3$) of **4** at 298 K.

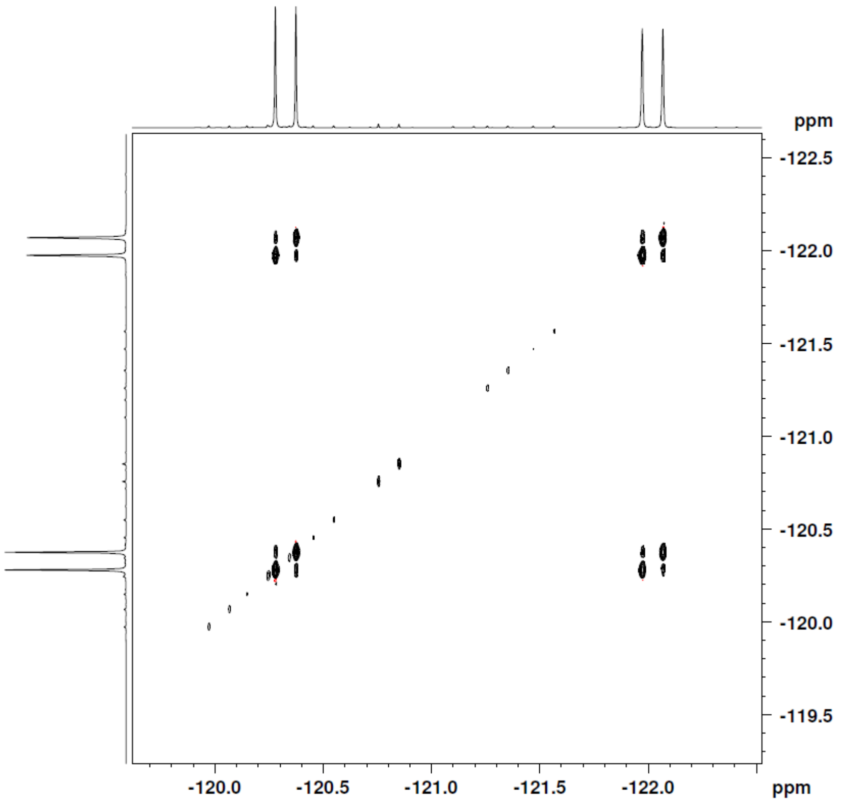

Figure 31. $^{19}F,^{19}F$-EXSY NMR spectrum (565 MHz, wet $CDCl_3$, mixing time 500 ms) of **4** at 298 K

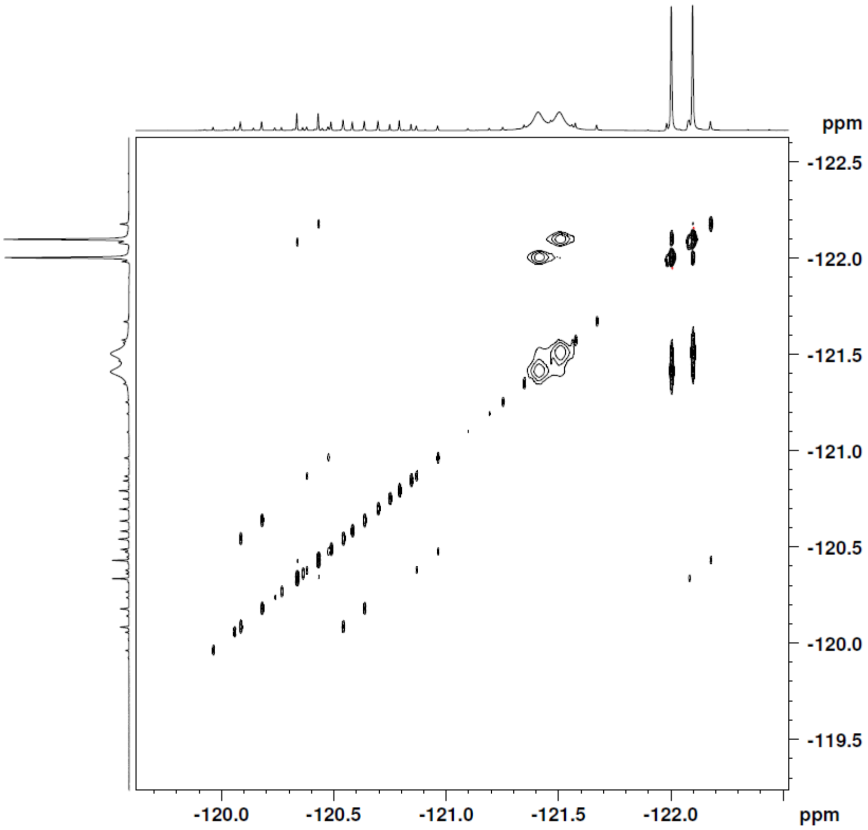

Figure 32. $^{19}F,^{19}F$-EXSY NMR spectrum (565 MHz, $CDCl_3$, mixing time 500 ms) of **4** at 298 K

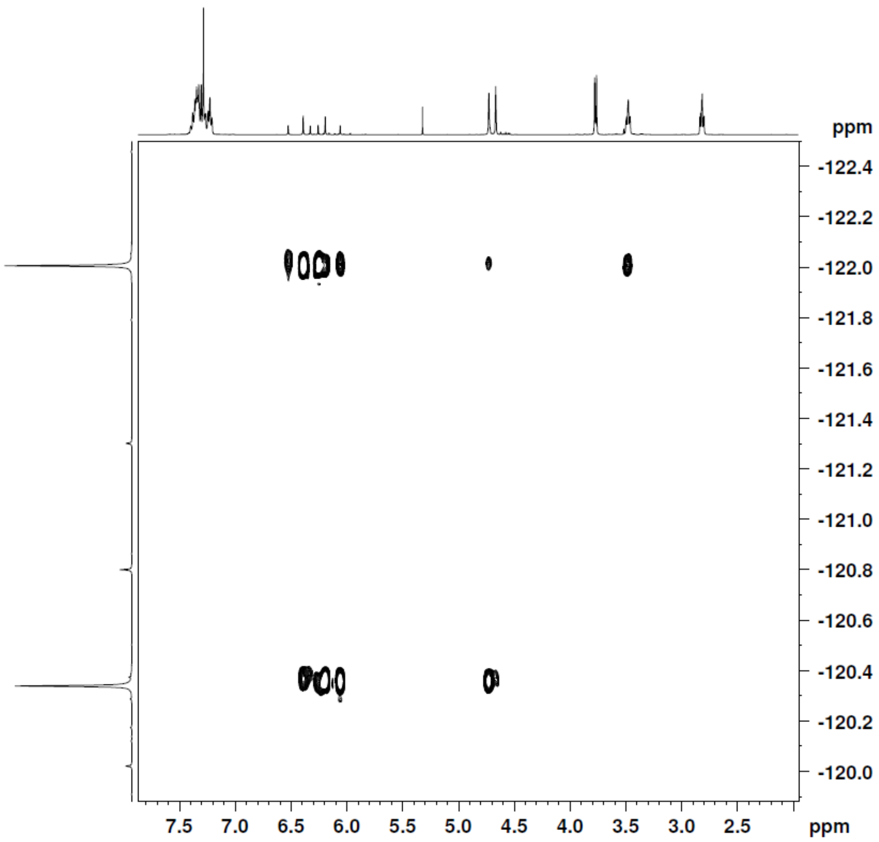

Figure 33. $^{1}H,^{19}F$-HOESY NMR spectrum (400 MHz, wet $CDCl_3$, mixing time 300 ms) of **4** at 298 K.

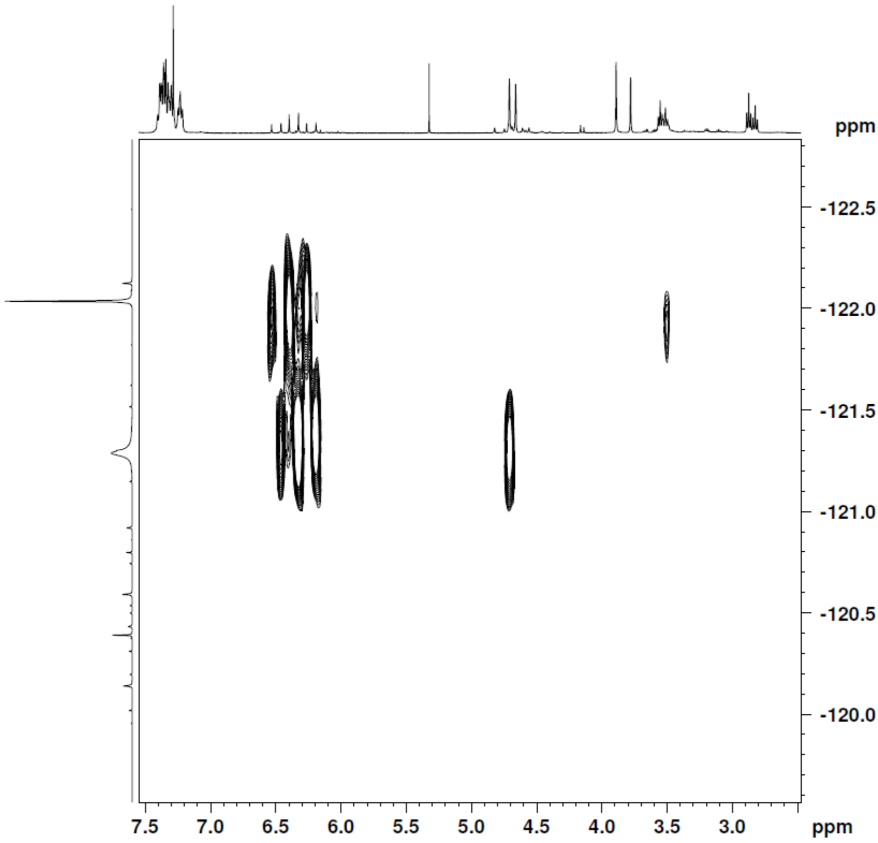

Figure 34. $^{1}H,^{19}F$-HOESY NMR spectrum (400 MHz, $CDCl_3$, mixing time 300 ms) of **4** at 298 K.

6. Solvent Effects

Compound **3** was investigated by NMR in a range of solvents. Higher population of *E,Z*-**3a** was observed in solvents with higher polarity (acetone or methanol). Dichloromethane has higher polarity than chloroform (ε = 8.93 vs. 4.71, respectively).

Table 1. Experimental populations of conformers of **3** in different solvents at 298 K.

**solvent**	***E,Z*-3a [%]**	***Z,Z*-3b [%]**	***E,E*-3c [%]**
$CDCl_3$	49	41	10
$CDCl_3$ + 1 eq. pyridine	48	42	9
80 % $CS_2/CDCl_3$	48	42	9
benzene-d_6	50	40	10
CD_2Cl_2 (78 mM)	54	36	10
CD_2Cl_2 (3 mM)	54	36	10
CD_3OD	59	31	10
acetone-d_6	61	27	12

The populations remained constant in $CDCl_3$ over the range of 278 – 318 K.

7. NMR Exchange Studies of Rotational Barriers

Rotational barriers of difluoroacetamide group of **4** in different solvents were estimated by a series of 1D EXSY spectra with different mixing time (100 – 500 ms) using initial rate approximation.

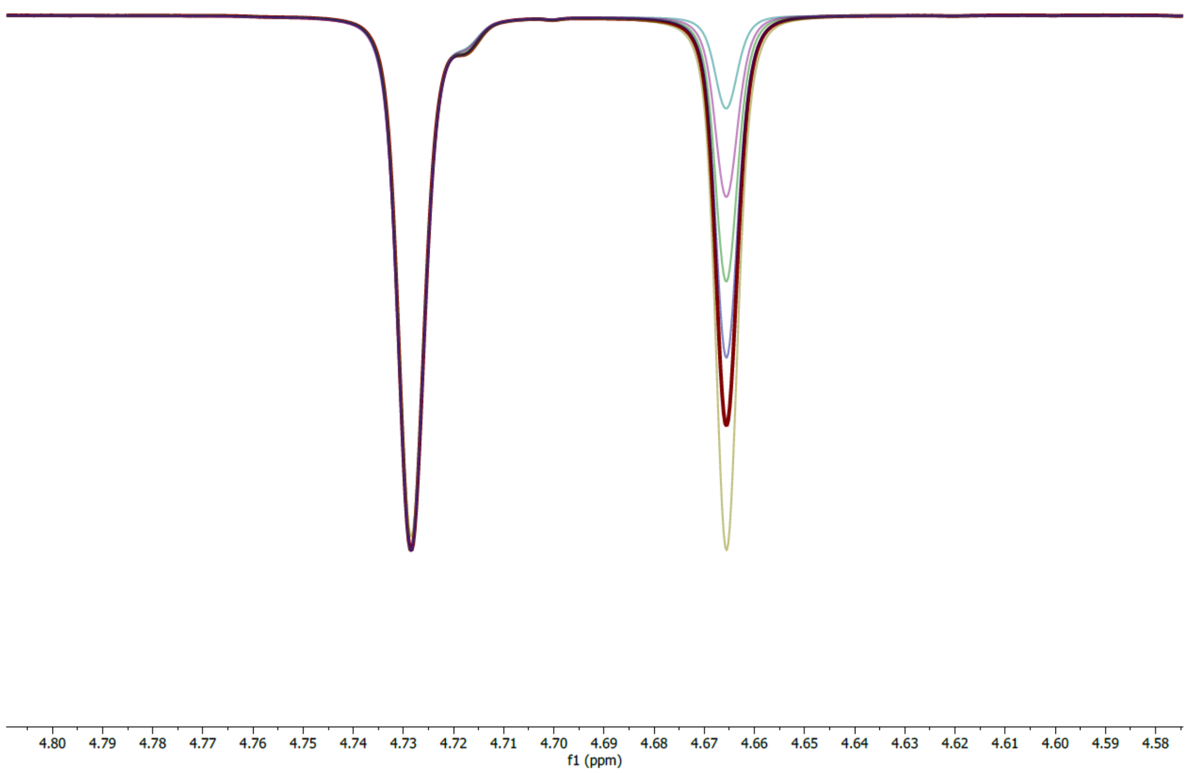

Figure 35. Stacked ^{1}H 1D NOESY NMR spectra (500 MHz, wet $CDCl_3$, mixing time 100 – 800 ms) of **4** in at 298 K used to determine the exchange rate. The peak at ~4.73 ppm (benzylic CH_2) was irradiated and the exchange peak at 4.66 ppm was monitored.

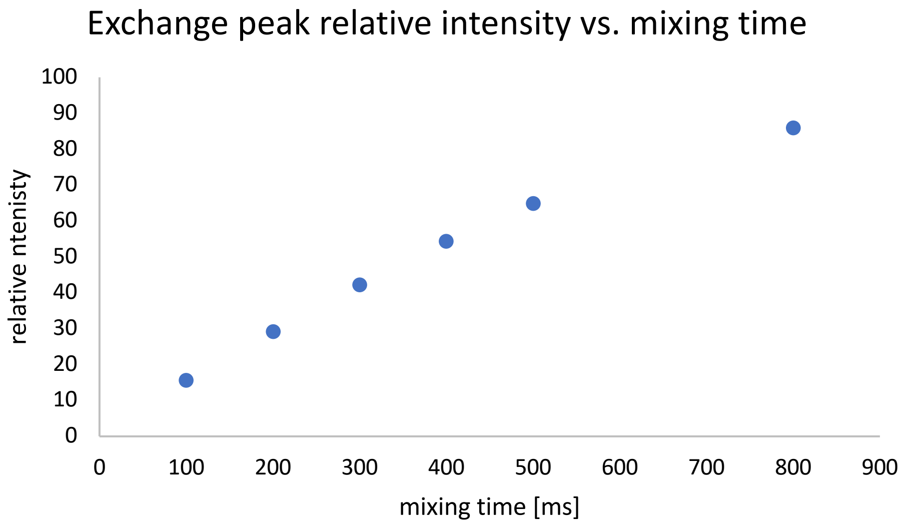

Figure 36. Plot of relative exchange peak intensity from ^{1}H 1D NOESY NMR spectra (500 MHz, wet $CDCl_3$, mixing time 100 – 800 ms) of **4** at 298 K. Initial rate approximation is valid up to 500 ms mixing time.

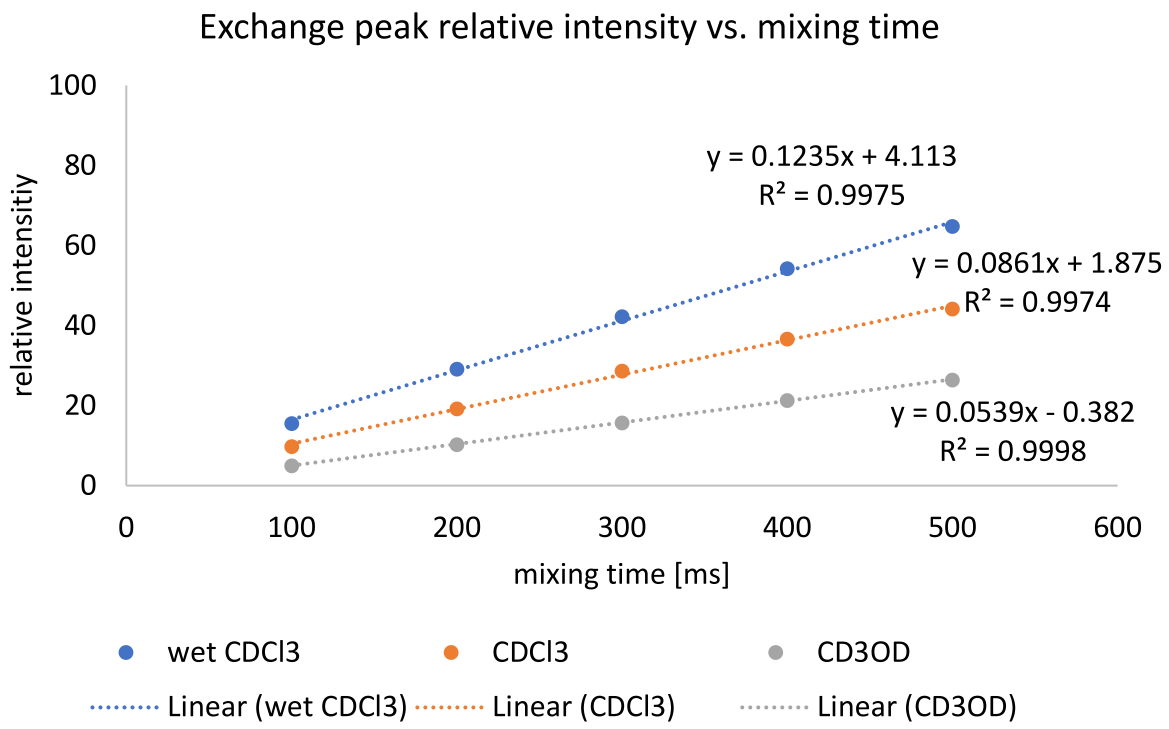

Figure 37. Plot of relative exchange peak intensities from ^{1}H 1D NOESY NMR spectra (500 MHz, $CDCl_3$, mixing time 100 – 500 ms) of **4** in different solvents at 298 K. Initial rate approximation was used to derive exchange rates as the slope of the line.

Table 2. Extracted exchange rates for the difluoroacetamide rotation in different solvents and the Gibbs energies of rotation based on Eyring equation.

solvent	rate [s^{-1}]	ΔG [kJ/mol]
wet $CDCl_3$	1.235	72.46
$CDCl_3$	0.861	73.35
CD_3OD	0.539	74.51
computed (in $CDCl_3$)	0.897	73.25

The rotational barriers of difluoroacetamdies are energetically higher (and the rotational slower) than for the corresponding (oligo)ureas which have rotational barriers in the range 49 – 59 kJ·mol^{-1} as determined by VT-NMR. This indicates a higher C=N double bond character in the difluoroacetamide group than in urea.

8. Urea Donor/Acceptor System

When a stronger H-bond donor such as arylurea **6** is present, it completely forces the preference of the amide to an H-bond acceptor (**6a**) in $CDCl_3$. However, a weak exchange crosspeak is visible in 2D NOESY spectrum where the urea is an H-bond acceptor (**6b**). The urea NH chemical shift changes from δ_H 7.83 (intramolecular H-bond) to 6.23 ppm (no intramolecular N-H-O bond) in $CDCl_3$. The computed chemical shift of the C-H does not change upon acting as the H-bond donor. Interestingly, the NBO analysis suggested that the orbital interactions of the H-bond are quite comparable (2.7 for urea donor vs. 0.9 kcal/mol for C-H donor) but certainly other factors including entropy influence the H-bond properties. However, in acetone-d_6, a conformer was detected, which, based on a HOE crosspeak, could be assigned as **6b**. Again, the change in solvent polarity can result in minor conformation to be populated (δ_{NH} change from 8.25 to 7.78 ppm, where urea binds acetone).

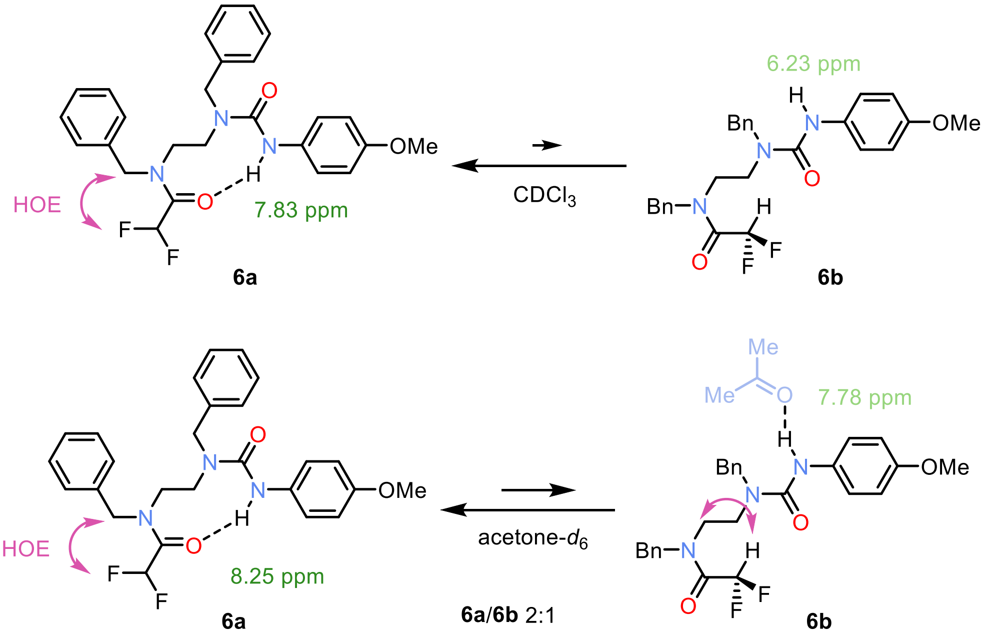

^{1}H NMR Assigment

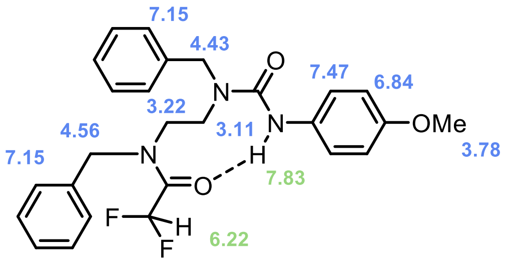

^{13}C NMR Assigment

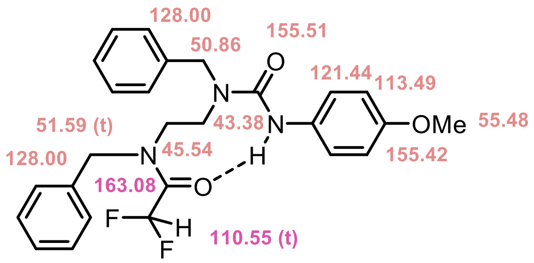

Figure 38. Assignment of ^{1}H and ^{13}C NMR shifts (500 MHz, $CDCl_3$ of **6** at 298 K using COSY, HSQC, HMBC and 2D NOESY spectra.

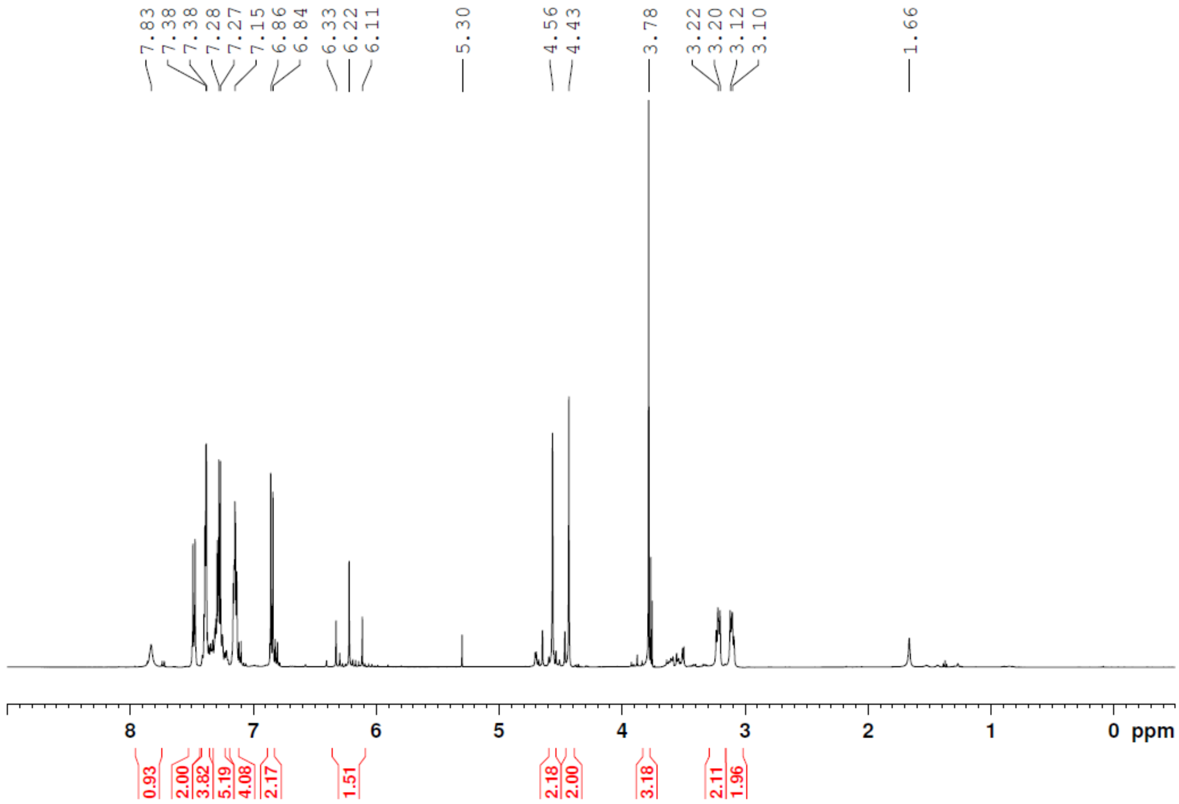

Figure 39. ^{1}H NMR spectrum (500 MHz, $CDCl_3$) of **6** at 298 K.

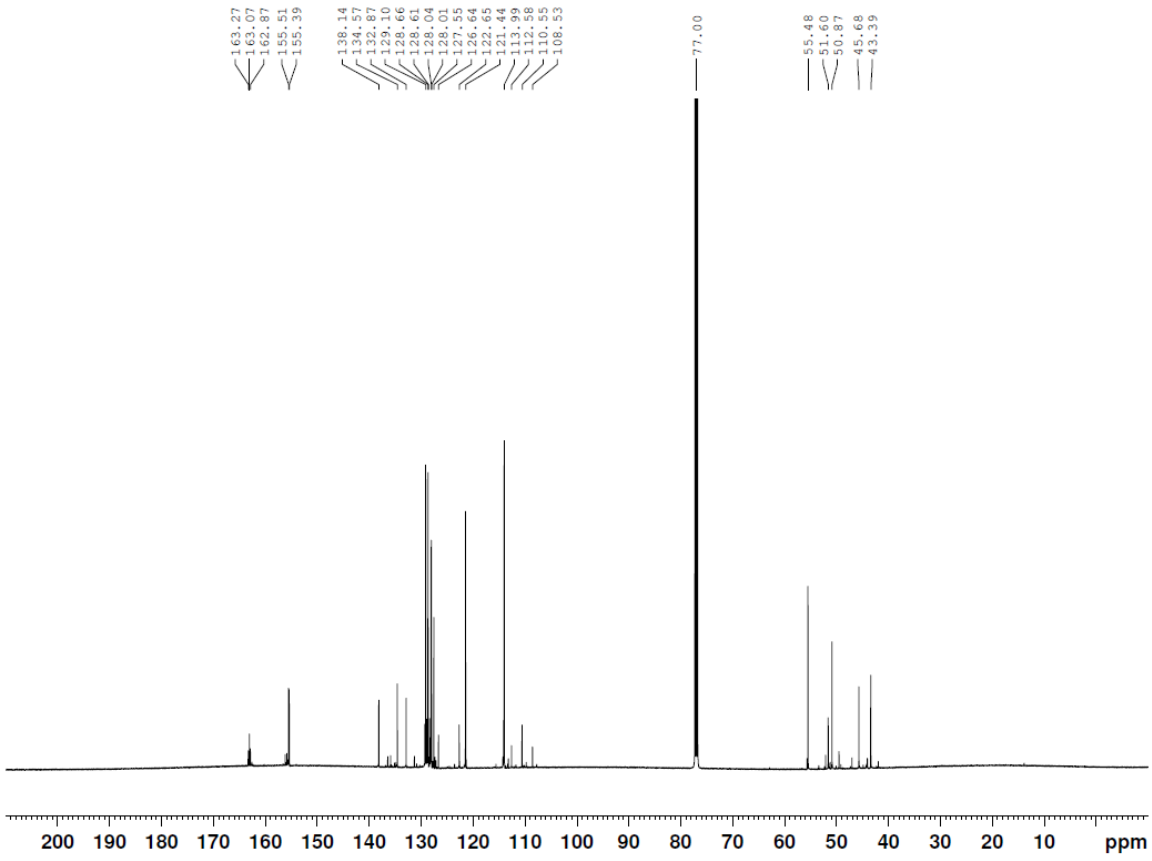

Figure 40. ^{13}C{^{1}H} NMR spectrum (126 MHz, $CDCl_3$) of **6** at 298 K.

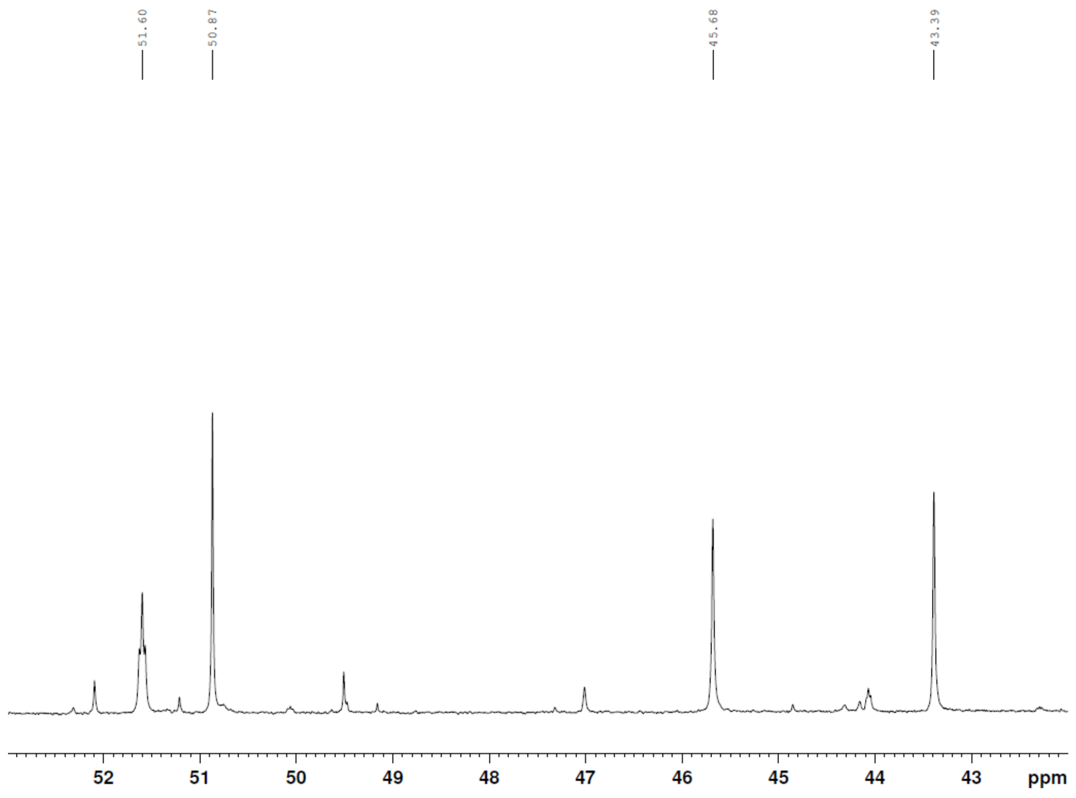

Figure 41. $^{13}C\{^{1}H\}$ NMR spectrum section (126 MHz, $CDCl_3$) of **6** at 298 K showing a triplet at 51.60 ppm.

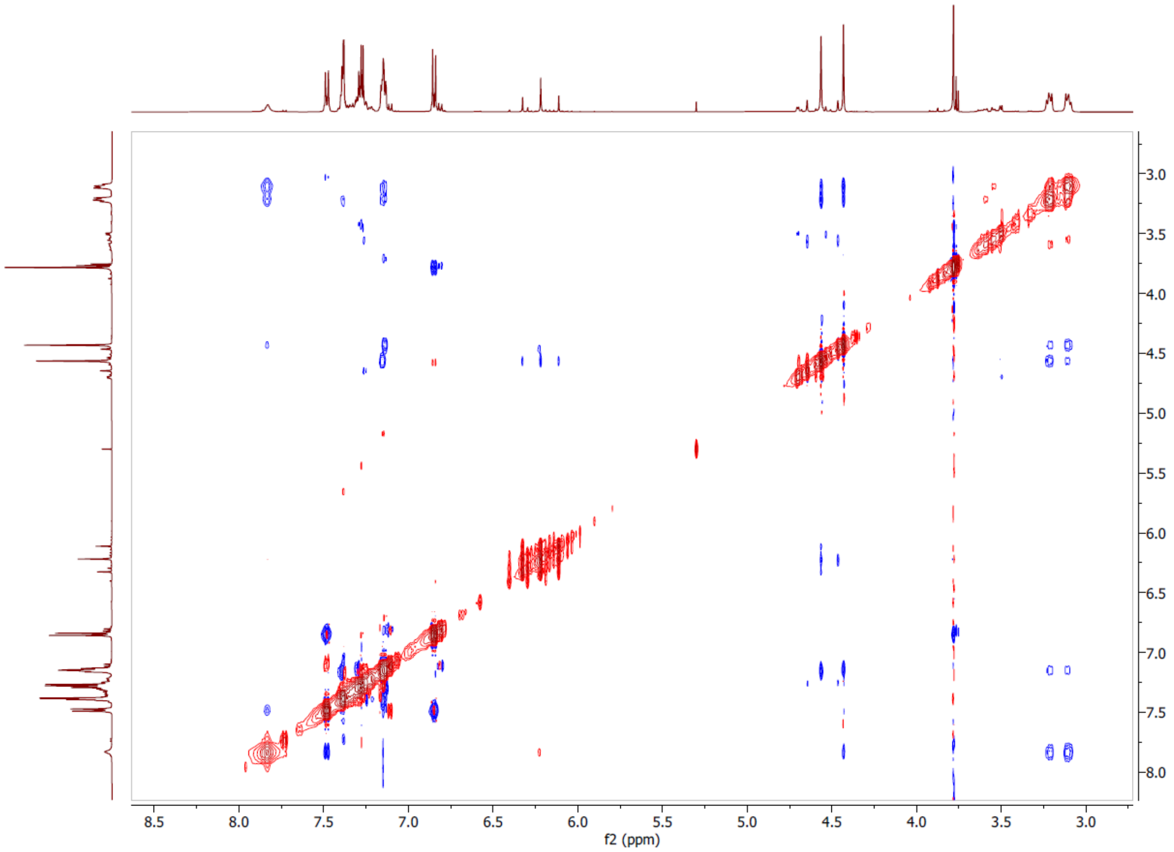

Figure 42. $^{1}H,^{1}H$ 2D NOESY NMR spectrum (500 MHz, $CDCl_3$, 500 ms mixing time) of **6** at 298 K showing NOE (blue) and exchange (red) correlation peaks.

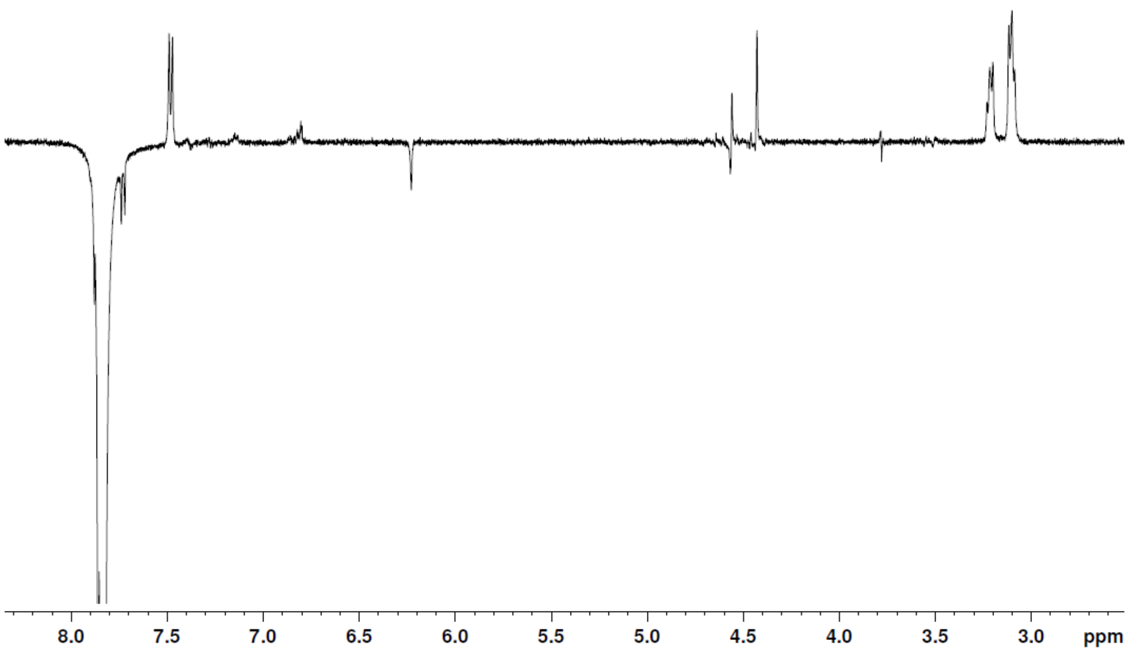

Figure 43. ^{1}H 1D NOESY NMR spectrum (500 MHz, $CDCl_3$, 500 ms mixing time) of **6** at 298 K upon irradiation of the urea NH proton (7.83 ppm) showing an exchange with minor conformer NH (6.23 ppm) and NOE correlations to the ethylene bridge, 4-methoxyphenyl and benzylic protons.

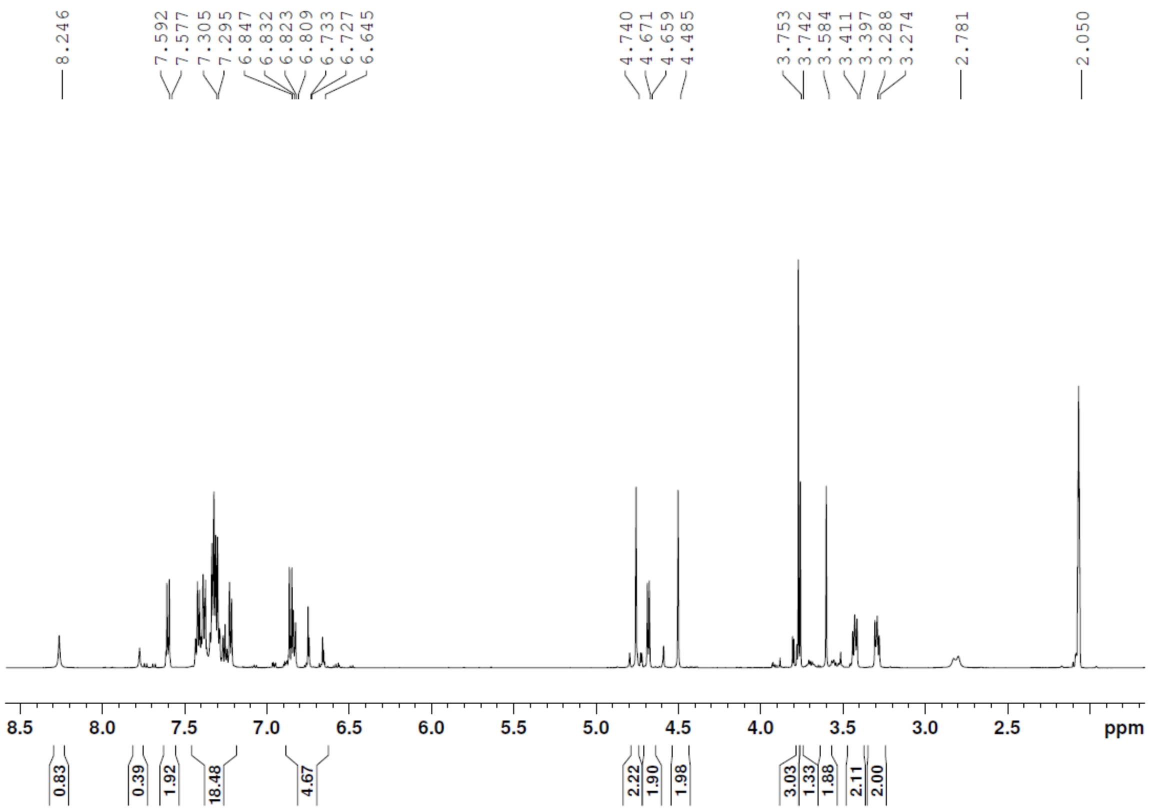

Figure 44. ^{1}H NMR spectrum (500 MHz, acetone-d_6) of **6** at 298 K.

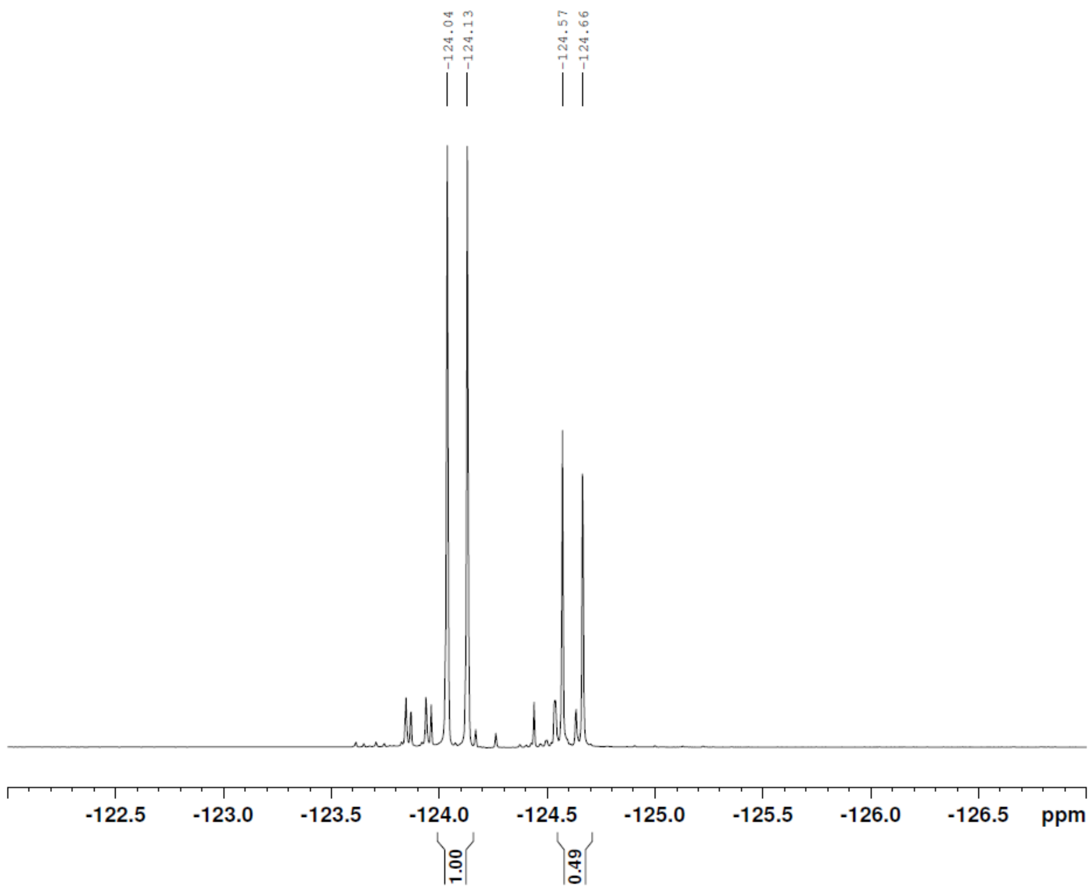

Figure 45. ^{19}F NMR spectrum (576 MHz, acetone-d_6) of **6** at 298 K.

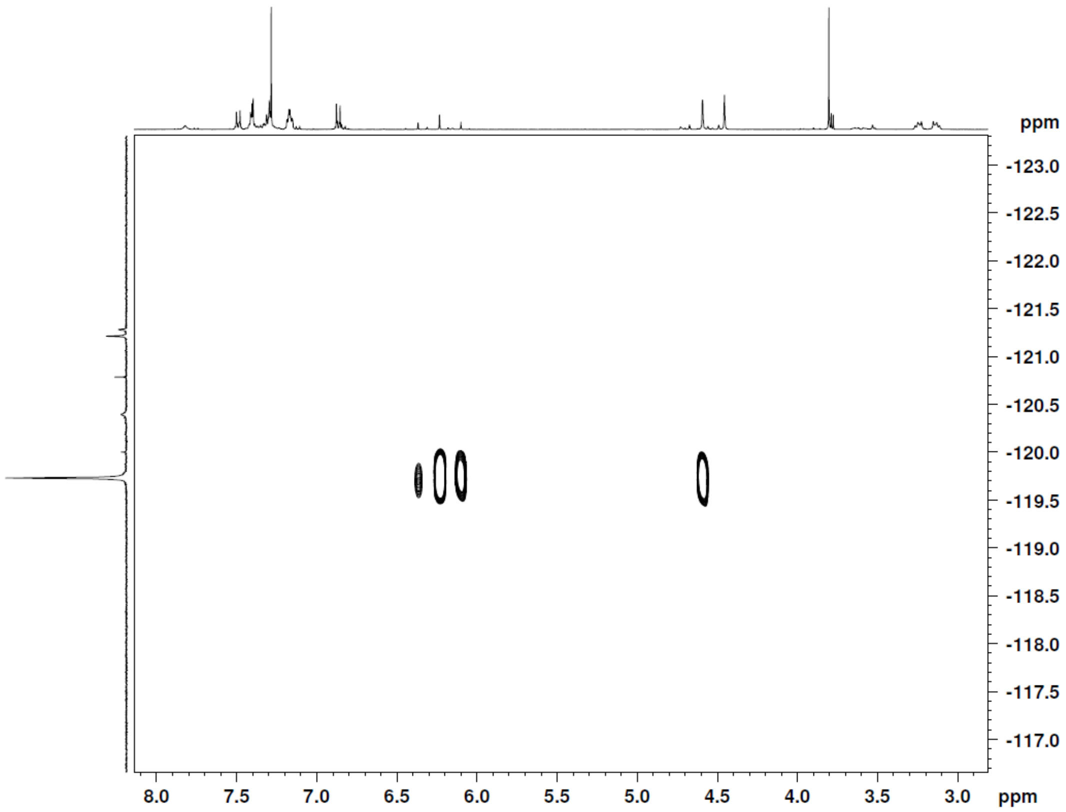

Figure 46. $^{1}H,^{19}F$-HOESY NMR spectrum (400 MHz, $CDCl_3$, mixing time 300 ms) of **6** at 298 K, showing only a major conformer **6a** and crosspeak to a benzylic position (4.56 ppm).

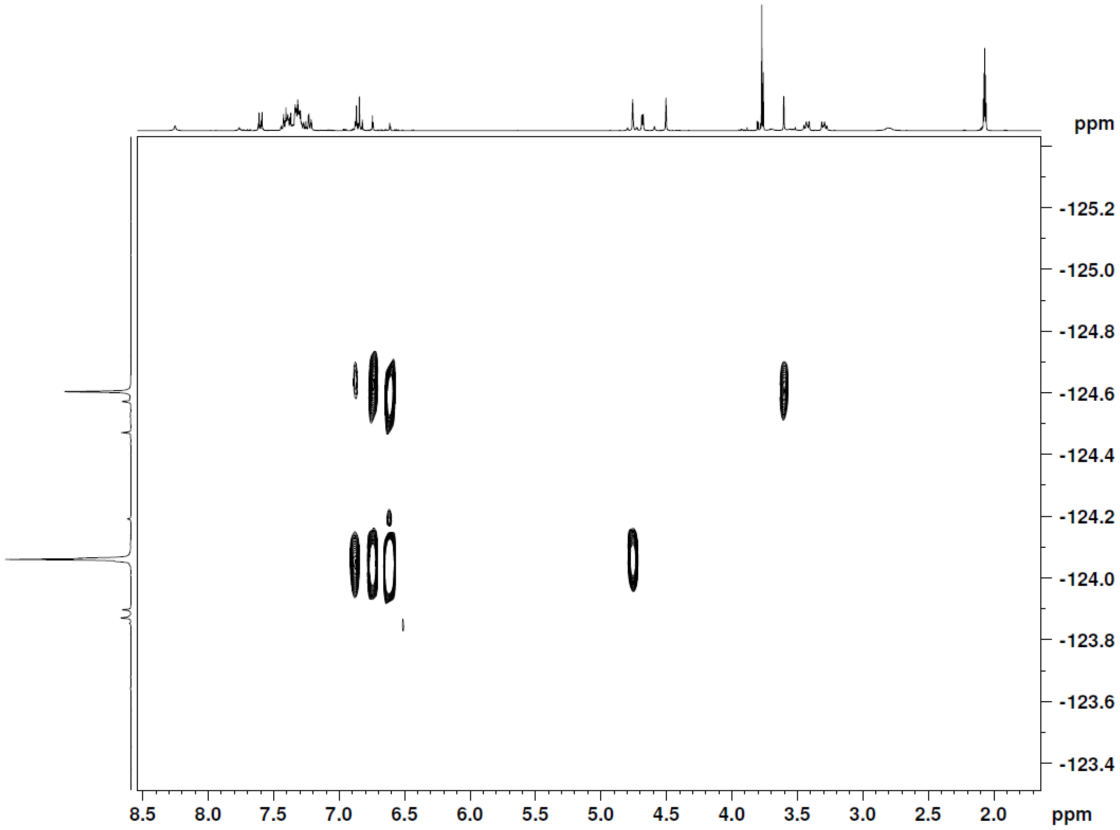

Figure 47. $^{1}H,^{19}F$-HOESY NMR spectrum (400 MHz, acetone-d_6, mixing time 300 ms) of **6** at 298 K, showing a major conformer **6a** as well as a minor conformation **6b** (crosspeak to ethylene bridge).

9. Crystallography

X-ray diffraction experiments on **3** were carried out at 100(2) K on a Bruker D8 Venture using Mo-K_α radiation (λ = 0.71073 Å) radiation. Intensities were integrated in SAINT[1] and absorption corrections based on equivalent reflections were applied using SADABS.[2] The structure was solved using ShelXT[3] all refined by full matrix least squares against F^2 in ShelXL[4,5] using Olex2[6]. All of the non-hydrogen atoms were refined anisotropically. While all of the hydrogen atoms were located geometrically and refined using a riding model. Crystal structure and refinement data are given in Table 1. Crystallographic data have been deposited with the Cambridge Crystallographic Data Centre as supplementary publication CCDC 2264158. Copies of the data can be obtained free of charge on application to CCDC, 12 Union Road, Cambridge CB2 1EZ, UK [fax(+44) 1223 336033, e-mail: deposit@ccdc.cam.ac.uk].

1. Bruker, SAINT+ v8.38A Integration Engine, Data Reduction Software, Bruker Analytical X-ray Instruments Inc., Madison, WI, USA 2015.
2. Bruker, SADABS 2014/5, Bruker AXS area detector scaling and absorption correction, Bruker Analytical X-ray Instruments Inc., Madison, Wisconsin, USA 2014/5.
3. Sheldrick, G. M., SHELXT - Integrated space-group and crystal-structure determination. Acta Crystallographica a-Foundation and Advances 2015, 71, 3-8.
4. Sheldrick, G. M., A short history of SHELX. Acta Crystallographica Section A 2008, 64, 112-122.
5. Sheldrick, G. M., Crystal structure refinement with SHELXL. Acta Crystallographica Section C-Structural Chemistry 2015, 71, 3-8.
6. Dolomanov, O. V.; Bourhis, L. J.; Gildea, R. J.; Howard, J. A. K.; Puschmann, H., OLEX2: a complete structure solution, refinement and analysis program. Journal of Applied Crystallography 2009, 42, 339-341.

Table 3. Crystal data and structure refinement for **3.**

Identification code	**3**
Empirical formula	$C_{22}H_{22}Cl_6F_4N_2O_2$
Formula weight	635.11
Temperature/K	100(2)
Crystal system	triclinic
Space group	*P*-1
a/Å	5.7583(2)
b/Å	10.0636(4)
c/Å	12.6561(5)
α/°	66.9880(10)
β/°	86.9530(10)
γ/°	87.6390(10)
Volume/Å^3	673.93(4)
Z	1
ρ_{calc}g/cm^3	1.565
μ/mm^{-1}	0.689
F(000)	322.0
Crystal size/mm^3	0.344 × 0.323 × 0.268
Radiation	MoKα (λ = 0.71073)
2Θ range for data collection/°	3.5 to 55.904
Index ranges	$-7 \le h \le 7$, $-13 \le k \le 13$, $-16 \le l \le 16$
Reflections collected	38662
Independent reflections	3236 [R_{int} = 0.0256, R_{sigma} = 0.0143]
Data/restraints/parameters	3236/0/163
Goodness-of-fit on F^2	1.032
Final R indexes [I>=2σ (I)]	R_1 = 0.0300, wR_2 = 0.0676
Final R indexes [all data]	R_1 = 0.0330, wR_2 = 0.0693
Largest diff. peak/hole / e Å^{-3}	0.51/-0.54

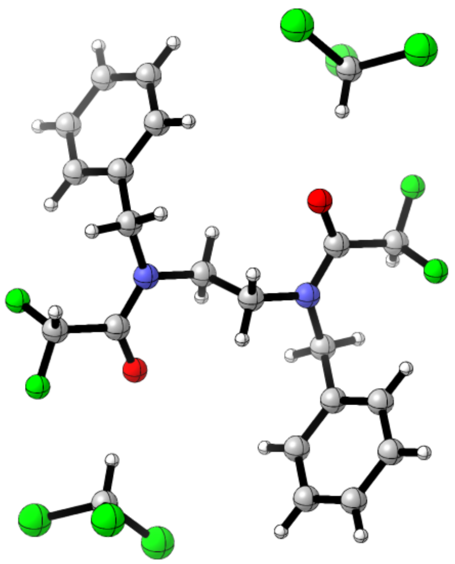

Figure 48. X-ray crystal structure of **3** as conformer **3b'**·$(CHCl_3)_2$.

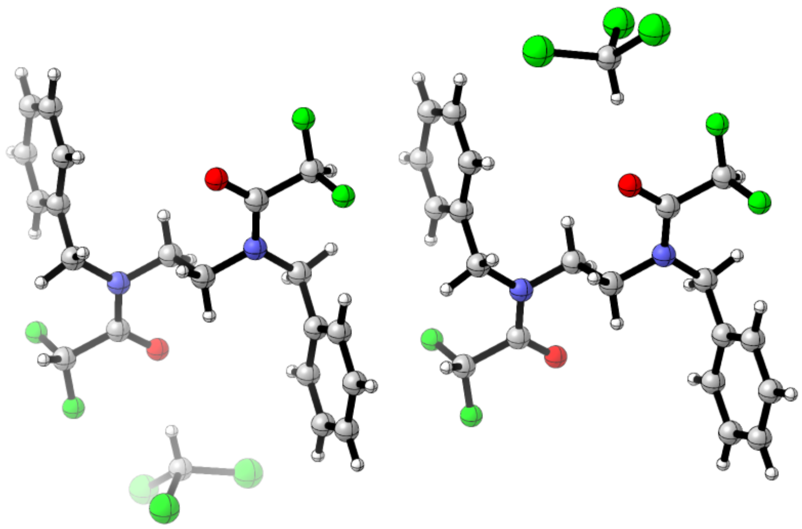

Figure 49. X-ray crystal structure of dimer (**3b'**·$CHCl_3)_2$, revealing CF_2H-phenyl noncovalent interactions.

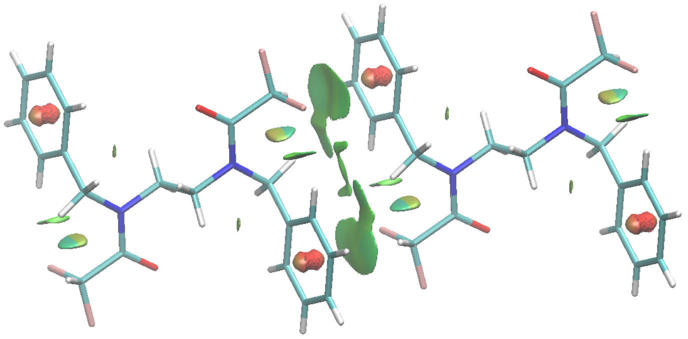

Figure 50. NCI Plot[5,6] of X-ray crystal structure of dimer (**3b'**)$_2$, revealing weak CF_2H-phenyl intermolecular noncovalent interactions (green areas).

10.Computational Details

Geometry Optimization

Density functional theory (DFT) calculations were performed with ORCA 5.0.2.[7] The geometries were optimized using the hybrid B3LYP functional[8,9] with D3(BJ) dispersion correction[10] and the triple-zeta plus polarization def2-TZVP basis set.[11] All DFT calculations were conducted with the *Defgrid2* integration grid, TightSCF convergence criteria, and Rijcosx approximation. Frequency calculations were performed at the same level of theory as for geometry optimizations to verify the stationary points as minima (no imaginary frequencies); or transition states (one imaginary frequency) as well as to obtain thermal Gibbs free energy corrections at 298 K. Grimme's entropy corrections using quasi-rigid rotor harmonic approximation (qRRHO)[12] were applied to all frequencies below 100 cm^{-1}.

Conformational screen for **3** was performed by GFN2-XTB metadynamics.[13]

Single-Point Calculations

To refine the computed energy, single point calculations were performed in ORCA 5.0.2 at B3LYP-D3(BJ)/def2-TZVP/SMD(CH_2Cl_2) level of theory. SMD implicit solvation model for chloroform was used.[14]

DLPNO-CCSD(T1) Calculations

To refine the computed energy, single point calculations were performed in ORCA 5.0.2 at DLPNO-CCSD(T1)/def2-QZVPP/TightPNO level of theory using Rijcosx approximation.[15,16] VeryTightSCF convergence criteria were used. The T1 diagnostics value was <0.02 in all cases.

Gibbs Free Energies

The ΔG value was obtained by adding the corresponding free energy corrections at 298 K and SMD(chloroform) solvation correction (sum of CPCM dielectric and G_{CDS} contributions) calculated at the B3LYP-D3(BJ)/def2-TZVP level, to ΔE, calculated at the single-point calculation at DLPNO-CCSD(T1) level of theory.

NMR Calculations

NMR scalar coupling constants were computed in ORCA 5.0.2 at DSD-PBEP86[17,18]/pcSseg-3[19] level of theory using AutoAux option, VeryTightSCF settings, and SMD solvation model for chloroform.

Computed Energies

Table 4. Computed Gibbs free energies (chloroform, 298 K, 1 M) of **5** and its rotational TS based on DLPNO-CCSD(T1)/def2-QZVPP/TightPNO single point calculations.

**Structure**	**B3LYP/TZVP**	G_{corr}	SMD	CCSD(T1)	ΔG_{corr}	**Δ*G*solv [kJ·mol⁻¹]**
**5**_SM	-486.289627	0.081713	-0.01083	-485.769619	-486.218742	0.00
**5**_TS1	-486.258562	0.081680	-0.01033	-485.742191	-486.187207	73.25
**5**_TS2	-486.254016	0.081241	-0.01092	-485.736550	-486.183690	85.35
**5**_SM_F_syn	-486.285227	0.081591	-0.01565	-485.765512	-485.699571	-2.20
**5**_TS_F_syn	-486.257714	0.081718	-0.01219	-485.741486	-485.671958	70.30

The rotational barrier between structures **5**_SM and **5**_TS1 is 73.25 kJ·mol^{-1}. The methyl groups are puckered towards the carbonyl oxygen in the transition state. The structure **5**_TS2 has a different *N*-pyramidalization with methyl groups puckered towards the fluorine atoms and is higher in energy. Nearly identical reaction barrier was computed from the conformer with fluorine atom *synclinal* to the C=O bond (72.50 kJ·mol^{-1}). The structures are depicted in the chapter Coordinates of Optimized Structures.

Table 5. Computed Gibbs free energies (chloroform, 298 K, 1 M) of conformations of **3** based on DLPNO-CCSD(T1)/def2-TZVPP/TightPNO single point calculations.

Structure	B3LYP/TZVP	G_{corr}	SMD	CCSD(T1)	ΔG_{corr}	ΔGsolv [kJ·mol^{-1}]	population [%]
conf1 (***E*,*Z***)	-971.383515	0.168813	-0.024671	-970.071826	-969.927685	6.72	1.52
conf1b	-971.379001	0.168581	-0.029410	-970.067431	-969.928261	5.21	2.79
conf1c	-971.378934	0.168592	-0.029663	-970.067453	-969.928524	4.52	3.69
conf23	-971.388572	0.170387	-0.020784	-970.076803	-969.927200	7.99	0.91
conf23b	-971.379271	0.169643	-0.031940	-970.067948	-969.930245	**0.00**	22.85
conf23c	-971.378699	0.170013	-0.031284	-970.067224	-969.928494	4.60	3.57
conf44 (***E*,*E***)	-971.381901	0.169642	-0.027020	-970.070378	-969.927756	6.53	1.64
conf44b	-971.386030	0.169578	-0.022655	-970.074308	-969.927384	7.51	1.10
conf44c	-971.375834	0.169489	-0.033294	-970.064531	-969.928336	5.01	3.02
conf68	-971.383085	0.168835	-0.025388	-970.071503	-969.928056	5.75	2.25
conf68b	-971.387341	0.168881	-0.020626	-970.075606	-969.927351	7.60	1.06
conf68c	-971.378957	0.168825	-0.030009	-970.067606	-969.928790	3.82	4.89
conf68d	-971.376635	0.168322	-0.032058	-970.065044	-969.928779	3.85	4.84
conf76	-971.384228	0.169180	-0.022126	-970.072552	-969.925499	12.46	0.15
conf164(***Z*,*Z***)	-971.386983	0.169241	-0.022123	-970.075459	-969.928341	5.00	3.04
conf164b	-971.389818	0.169384	-0.018875	-970.078204	-969.927694	6.70	1.53
conf164c	-971.379715	0.169455	-0.029458	-970.068634	-969.928636	4.22	4.15
conf164d	-971.377611	0.169091	-0.032554	-970.066605	-969.930068	0.46	18.95
conf164e	-971.380872	0.169739	-0.025774	-970.070050	-969.926084	10.92	0.28
conf_Xray	-971.380225	0.168241	-0.028104	-970.068994	-969.928857	3.64	5.25
conf250	-971.378978	0.168794	-0.030172	-970.067575	-969.928953	3.39	5.81
conf250b	-971.387361	0.168857	-0.020639	-970.075609	-969.927390	7.49	1.11
conf250c	-971.376659	0.168306	-0.032166	-970.065061	-969.928921	3.48	5.62

Total calculated populations of NMR conformers: ***E*,*Z*-3a** (35.3 %); ***Z*,*Z*-3b** (39.0 %) and ***E*,*E*-3c** (25.7 %).

Experimental NMR ratios in $CDCl_3$: ***E*,*Z*-3a** (49 %), ***Z*,*Z*-3b** (41 %); ***E*,*E*-3c** (10 %). The computed ratios match the experimental values.

The X-ray structure (conformer **3b'**) is not the most populated structure in solution and accounts for ~5 % of the population. The X-ray structure contains *antiperiplanar* N-CH_2CH_2-N, while the more populated conformers of **3b** in solution have *gauche* arrangement.

Table 6. Computed Gibbs free energies (chloroform, 298 K, 1 M) conformations of **3** based on B3LYP-D3(BJ)/def2-TZVP/SMD single point calculations.

Structure	B3LYP/TZVP	G_{corr}	SMD	ΔG_{corr}	ΔGsolv [kJ·mol^{-1}]	population [%]
conf1	-971.383515	0.168813	-0.024671	-969.927685	5.76	2.13
conf1b	-971.379001	0.168581	-0.029410	-969.928261	4.56	3.46
conf1c	-971.378934	0.168592	-0.029663	-969.928524	4.10	4.16
conf23	-971.388572	0.170387	-0.020784	-969.927200	6.83	1.39
conf23b	-971.379271	0.169643	-0.031940	-969.930245	**0.00**	21.81
conf23c	-971.378699	0.170013	-0.031284	-969.928494	4.20	4.01
conf44	-971.381901	0.169642	-0.027020	-969.927756	6.01	1.93
conf44b	-971.386030	0.169578	-0.022655	-969.927384	6.46	1.61
conf44c	-971.375834	0.169489	-0.033294	-969.928336	5.07	2.82
conf68	-971.383085	0.168835	-0.025388	-969.928056	5.07	2.82
conf68b	-971.387341	0.168881	-0.020626	-969.927351	6.52	1.57
conf68c	-971.378957	0.168825	-0.030009	-969.928790	3.75	4.80
conf68d	-971.376635	0.168322	-0.032058	-969.928779	3.14	6.13
conf76	-971.384228	0.169180	-0.022126	-969.925499	11.54	0.21
conf164	-971.386983	0.169241	-0.022123	-969.928341	4.47	3.59
conf164b	-971.389818	0.169384	-0.018875	-969.927694	5.93	1.99
conf164c	-971.379715	0.169455	-0.029458	-969.928636	4.86	3.07
conf164d	-971.377611	0.169091	-0.032554	-969.930068	1.30	12.93
conf164e	-971.380872	0.169739	-0.025774	-969.926084	12.24	0.16
conf_Xray	-971.380225	0.168241	-0.028104	-971.240088	3.89	4.54
conf250	-971.378978	0.168794	-0.030172	-969.928953	3.18	6.04
conf250b	-971.387361	0.168857	-0.020639	-969.927390	6.37	1.67
conf250c	-971.376659	0.168306	-0.032166	-969.928921	2.76	7.17

Total calculated populations of NMR conformers: ***E,Z*-3a** (37.0 %); ***Z,Z*-3b** (32.6 %) and ***E,E*-3c** (30.4 %). Experimental NMR ratios in $CDCl_3$: ***E,Z*-3a** (49 %), ***Z,Z*-3b** (41 %); ***E,E*-3c** (10 %). The computed ratios by B3LYP single-point energies are close to the DLPNO-CCSD(T1) values.

Table 7. Computed Gibbs free energies (chloroform, 298 K, 1 M) conformations of full model **3_Ph** which includes benzyl groups based on B3LYP-D3(BJ)/def2-TZVP/SMD single point calculations. These calculations confirm that the crystal structure is not the most populated conformation in solution.

Structure	B3LYP/TZVP	G_{corr}	SMD	ΔG_{corr}	ΔGsolv [kJ·mol^{-1}]
conf23b_Ph	-1433.405560	0.322610	-0.044775	-1433.127726	3.22
conf164d_Ph	-1433.404428	0.321275	-0.045800	-1433.128953	0.00
conf_crystal_Ph	-1433.407288	0.322457	-0.039659	-1433.124490	11.72

Computed Dipole Moments

Table 8. Computed dipole moments in Debye of selected *N,N'*-dimethyl conformers in the gas phase at B3LYP-D3(BJ)/def2-TZVP; in SMD(chloroform) model as well as compared to *N,N'*-dibenzyl derivatives.

Structure	dipole moment in the gas phase	dipole moment in the SMD model	dipole moment in the gas phase after the inclusion of phenyls
conf1c	8.56	10.82	na
conf23b	6.48	8.48	7.03
conf164d	7.87	10.31	8.38
conf_Xray	0.85	1.02	0.01
conf250c	2.36	3.11	na

Computed NMR *J*-Coupling

Conformer 1c

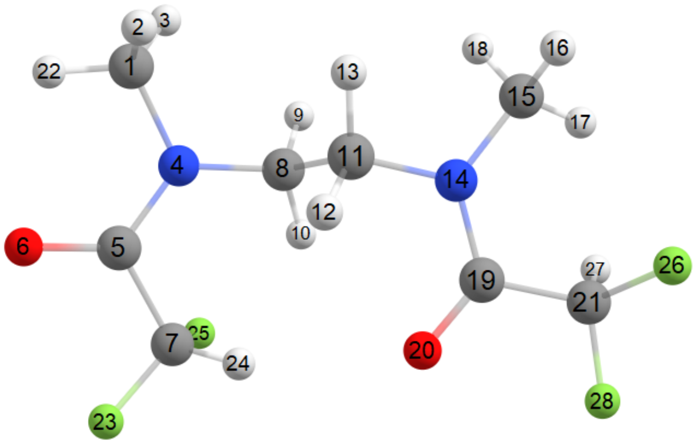

Table 9. Selected computed NMR coupling constants of conformer **1c**.

	1 C	5 C	7 C	8 C	24 H
**23 F**	1.897	32.828	-266.732	-0.17	61.252
**24 H**			222.403		
**25 F**	-1.13	25.296	-257.393	5.373	58.168
	**11 C**	**15 C**	**19C**	**21 C**	**27 H**
**26 F**	-1.382	4.024	27.538	-261.278	60.305
**27 H**			216.808		
**28 F**	1.77	-0.159	32.176	-269.116	61.885

Conformer 23b

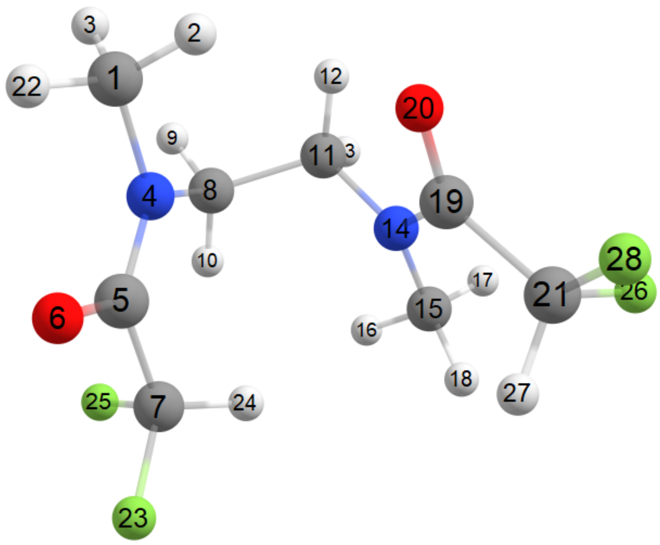

Table 10. Selected computed NMR coupling constants of conformer **23b**.

	1 C	5 C	7 C	8 C	
**23 F**	1.851	31.351	-266.156	-0.274	62.303
**24 H**			215.888		
**25 F**	-1.082	24.704	-261.582	4.774	60.086
	**11 C**	**15 C**	**19C**	**21 C**	**27 H**
**26 F**	-1.122	5.46	27.927	-259.695	60.065
**27 H**			217.086		
**28 F**	1.721	-0.226	31.767	-270.236	61.334

Conformer 164d

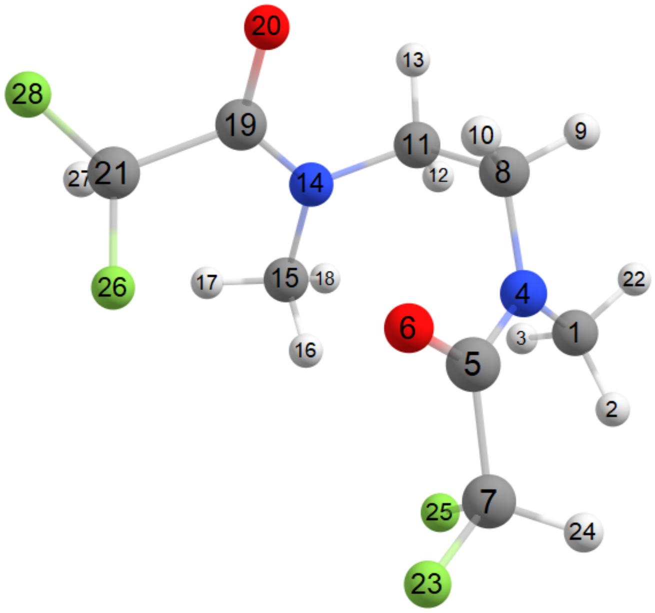

Table 11. Selected computed NMR coupling constants of conformer **164d**.

	1 C	5 C	7 C	8 C	24 H
**23 F**	-0.058	31.322	-268.912	1.719	61.926
**24 H**			217.019		
**25 F**	4.027	27.164	-257.248	-0.767	59.697
	**11 C**	**15 C**	**19C**	**21 C**	**27 H**
**26 F**	-0.734	4.358	27.691	-265.696	60.48
**27 H**			214.742		
**28 F**	1.86	-0.148	31.989	-266.595	62.053

Conformer 250c

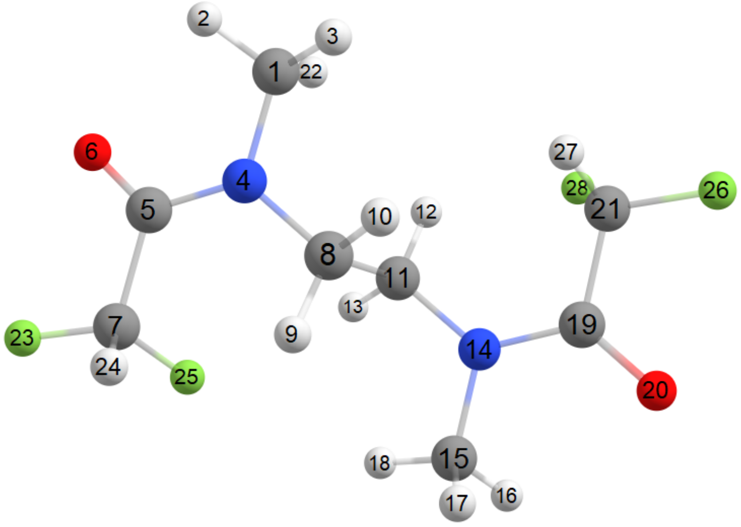

Table 12. Selected computed NMR coupling constants of conformer **250c**.

	1 C	5 C	7 C	8 C	24 H
**23 F**	1.938	31.762	-269.893	-0.134	61.898
**24 H**			217.143		
**25 F**	-1.091	27.378	-258.161	1.464	59.984
	**11 C**	**15 C**	**19C**	**21 C**	**27 H**
**26 F**	-0.264	1.873	32.064	-269	61.824
**27 H**			215.942		
**28 F**	5.578	-0.921	24.803	-262.025	59.889

Coordinates of Optimized Structures

Compound 3

Method: B3LYP-D3(BJ)/def2-TZVP

conf1

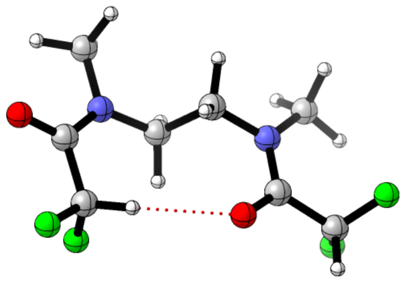

C	-4.477169000000	1.552424000000	-0.226753000000
H	-4.389629000000	2.157724000000	0.680181000000
H	-4.419566000000	2.209492000000	-1.096329000000
N	-3.424815000000	0.549903000000	-0.309780000000
C	-3.721096000000	-0.730955000000	0.057102000000
O	-4.813390000000	-1.085717000000	0.446566000000
C	-2.568111000000	-1.759205000000	-0.064286000000
C	-2.087248000000	1.016286000000	-0.627806000000
H	-2.160654000000	1.736407000000	-1.446512000000
H	-1.479233000000	0.194041000000	-0.989357000000
C	-1.388294000000	1.660082000000	0.580680000000
H	-1.466439000000	0.989181000000	1.435460000000
H	-1.853052000000	2.609467000000	0.845790000000
N	0.022553000000	1.909121000000	0.307526000000
C	0.395446000000	3.255666000000	-0.105064000000
H	0.254502000000	3.955995000000	0.721443000000
H	1.428956000000	3.297705000000	-0.424648000000
H	-0.232709000000	3.569794000000	-0.940816000000
C	0.854124000000	0.852124000000	0.427694000000
O	0.477138000000	-0.268699000000	0.742798000000
C	2.355742000000	1.033863000000	0.156083000000
H	-5.438963000000	1.048898000000	-0.209834000000
F	-2.978543000000	-2.942169000000	0.432415000000
H	-1.645452000000	-1.466839000000	0.441499000000
F	-2.279723000000	-1.943416000000	-1.399120000000
F	2.577046000000	1.353361000000	-1.155444000000
H	2.867603000000	0.099885000000	0.382418000000
F	2.896082000000	2.031708000000	0.917422000000

conf1b

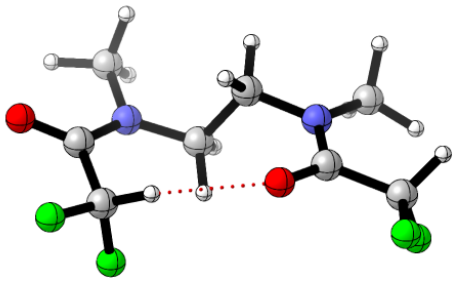

C	-4.475801000000	1.553384000000	-0.184639000000
H	-4.376938000000	2.132494000000	0.738287000000
H	-4.424873000000	2.234656000000	-1.035757000000
N	-3.428319000000	0.549798000000	-0.307194000000
C	-3.721129000000	-0.734707000000	0.051149000000
O	-4.807438000000	-1.089001000000	0.457517000000
C	-2.574999000000	-1.765824000000	-0.104284000000
C	-2.095044000000	1.019235000000	-0.638131000000
H	-2.177113000000	1.739814000000	-1.455928000000
H	-1.488514000000	0.198528000000	-1.005167000000
C	-1.389553000000	1.662033000000	0.567287000000
H	-1.478749000000	0.995687000000	1.424563000000
H	-1.845862000000	2.617422000000	0.826513000000
N	0.025872000000	1.895498000000	0.304947000000
C	0.414490000000	3.234047000000	-0.113958000000
H	0.248071000000	3.950496000000	0.694567000000
H	1.455942000000	3.270585000000	-0.412439000000
H	-0.179724000000	3.541874000000	-0.976912000000
C	0.848615000000	0.824735000000	0.433079000000
O	0.453616000000	-0.284181000000	0.742951000000
C	2.356005000000	1.066487000000	0.192974000000
H	-5.439569000000	1.053493000000	-0.171260000000
F	-2.986794000000	-2.956089000000	0.373274000000
H	-1.645593000000	-1.488272000000	0.397462000000
F	-2.302373000000	-1.924081000000	-1.445683000000
F	3.043476000000	-0.044418000000	0.506795000000
H	2.763187000000	1.898347000000	0.773827000000
F	2.567322000000	1.335291000000	-1.138331000000

conf1c

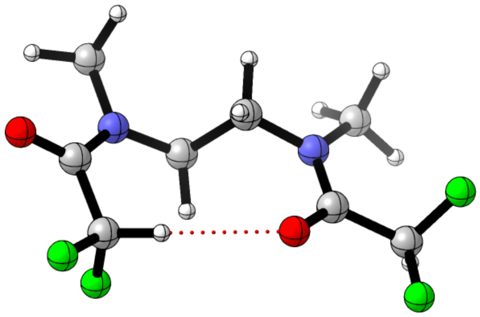

C	-4.481694000000	1.548993000000	-0.168388000000
H	-4.382215000000	2.113715000000	0.763287000000
H	-4.425036000000	2.242104000000	-1.009348000000
N	-3.438764000000	0.541870000000	-0.303914000000
C	-3.731504000000	-0.739589000000	0.064734000000
O	-4.814156000000	-1.087456000000	0.486074000000
C	-2.590187000000	-1.775390000000	-0.095194000000
C	-2.109296000000	1.004662000000	-0.659474000000
H	-2.201045000000	1.712837000000	-1.487355000000
H	-1.509395000000	0.176913000000	-1.021301000000
C	-1.384978000000	1.663924000000	0.524806000000
H	-1.463507000000	1.010926000000	1.393464000000
H	-1.834733000000	2.624026000000	0.777511000000
N	0.027841000000	1.887502000000	0.237340000000
C	0.429104000000	3.231344000000	-0.152878000000
H	0.419459000000	3.910181000000	0.703394000000
H	1.426872000000	3.240756000000	-0.578960000000
H	-0.262358000000	3.608340000000	-0.907889000000
C	0.856929000000	0.829898000000	0.423150000000
O	0.462308000000	-0.279393000000	0.731388000000
C	2.368255000000	1.081984000000	0.217034000000
H	-5.448413000000	1.054464000000	-0.164422000000
F	-3.002530000000	-2.962786000000	0.387875000000
H	-1.657044000000	-1.498622000000	0.400253000000
F	-2.325177000000	-1.939431000000	-1.438037000000
F	2.800947000000	2.039610000000	1.101302000000
H	2.616036000000	1.418085000000	-0.793837000000
F	3.050399000000	-0.048381000000	0.467849000000

conf23

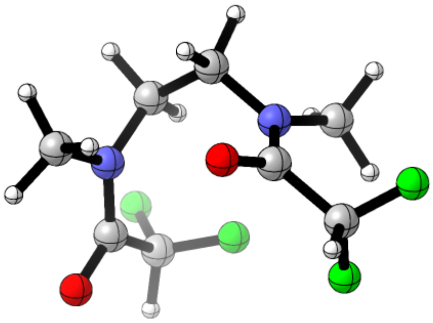

C	-3.809795000000	0.700783000000	1.115490000000
H	-3.159443000000	0.891100000000	1.969189000000
H	-4.514533000000	1.526194000000	0.998247000000
N	-3.028168000000	0.570193000000	-0.112211000000
C	-2.766089000000	-0.678796000000	-0.563786000000
O	-3.105497000000	-1.702634000000	0.006806000000
C	-2.027146000000	-0.868414000000	-1.901101000000
C	-2.584673000000	1.805951000000	-0.738147000000
H	-3.459447000000	2.445507000000	-0.894369000000
H	-2.169691000000	1.595697000000	-1.717484000000
C	-1.571614000000	2.593735000000	0.102044000000
H	-1.970705000000	2.795511000000	1.093276000000
H	-1.388905000000	3.551057000000	-0.392030000000
N	-0.293998000000	1.910399000000	0.256784000000
C	0.673693000000	2.093431000000	-0.819274000000
H	0.159106000000	2.021348000000	-1.775674000000
H	1.150292000000	3.074365000000	-0.744585000000
H	1.435456000000	1.324289000000	-0.799047000000
C	-0.060128000000	1.248378000000	1.411252000000
O	-0.854570000000	1.172928000000	2.337083000000
C	1.289808000000	0.536533000000	1.605674000000
H	-4.352959000000	-0.224358000000	1.282026000000
F	-2.682634000000	-0.258073000000	-2.938669000000
H	-1.964718000000	-1.935285000000	-2.106576000000
F	-0.758531000000	-0.354788000000	-1.869260000000
F	1.474310000000	-0.453263000000	0.682462000000
H	1.307859000000	0.095994000000	2.601165000000
F	2.337987000000	1.408514000000	1.490206000000

conf23b

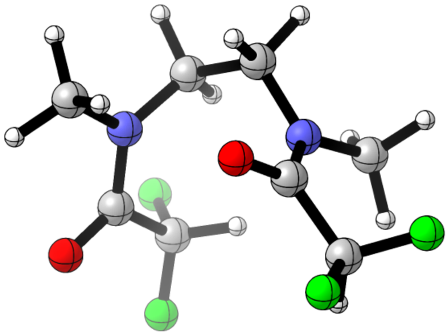

C	-3.906405000000	0.712031000000	1.095721000000
H	-3.285697000000	0.919642000000	1.968452000000
H	-4.629523000000	1.517492000000	0.958122000000
N	-3.088535000000	0.598327000000	-0.109232000000
C	-2.710656000000	-0.646613000000	-0.503903000000
O	-3.014109000000	-1.668249000000	0.075556000000
C	-1.848158000000	-0.737250000000	-1.786997000000
C	-2.647272000000	1.839831000000	-0.720343000000
H	-3.512986000000	2.500979000000	-0.820239000000
H	-2.294329000000	1.653409000000	-1.731103000000
C	-1.578804000000	2.577277000000	0.095867000000
H	-1.944730000000	2.782959000000	1.098772000000
H	-1.359620000000	3.531525000000	-0.389696000000
N	-0.336602000000	1.821403000000	0.218599000000
C	0.629242000000	1.979565000000	-0.860931000000
H	0.096274000000	2.010777000000	-1.811616000000
H	1.202121000000	2.903894000000	-0.753918000000
H	1.327686000000	1.148692000000	-0.899816000000
C	-0.086760000000	1.195180000000	1.403127000000
O	-0.871424000000	1.157769000000	2.327329000000
C	1.280323000000	0.482276000000	1.526415000000
H	-4.429130000000	-0.226694000000	1.250991000000
F	-1.466299000000	-2.013588000000	-1.981483000000
H	-0.950171000000	-0.117603000000	-1.761071000000
F	-2.592811000000	-0.351131000000	-2.877221000000
F	2.292468000000	1.404388000000	1.401437000000
H	1.433805000000	-0.293375000000	0.770808000000
F	1.384501000000	-0.081988000000	2.740575000000

conf23c

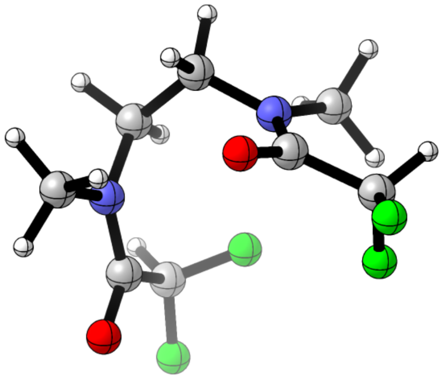

C	-3.795532000000	0.671193000000	1.122126000000
H	-3.134065000000	0.844815000000	1.970695000000
H	-4.491175000000	1.507007000000	1.023321000000
N	-3.033828000000	0.544418000000	-0.119109000000
C	-2.756053000000	-0.712154000000	-0.563669000000
O	-3.079713000000	-1.725032000000	0.019378000000
C	-2.054821000000	-0.830549000000	-1.939647000000
C	-2.598859000000	1.780676000000	-0.744353000000
H	-3.473599000000	2.423275000000	-0.893058000000
H	-2.183195000000	1.577017000000	-1.726980000000
C	-1.581643000000	2.577410000000	0.085956000000
H	-1.989703000000	2.793818000000	1.070452000000
H	-1.397571000000	3.527927000000	-0.421726000000
N	-0.308356000000	1.897079000000	0.269040000000
C	0.674901000000	2.034779000000	-0.798678000000
H	0.159168000000	2.049789000000	-1.756211000000
H	1.244184000000	2.964284000000	-0.696161000000
H	1.352467000000	1.187916000000	-0.824150000000
C	-0.071430000000	1.286701000000	1.459751000000
O	-0.866655000000	1.249265000000	2.377340000000
C	1.318838000000	0.626531000000	1.620335000000
H	-4.348331000000	-0.248957000000	1.284492000000
F	-0.774051000000	-0.329083000000	-1.876559000000
H	-2.581196000000	-0.299027000000	-2.737606000000
F	-1.963797000000	-2.125589000000	-2.286049000000
F	1.484551000000	0.238963000000	2.898505000000
H	2.149315000000	1.285074000000	1.352592000000
F	1.386285000000	-0.487488000000	0.824627000000

conf44

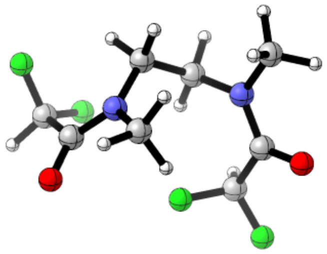

C	-2.619712000000	0.803627000000	1.610385000000
H	-3.427704000000	0.306442000000	2.142855000000
H	-1.676787000000	0.497209000000	2.064493000000
N	-2.676916000000	0.443184000000	0.195387000000
C	-2.931899000000	-0.856622000000	-0.085912000000
O	-3.123967000000	-1.709733000000	0.762969000000
C	-3.026288000000	-1.308840000000	-1.554131000000
C	-2.367851000000	1.457132000000	-0.802353000000
H	-2.826329000000	2.392991000000	-0.477569000000
H	-2.836604000000	1.196493000000	-1.746897000000
C	-0.871319000000	1.672948000000	-1.063717000000
H	-0.779066000000	2.339630000000	-1.927406000000
H	-0.426110000000	0.723855000000	-1.340142000000
N	-0.146031000000	2.267391000000	0.050011000000
C	-0.484795000000	3.639353000000	0.407666000000
H	-1.267729000000	3.681118000000	1.168947000000
H	0.395829000000	4.134910000000	0.807579000000
H	-0.830798000000	4.160949000000	-0.484332000000
C	0.772498000000	1.636948000000	0.830853000000
O	1.290107000000	2.152668000000	1.800029000000
C	1.190345000000	0.206916000000	0.410066000000
H	-2.730478000000	1.881760000000	1.701739000000
F	-4.060423000000	-0.687718000000	-2.202301000000
H	-3.192893000000	-2.384102000000	-1.575851000000
F	-1.884390000000	-1.028123000000	-2.261220000000
F	2.177213000000	-0.221484000000	1.216436000000
H	1.537582000000	0.140819000000	-0.624407000000
F	0.129936000000	-0.653267000000	0.556470000000

conf44b

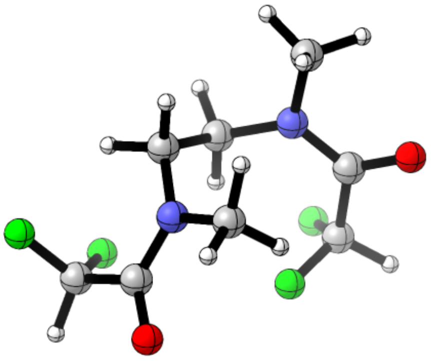

C	-2.614139000000	0.783812000000	1.596614000000
H	-3.423591000000	0.288468000000	2.128812000000
H	-1.671726000000	0.466356000000	2.044684000000
N	-2.680836000000	0.437286000000	0.179309000000
C	-2.956358000000	-0.855473000000	-0.113667000000
O	-3.147377000000	-1.715678000000	0.729048000000
C	-3.077866000000	-1.288010000000	-1.585999000000
C	-2.369690000000	1.460137000000	-0.810305000000
H	-2.826836000000	2.393972000000	-0.477385000000
H	-2.839880000000	1.208338000000	-1.756283000000
C	-0.872771000000	1.664348000000	-1.068459000000
H	-0.768325000000	2.327744000000	-1.932846000000
H	-0.422542000000	0.716502000000	-1.335940000000
N	-0.154601000000	2.262010000000	0.051053000000
C	-0.494971000000	3.634612000000	0.406280000000
H	-1.269981000000	3.675648000000	1.175617000000
H	0.387667000000	4.136121000000	0.794305000000
H	-0.852515000000	4.150215000000	-0.484396000000
C	0.781079000000	1.645942000000	0.811136000000
O	1.307104000000	2.162421000000	1.783981000000
C	1.245945000000	0.222900000000	0.451277000000
H	-2.715351000000	1.861924000000	1.698759000000
F	-4.118191000000	-0.648531000000	-2.207700000000
H	-3.257285000000	-2.361038000000	-1.618215000000
F	-1.946962000000	-1.008979000000	-2.306718000000
F	0.221456000000	-0.679206000000	0.529576000000
H	2.023155000000	-0.069376000000	1.155380000000
F	1.752970000000	0.158514000000	-0.816795000000

conf44c

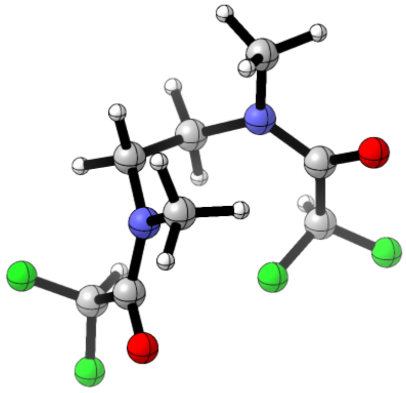

C	-2.827870000000	1.066051000000	1.586543000000
H	-3.465969000000	0.395471000000	2.154683000000
H	-1.859374000000	1.122969000000	2.087642000000
N	-2.708134000000	0.557499000000	0.222931000000
C	-2.890784000000	-0.780838000000	0.039694000000
O	-3.136368000000	-1.559151000000	0.934526000000
C	-2.778514000000	-1.300375000000	-1.413943000000
C	-2.388559000000	1.499659000000	-0.838322000000
H	-2.840087000000	2.456340000000	-0.571141000000
H	-2.862022000000	1.195180000000	-1.770376000000
C	-0.888542000000	1.705244000000	-1.107551000000
H	-0.793801000000	2.438792000000	-1.914992000000
H	-0.456353000000	0.776759000000	-1.468054000000
N	-0.137489000000	2.179282000000	0.042760000000
C	-0.453675000000	3.512792000000	0.541175000000
H	-1.264680000000	3.498349000000	1.272440000000
H	0.425016000000	3.929837000000	1.025787000000
H	-0.744672000000	4.141209000000	-0.300444000000
C	0.716460000000	1.425699000000	0.790968000000
O	1.168159000000	1.791990000000	1.855245000000
C	1.180407000000	0.079989000000	0.179329000000
H	-3.276057000000	2.059253000000	1.558934000000
F	-2.801520000000	-2.643666000000	-1.414448000000
H	-1.874190000000	-0.975205000000	-1.932704000000
F	-3.861674000000	-0.862027000000	-2.142918000000
F	2.176675000000	-0.426874000000	0.923467000000
H	1.536483000000	0.177447000000	-0.849952000000
F	0.149107000000	-0.829442000000	0.183604000000

conf68

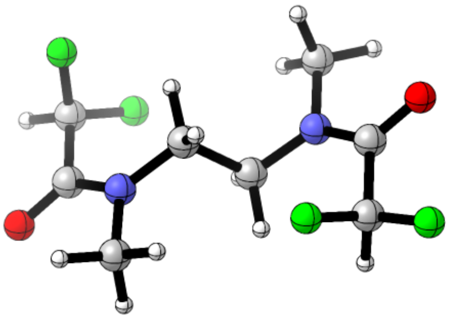

C	-2.640273000000	-0.977520000000	1.399167000000
H	-3.618178000000	-1.281635000000	1.769887000000
H	-2.068903000000	-1.882373000000	1.184679000000
N	-2.796366000000	-0.152322000000	0.201904000000
C	-3.538983000000	-0.678113000000	-0.798253000000
O	-4.071488000000	-1.774322000000	-0.738660000000
C	-3.759038000000	0.136433000000	-2.083663000000
C	-2.054497000000	1.098843000000	0.133161000000
H	-2.112375000000	1.586414000000	1.106882000000
H	-2.516468000000	1.765110000000	-0.588497000000
C	-0.580524000000	0.867034000000	-0.229192000000
H	-0.523647000000	0.416655000000	-1.221144000000
H	-0.144345000000	0.158361000000	0.469863000000
N	0.184152000000	2.103606000000	-0.238096000000
C	-0.048036000000	3.025611000000	-1.347724000000
H	-0.798156000000	3.778666000000	-1.095423000000
H	0.878067000000	3.541483000000	-1.589475000000
H	-0.391749000000	2.454910000000	-2.208484000000
C	0.960089000000	2.552741000000	0.784142000000
O	1.479544000000	3.649186000000	0.801966000000
C	1.213063000000	1.561673000000	1.945777000000
H	-2.124848000000	-0.394264000000	2.158259000000
F	-4.426983000000	1.300639000000	-1.828871000000
H	-4.343753000000	-0.460520000000	-2.781208000000
F	-2.571031000000	0.472845000000	-2.685213000000
F	2.070922000000	2.104228000000	2.825447000000
H	1.617375000000	0.600476000000	1.618134000000
F	0.032020000000	1.312782000000	2.614454000000

conf68b

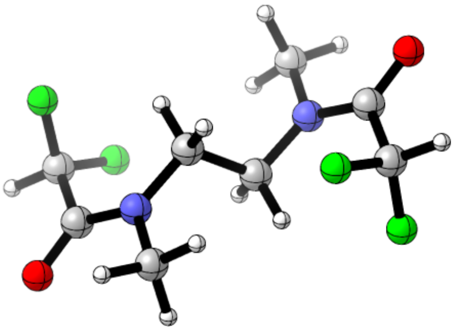

C	-2.606369000000	-1.047585000000	1.347777000000
H	-3.573411000000	-1.373892000000	1.727760000000
H	-2.031880000000	-1.937852000000	1.085914000000
N	-2.794898000000	-0.177817000000	0.187481000000
C	-3.559200000000	-0.665387000000	-0.814609000000
O	-4.096066000000	-1.760911000000	-0.783615000000
C	-3.793670000000	0.192308000000	-2.069683000000
C	-2.050751000000	1.073019000000	0.144497000000
H	-2.106373000000	1.539869000000	1.128734000000
H	-2.513483000000	1.755218000000	-0.561055000000
C	-0.579730000000	0.841637000000	-0.223786000000
H	-0.525611000000	0.391845000000	-1.215956000000
H	-0.127066000000	0.141947000000	0.470860000000
N	0.177700000000	2.084647000000	-0.243333000000
C	-0.031047000000	2.986254000000	-1.374192000000
H	-0.758623000000	3.765806000000	-1.136716000000
H	0.908397000000	3.470212000000	-1.630949000000
H	-0.393969000000	2.405381000000	-2.219548000000
C	0.955746000000	2.536040000000	0.766135000000
O	1.496844000000	3.629745000000	0.771507000000
C	1.199446000000	1.634413000000	1.989688000000
H	-2.077463000000	-0.490338000000	2.117099000000
F	-4.424613000000	1.367306000000	-1.769549000000
H	-4.411149000000	-0.370571000000	-2.767263000000
F	-2.612892000000	0.514482000000	-2.692168000000
F	0.023961000000	1.334971000000	2.633487000000
H	1.851301000000	2.160302000000	2.684748000000
F	1.783694000000	0.447174000000	1.647463000000

conf68c

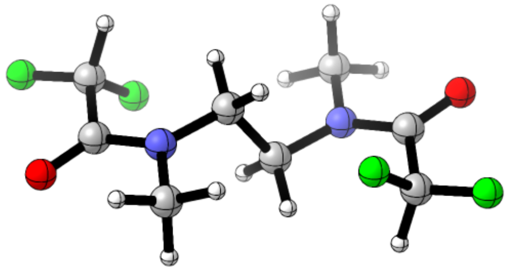

C	-2.591396000000	-1.051620000000	1.354554000000
H	-3.529456000000	-1.532601000000	1.622131000000
H	-1.862517000000	-1.836651000000	1.139685000000
N	-2.801663000000	-0.179101000000	0.201197000000
C	-3.544775000000	-0.682248000000	-0.820790000000
O	-4.046334000000	-1.786849000000	-0.803479000000
C	-3.784517000000	0.255237000000	-2.028638000000
C	-2.047765000000	1.063176000000	0.156748000000
H	-2.106139000000	1.540902000000	1.135523000000
H	-2.491136000000	1.747928000000	-0.561617000000
C	-0.571964000000	0.833004000000	-0.202803000000
H	-0.512583000000	0.355490000000	-1.181621000000
H	-0.129675000000	0.147991000000	0.516058000000
N	0.182174000000	2.075195000000	-0.246897000000
C	-0.026721000000	2.945960000000	-1.401758000000
H	-0.767085000000	3.722183000000	-1.194047000000
H	0.908407000000	3.437855000000	-1.659080000000
H	-0.369007000000	2.338867000000	-2.237528000000
C	0.924445000000	2.578824000000	0.775538000000
O	1.427181000000	3.682843000000	0.758210000000
C	1.160788000000	1.642448000000	1.984968000000
H	-2.233615000000	-0.448070000000	2.186336000000
F	-2.591363000000	0.497921000000	-2.677207000000
H	-4.213101000000	1.221607000000	-1.751056000000
F	-4.609647000000	-0.338065000000	-2.906126000000
F	1.983460000000	2.236844000000	2.864000000000
H	1.589955000000	0.675691000000	1.709700000000
F	-0.034239000000	1.400615000000	2.630527000000

conf68d

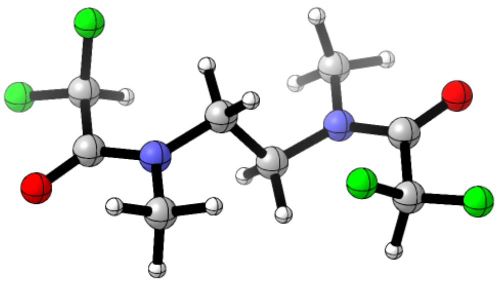

C	-2.595297000000	-1.001305000000	1.384423000000
H	-3.564769000000	-1.330304000000	1.755074000000
H	-2.001876000000	-1.892112000000	1.169202000000
N	-2.774622000000	-0.177969000000	0.189526000000
C	-3.559268000000	-0.695357000000	-0.790875000000
O	-4.086622000000	-1.785548000000	-0.716805000000
C	-3.764322000000	0.178949000000	-2.050993000000
C	-2.045013000000	1.076609000000	0.110383000000
H	-2.104745000000	1.577123000000	1.077543000000
H	-2.514548000000	1.739233000000	-0.610884000000
C	-0.566754000000	0.856187000000	-0.245862000000
H	-0.492726000000	0.399916000000	-1.236684000000
H	-0.131304000000	0.148253000000	0.454394000000
N	0.195750000000	2.094147000000	-0.254585000000
C	-0.016902000000	2.994525000000	-1.383148000000
H	-0.903504000000	3.618457000000	-1.245056000000
H	0.845212000000	3.647440000000	-1.484059000000
H	-0.137442000000	2.400421000000	-2.289300000000
C	0.935605000000	2.561909000000	0.790047000000
O	1.447544000000	3.660430000000	0.816402000000
C	1.154026000000	1.584626000000	1.970857000000
H	-2.093961000000	-0.407705000000	2.145223000000
F	-4.459834000000	-0.515280000000	-2.968236000000
H	-2.830500000000	0.512901000000	-2.512254000000
F	-4.494752000000	1.297487000000	-1.725550000000
F	1.966641000000	2.148000000000	2.878355000000
H	1.583851000000	0.625883000000	1.669184000000
F	-0.049944000000	1.327428000000	2.592564000000

conf76

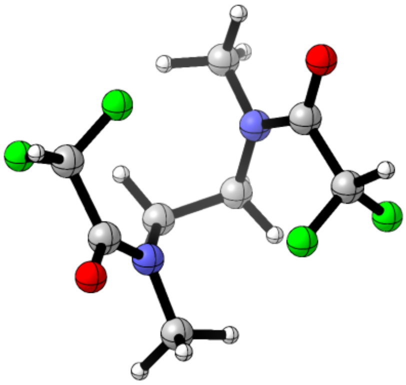

C	-1.912742000000	-0.815921000000	1.901241000000
H	-2.797053000000	-0.635613000000	2.502879000000
H	-1.702915000000	-1.888958000000	1.871353000000
N	-2.157182000000	-0.324545000000	0.550577000000
C	-3.417605000000	0.061215000000	0.234171000000
O	-4.343942000000	0.107966000000	1.025879000000
C	-3.755673000000	0.433294000000	-1.219997000000
C	-1.035062000000	-0.417422000000	-0.372143000000
H	-1.386005000000	-0.349350000000	-1.395611000000
H	-0.597196000000	-1.414346000000	-0.259156000000
C	0.081383000000	0.607738000000	-0.121407000000
H	0.990552000000	0.229929000000	-0.600352000000
H	0.298741000000	0.674025000000	0.938569000000
N	-0.182650000000	1.935244000000	-0.660365000000
C	-0.062634000000	2.078718000000	-2.107261000000
H	-0.599269000000	2.965733000000	-2.427145000000
H	0.988410000000	2.177120000000	-2.394984000000
H	-0.486409000000	1.206676000000	-2.599753000000
C	-0.329052000000	3.072311000000	0.061299000000
O	-0.428158000000	4.184290000000	-0.430446000000
C	-0.366768000000	3.006484000000	1.597810000000
H	-1.066724000000	-0.300367000000	2.352120000000
F	-3.003082000000	1.472409000000	-1.688318000000
H	-4.806247000000	0.713373000000	-1.266972000000
F	-3.547369000000	-0.631205000000	-2.059512000000
F	-1.351739000000	2.183337000000	2.062426000000
H	-0.538567000000	4.011381000000	1.978928000000
F	0.820614000000	2.543174000000	2.106050000000

conf164

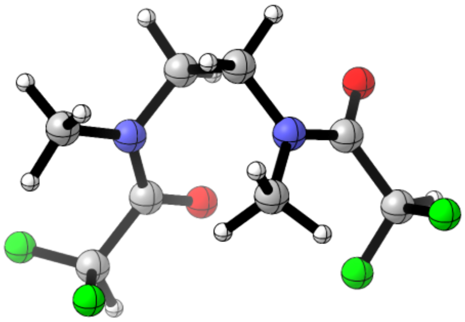

C	-2.185298000000	-1.465539000000	-0.774908000000
H	-2.299186000000	-1.478087000000	-1.850975000000
H	-1.167252000000	-1.783589000000	-0.539555000000
N	-2.431332000000	-0.141334000000	-0.223822000000
C	-2.866397000000	0.935260000000	-0.920155000000
O	-3.053711000000	2.039835000000	-0.437875000000
C	-3.141509000000	0.789615000000	-2.426676000000
C	-2.193984000000	0.014743000000	1.209803000000
H	-2.565369000000	-0.878844000000	1.718041000000
H	-2.760009000000	0.874275000000	1.557851000000
C	-0.716997000000	0.212381000000	1.551687000000
H	-0.119853000000	-0.635231000000	1.214774000000
H	-0.624019000000	0.285427000000	2.635558000000
N	-0.154220000000	1.415882000000	0.946325000000
C	0.633678000000	1.268263000000	-0.271028000000
H	0.001562000000	1.093891000000	-1.143556000000
H	1.237295000000	2.150364000000	-0.449502000000
H	1.307336000000	0.418575000000	-0.150926000000
C	-0.491173000000	2.593615000000	1.520727000000
O	-1.151079000000	2.680160000000	2.543623000000
C	-0.017820000000	3.907663000000	0.877985000000
H	-2.881407000000	-2.187408000000	-0.340459000000
F	-1.999330000000	0.460259000000	-3.110351000000
H	-3.511327000000	1.740638000000	-2.805511000000
F	-4.068371000000	-0.181150000000	-2.686659000000
F	1.346016000000	4.027406000000	0.970090000000
H	-0.477626000000	4.739295000000	1.409327000000
F	-0.342320000000	3.990973000000	-0.443296000000

conf164b

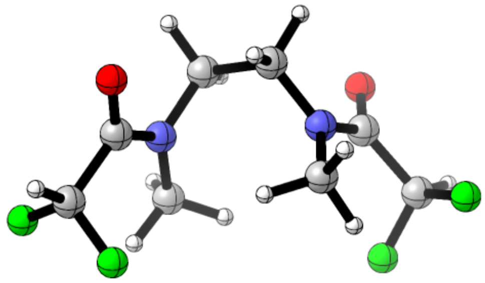

C	-3.037246000000	1.014328000000	-0.893880000000
H	-3.041370000000	0.896566000000	-1.970298000000
H	-4.066656000000	1.140283000000	-0.550293000000
N	-2.420520000000	-0.133422000000	-0.237037000000
C	-2.095319000000	-1.302325000000	-0.826588000000
O	-1.573864000000	-2.238076000000	-0.235860000000
C	-2.367687000000	-1.497874000000	-2.326956000000
C	-2.181703000000	-0.025146000000	1.203947000000
H	-2.495709000000	-0.952468000000	1.683802000000
H	-2.793652000000	0.790893000000	1.582337000000
C	-0.715831000000	0.225109000000	1.552546000000
H	-0.112374000000	-0.617317000000	1.221970000000
H	-0.631001000000	0.317701000000	2.635486000000
N	-0.174166000000	1.435402000000	0.930926000000
C	0.679735000000	1.253801000000	-0.237586000000
H	0.124534000000	0.755320000000	-1.033017000000
H	1.041745000000	2.202708000000	-0.611763000000
H	1.538431000000	0.634208000000	0.028359000000
C	-0.510770000000	2.615605000000	1.490369000000
O	-1.245035000000	2.720117000000	2.463379000000
C	0.024633000000	3.923456000000	0.884908000000
H	-2.476128000000	1.916214000000	-0.650540000000
F	-3.677978000000	-1.279996000000	-2.647126000000
H	-2.104746000000	-2.518867000000	-2.598360000000
F	-1.610969000000	-0.636779000000	-3.079584000000
F	1.390454000000	3.964174000000	0.874901000000
H	-0.347134000000	4.758024000000	1.477063000000
F	-0.400817000000	4.079264000000	-0.409218000000

conf164c

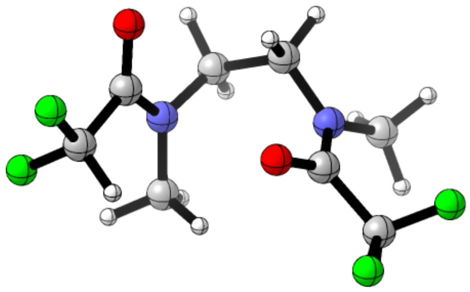

C	-2.434031000000	-1.506001000000	-0.709421000000
H	-2.337027000000	-1.573823000000	-1.788298000000
H	-1.589193000000	-2.034594000000	-0.266021000000
N	-2.421573000000	-0.124969000000	-0.248632000000
C	-2.736580000000	0.954358000000	-1.006107000000
O	-2.688793000000	2.103508000000	-0.611150000000
C	-3.148251000000	0.670635000000	-2.470473000000
C	-2.170144000000	0.074453000000	1.181006000000
H	-2.579208000000	-0.786877000000	1.713493000000
H	-2.705047000000	0.962929000000	1.506917000000
C	-0.688607000000	0.226945000000	1.525162000000
H	-0.108411000000	-0.606587000000	1.127296000000
H	-0.589066000000	0.224597000000	2.610352000000
N	-0.113409000000	1.464184000000	1.008271000000
C	0.562183000000	1.445625000000	-0.283023000000
H	-0.121939000000	1.650358000000	-1.110797000000
H	1.370115000000	2.171662000000	-0.305930000000
H	0.999185000000	0.459429000000	-0.429871000000
C	-0.479860000000	2.605828000000	1.653435000000
O	-1.113030000000	2.613448000000	2.689374000000
C	-0.077955000000	3.932207000000	0.973081000000
H	-3.356540000000	-2.013365000000	-0.416318000000
F	-4.262091000000	-0.130336000000	-2.494804000000
H	-2.364456000000	0.176204000000	-3.051843000000
F	-3.456621000000	1.828681000000	-3.081847000000
F	-0.602054000000	4.967802000000	1.652312000000
H	-0.414798000000	3.987026000000	-0.064692000000
F	1.292768000000	4.073175000000	0.992039000000

conf164d

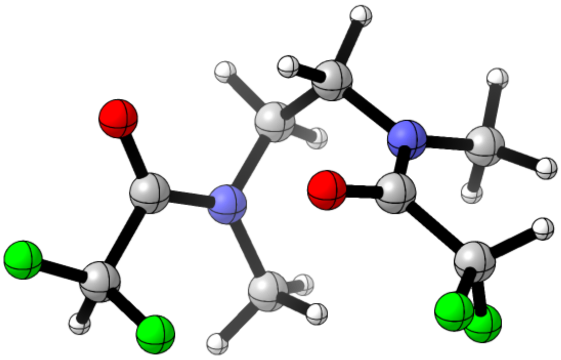

C	-2.186210000000	-1.449121000000	-0.757706000000
H	-2.733903000000	-1.648228000000	-1.674265000000
H	-1.120358000000	-1.549793000000	-0.977534000000
N	-2.516695000000	-0.143150000000	-0.207219000000
C	-2.902715000000	0.935432000000	-0.943947000000
O	-3.128565000000	2.034198000000	-0.485151000000
C	-3.045580000000	0.701998000000	-2.466599000000
C	-2.261659000000	0.054305000000	1.217675000000
H	-2.648462000000	-0.812445000000	1.759664000000
H	-2.806023000000	0.937313000000	1.539984000000
C	-0.778052000000	0.226814000000	1.546330000000
H	-0.199406000000	-0.629743000000	1.198272000000
H	-0.677766000000	0.290023000000	2.629839000000
N	-0.192277000000	1.427659000000	0.956964000000
C	0.512474000000	1.309122000000	-0.313240000000
H	-0.158381000000	1.364166000000	-1.171061000000
H	1.260776000000	2.090169000000	-0.421906000000
H	1.031940000000	0.351181000000	-0.332557000000
C	-0.475802000000	2.603670000000	1.579378000000
O	-1.108262000000	2.675405000000	2.613315000000
C	0.098591000000	3.882722000000	0.928701000000
H	-2.465622000000	-2.212877000000	-0.031137000000
F	-3.430051000000	1.842727000000	-3.062166000000
H	-3.757495000000	-0.083647000000	-2.732401000000
F	-1.816973000000	0.350728000000	-2.985091000000
F	-0.411658000000	4.042965000000	-0.331067000000
H	1.190225000000	3.866633000000	0.851612000000
F	-0.253319000000	4.955286000000	1.662890000000

conf164e

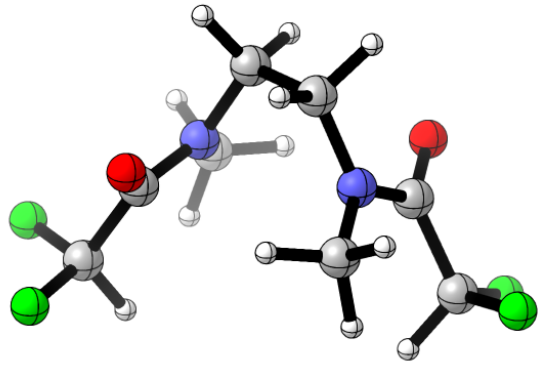

C	-3.569020000000	0.925712000000	-0.449525000000
H	-3.508826000000	1.207801000000	-1.496566000000
H	-4.555841000000	0.491122000000	-0.272995000000
N	-2.508310000000	-0.011416000000	-0.089383000000
C	-1.878857000000	-0.843606000000	-0.946344000000
O	-1.031046000000	-1.649137000000	-0.601101000000
C	-2.254514000000	-0.714338000000	-2.439584000000
C	-2.205942000000	-0.154326000000	1.332857000000
H	-2.389223000000	-1.185859000000	1.641397000000
H	-2.889138000000	0.498471000000	1.865664000000
C	-0.753661000000	0.192475000000	1.722198000000
H	-0.131279000000	-0.689980000000	1.617673000000
H	-0.759581000000	0.498860000000	2.770653000000
N	-0.134521000000	1.240326000000	0.913526000000
C	1.086254000000	0.882501000000	0.196086000000
H	0.944580000000	-0.091938000000	-0.267949000000
H	1.313468000000	1.603804000000	-0.582756000000
H	1.941726000000	0.839117000000	0.874576000000
C	-0.662753000000	2.480446000000	0.986570000000
O	-1.660520000000	2.750023000000	1.633881000000
C	0.045230000000	3.585708000000	0.171514000000
H	-3.455847000000	1.824755000000	0.152966000000
F	-1.480027000000	-1.536517000000	-3.173356000000
H	-2.137610000000	0.299647000000	-2.831241000000
F	-3.562568000000	-1.088916000000	-2.619663000000
F	-0.657097000000	4.731405000000	0.259174000000
H	0.163212000000	3.341215000000	-0.887502000000
F	1.293454000000	3.810727000000	0.695159000000

conf_Xray

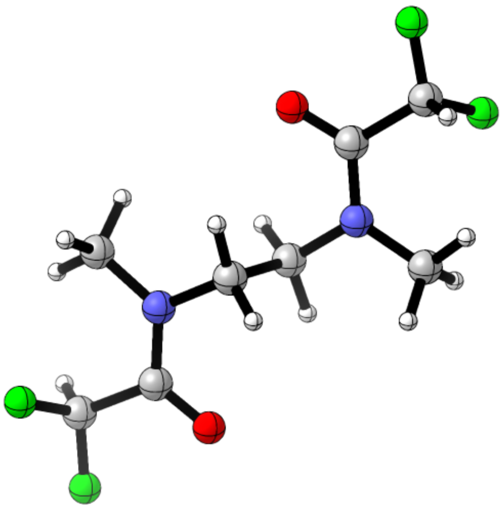

O	2.801146000000	6.572135000000	7.106259000000
C	0.013768000000	4.599526000000	5.898354000000
N	0.961876000000	5.682004000000	6.126782000000
F	3.453224000000	4.379253000000	8.369560000000
F	2.490925000000	3.288518000000	6.722297000000
C	2.052569000000	5.628621000000	6.930016000000
C	2.319766000000	4.286950000000	7.649949000000
H	1.510887000000	3.987035000000	8.321996000000
C	0.760099000000	6.915294000000	5.374410000000
H	0.169809000000	6.688893000000	4.487804000000
H	1.733610000000	7.292899000000	5.063693000000
O	-1.799130000000	8.627748000000	4.330744000000
C	1.057142000000	10.221708000000	5.891053000000
N	0.022419000000	9.263920000000	5.521200000000
F	-2.025149000000	10.909281000000	3.069365000000
F	-1.181348000000	11.834598000000	4.877049000000
C	-0.968563000000	9.468603000000	4.620173000000
C	-0.995800000000	10.853448000000	3.933994000000
H	-0.077812000000	11.082625000000	3.386095000000
C	0.046769000000	7.980063000000	6.212648000000
H	0.577183000000	8.112668000000	7.154144000000
H	-0.977260000000	7.672033000000	6.419812000000
H	0.221437000000	4.082236000000	4.958658000000
H	-0.993640000000	5.013839000000	5.854924000000
H	0.042968000000	3.869538000000	6.701460000000
H	1.197982000000	10.975647000000	5.122582000000
H	1.999226000000	9.689017000000	6.022032000000
H	0.808265000000	10.729233000000	6.826054000000

conf250

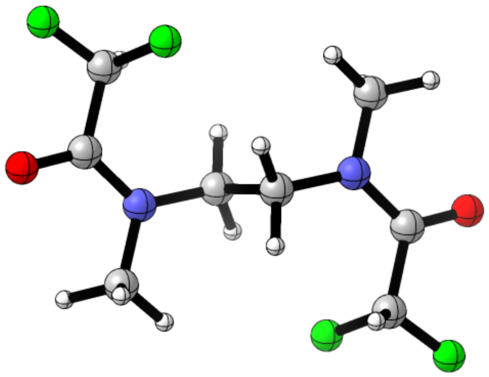

C	-3.906571000000	0.067844000000	-0.086711000000
H	-4.583023000000	-0.731054000000	-0.384204000000
H	-3.653938000000	-0.035351000000	0.966328000000
N	-2.676239000000	-0.007789000000	-0.872535000000
C	-2.821957000000	-0.128558000000	-2.218772000000
O	-3.900533000000	-0.209504000000	-2.768541000000
C	-1.522225000000	-0.204646000000	-3.054598000000
C	-1.420536000000	0.219536000000	-0.176393000000
H	-0.587438000000	-0.147358000000	-0.770699000000
H	-1.429470000000	-0.351607000000	0.752746000000
C	-1.198230000000	1.704562000000	0.147350000000
H	-2.031883000000	2.074314000000	0.739235000000
H	-1.187688000000	2.273395000000	-0.783274000000
N	0.057287000000	1.932897000000	0.843457000000
C	1.285121000000	1.853292000000	0.054970000000
H	2.001296000000	2.584810000000	0.421837000000
H	1.747228000000	0.865886000000	0.129385000000
H	1.044890000000	2.059263000000	-0.986228000000
C	0.201914000000	2.043696000000	2.191270000000
O	1.278787000000	2.113580000000	2.745831000000
C	-1.099293000000	2.125798000000	3.024628000000
H	-4.422872000000	1.017521000000	-0.243473000000
F	-1.827895000000	-0.391595000000	-4.348732000000
H	-0.846139000000	-1.004116000000	-2.740266000000
F	-0.835202000000	0.987779000000	-2.955196000000
F	-1.790669000000	0.935401000000	2.926325000000
H	-1.772569000000	2.926746000000	2.708327000000
F	-0.795212000000	2.313231000000	4.319110000000

conf250b

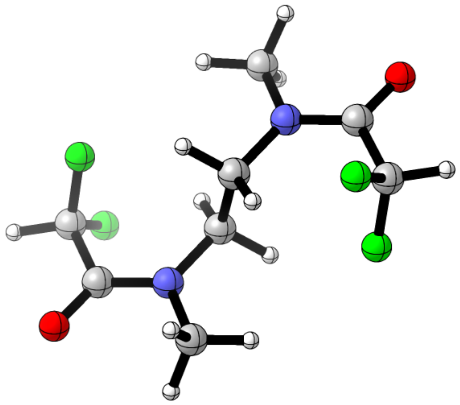

C	-3.914134000000	0.110038000000	-0.116858000000
H	-4.591084000000	-0.691637000000	-0.405787000000
H	-3.673036000000	0.027548000000	0.940452000000
N	-2.675113000000	0.013903000000	-0.886994000000
C	-2.801275000000	-0.136825000000	-2.224431000000
O	-3.874950000000	-0.224578000000	-2.797596000000
C	-1.537676000000	-0.208756000000	-3.099198000000
C	-1.421074000000	0.223706000000	-0.178633000000
H	-0.594573000000	-0.161877000000	-0.766058000000
H	-1.451273000000	-0.347334000000	0.750608000000
C	-1.195381000000	1.704910000000	0.147513000000
H	-2.023749000000	2.090357000000	0.732419000000
H	-1.162818000000	2.275641000000	-0.781811000000
N	0.057007000000	1.916115000000	0.858473000000
C	1.295622000000	1.817572000000	0.088536000000
H	1.997278000000	2.578032000000	0.424351000000
H	1.769779000000	0.841937000000	0.217125000000
H	1.063266000000	1.965801000000	-0.963780000000
C	0.180207000000	2.059601000000	2.197314000000
O	1.251786000000	2.139495000000	2.775451000000
C	-1.085921000000	2.131860000000	3.068758000000
H	-4.422208000000	1.059750000000	-0.296982000000
F	-0.790546000000	0.938587000000	-2.996198000000
H	-1.840473000000	-0.334036000000	-4.137146000000
F	-0.725527000000	-1.250641000000	-2.748195000000
F	-1.899885000000	3.171411000000	2.715728000000
H	-0.785413000000	2.258253000000	4.107223000000
F	-1.830319000000	0.982648000000	2.964756000000

conf250c

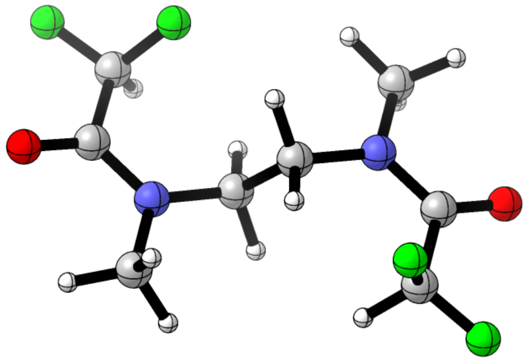

C	-3.899758000000	0.072703000000	-0.064025000000
H	-4.694580000000	-0.470890000000	-0.566142000000
H	-3.710962000000	-0.370020000000	0.914294000000
N	-2.682839000000	-0.014656000000	-0.864598000000
C	-2.826332000000	-0.136706000000	-2.214358000000
O	-3.898035000000	-0.212723000000	-2.775705000000
C	-1.520354000000	-0.223381000000	-3.040639000000
C	-1.425285000000	0.221063000000	-0.174588000000
H	-0.592158000000	-0.156736000000	-0.761807000000
H	-1.434457000000	-0.351365000000	0.757138000000
C	-1.195732000000	1.706647000000	0.141015000000
H	-2.020581000000	2.100889000000	0.726945000000
H	-1.181195000000	2.269388000000	-0.792978000000
N	0.061703000000	1.924748000000	0.836465000000
C	1.293492000000	1.868933000000	0.052029000000
H	1.964967000000	2.665573000000	0.366014000000
H	1.815240000000	0.919250000000	0.191151000000
H	1.043208000000	1.991998000000	-0.999510000000
C	0.198971000000	2.064699000000	2.181193000000
O	1.273459000000	2.145739000000	2.738696000000
C	-1.108527000000	2.112082000000	3.009161000000
H	-4.225648000000	1.107389000000	0.069868000000
F	-1.819176000000	-0.424355000000	-4.333961000000
H	-0.847268000000	-1.020409000000	-2.714090000000
F	-0.833595000000	0.969085000000	-2.949622000000
F	-0.809935000000	2.148877000000	4.319368000000
H	-1.767412000000	1.257120000000	2.832650000000
F	-1.812369000000	3.252798000000	2.704201000000

Additional Computed Structures with Benzyl Substituents

Method: B3LYP-D3(BJ)/def2-TZVP

Conf23b_Ph

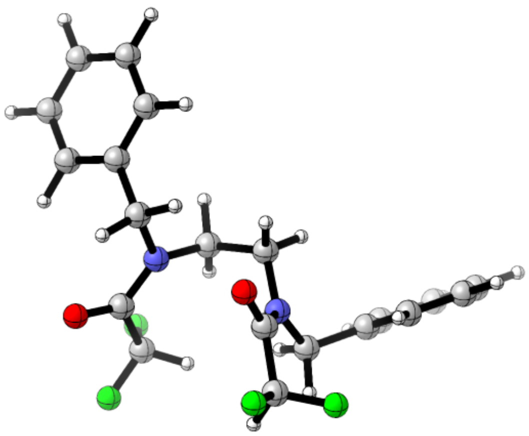

C	-4.088238000000	0.005116000000	1.048349000000
H	-4.192256000000	-1.049317000000	1.291063000000
H	-3.577500000000	0.485967000000	1.881735000000
N	-3.219508000000	0.077057000000	-0.134991000000
C	-2.987801000000	-1.067325000000	-0.828789000000
O	-3.435002000000	-2.156702000000	-0.532139000000
C	-2.074045000000	-0.948262000000	-2.073154000000
C	-2.772435000000	1.406073000000	-0.526370000000
H	-3.636050000000	2.076410000000	-0.503907000000
H	-2.431548000000	1.396840000000	-1.557172000000
C	-1.694772000000	1.986268000000	0.392064000000
H	-2.047856000000	2.029884000000	1.418491000000
H	-1.468830000000	3.003360000000	0.069559000000
N	-0.457234000000	1.210345000000	0.375108000000
C	0.502183000000	1.544898000000	-0.675640000000
H	0.021120000000	1.418243000000	-1.649579000000
C	1.022335000000	2.963152000000	-0.577724000000
H	1.334302000000	0.846476000000	-0.643749000000
C	-0.250889000000	0.324027000000	1.389258000000
O	-1.026425000000	0.155092000000	2.307158000000
C	1.037624000000	-0.532683000000	1.317960000000
C	-5.433259000000	0.639085000000	0.796891000000
F	-1.768503000000	-2.177866000000	-2.527282000000
H	-1.139976000000	-0.415869000000	-1.886189000000
F	-2.742754000000	-0.285729000000	-3.076498000000
F	2.140854000000	0.265991000000	1.513165000000

H	1.162748000000	-1.052421000000	0.364515000000
F	1.015782000000	-1.450756000000	2.297824000000
C	1.532979000000	3.457619000000	0.621693000000
C	0.996920000000	3.791012000000	-1.696330000000
C	2.009991000000	4.759102000000	0.696041000000
C	1.984824000000	5.580593000000	-0.427190000000
C	1.479400000000	5.093547000000	-1.625402000000
H	1.452109000000	5.726766000000	-2.502855000000
H	0.593823000000	3.416314000000	-2.630327000000
H	1.556056000000	2.823690000000	1.498296000000
H	2.403244000000	5.134067000000	1.632189000000
H	2.354563000000	6.595960000000	-0.366097000000
C	-5.831045000000	1.774705000000	1.496932000000
C	-6.291486000000	0.102361000000	-0.164909000000
C	-7.064643000000	2.368402000000	1.245739000000
C	-7.520676000000	0.693700000000	-0.419508000000
C	-7.910287000000	1.829955000000	0.285003000000
H	-8.869886000000	2.289698000000	0.085877000000
H	-7.361537000000	3.250717000000	1.798438000000
H	-5.990643000000	-0.784620000000	-0.708916000000
H	-8.178998000000	0.266208000000	-1.165181000000
H	-5.172797000000	2.196024000000	2.248137000000

Conf164d_Ph

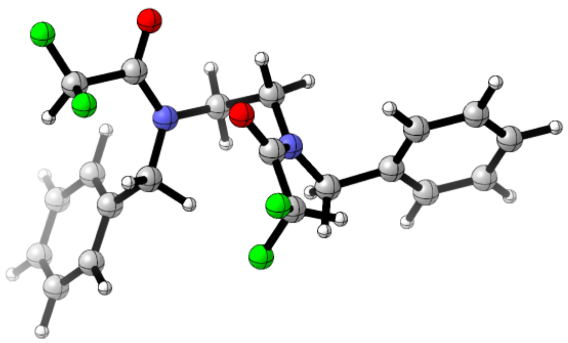

C	-2.142813000000	-1.489043000000	-1.162016000000
H	-1.953186000000	-1.313201000000	-2.219102000000
H	-1.199346000000	-1.870139000000	-0.762774000000
N	-2.437480000000	-0.229386000000	-0.496998000000
C	-2.957794000000	0.870401000000	-1.105727000000
O	-3.250370000000	1.896663000000	-0.531345000000

C	-3.150041000000	0.769129000000	-2.637562000000
C	-2.208935000000	-0.186869000000	0.947439000000
H	-2.478823000000	-1.159820000000	1.363002000000
H	-2.860646000000	0.568065000000	1.379006000000
C	-0.757832000000	0.136626000000	1.298325000000
H	-0.072628000000	-0.562701000000	0.816245000000
H	-0.637014000000	0.040723000000	2.377053000000
N	-0.365106000000	1.485211000000	0.906900000000
C	0.312281000000	1.679405000000	-0.366657000000
H	-0.048971000000	0.911764000000	-1.051288000000
H	-0.007856000000	2.622519000000	-0.806950000000
C	1.825911000000	1.619093000000	-0.302418000000
C	-0.675194000000	2.492175000000	1.766128000000
O	-1.273520000000	2.321220000000	2.808241000000
C	-0.164491000000	3.899551000000	1.374831000000
C	-3.213075000000	-2.550497000000	-0.992567000000
F	-3.817333000000	1.848827000000	-3.077234000000
H	-3.681285000000	-0.126031000000	-2.968737000000
F	-1.905793000000	0.769327000000	-3.236279000000
F	-0.928539000000	4.408696000000	0.358079000000
H	0.878550000000	3.902339000000	1.048648000000
F	-0.280550000000	4.723097000000	2.435522000000
C	2.554154000000	1.815738000000	-1.476755000000
C	2.513559000000	1.380804000000	0.882681000000
C	3.940677000000	1.777161000000	-1.464637000000
C	3.905217000000	1.339868000000	0.896724000000
C	4.622461000000	1.538136000000	-0.274595000000
H	2.028345000000	2.005973000000	-2.405819000000
H	4.491239000000	1.937083000000	-2.382957000000
H	4.425466000000	1.155605000000	1.828037000000
H	5.704291000000	1.510363000000	-0.263167000000
H	1.963547000000	1.231145000000	1.802437000000
C	-2.874945000000	-3.890197000000	-1.181632000000
C	-4.530677000000	-2.224731000000	-0.685785000000
C	-5.496553000000	-3.220379000000	-0.578321000000
C	-3.837472000000	-4.884949000000	-1.077645000000
C	-5.154003000000	-4.551538000000	-0.776018000000
H	-3.559487000000	-5.920703000000	-1.225201000000
H	-5.905088000000	-5.325891000000	-0.689624000000
H	-4.806703000000	-1.192322000000	-0.514600000000
H	-6.516872000000	-2.952178000000	-0.336218000000
H	-1.849009000000	-4.156605000000	-1.410303000000

Conf_crystal (DFT-optimised)

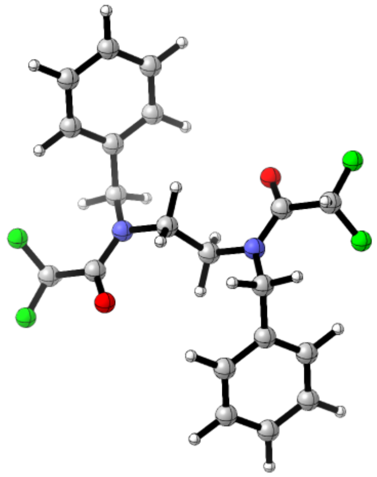

O	2.858364000000	6.610537000000	7.356635000000
C	0.106306000000	4.732154000000	5.899488000000
H	-0.865061000000	5.150009000000	6.177159000000
H	0.284366000000	3.871792000000	6.536409000000
N	1.139438000000	5.745997000000	6.155140000000
F	3.244021000000	4.469173000000	8.780204000000
F	2.570688000000	3.336725000000	7.021640000000
C	0.065905000000	4.296965000000	4.454437000000
C	1.028931000000	3.422084000000	3.952706000000
H	1.800943000000	3.041630000000	4.609648000000
C	1.002784000000	3.042108000000	2.617714000000
H	1.751540000000	2.358508000000	2.238147000000
C	0.016454000000	3.536699000000	1.769432000000
H	-0.001953000000	3.240299000000	0.728374000000
C	-0.942892000000	4.412141000000	2.262195000000
H	-1.709295000000	4.806840000000	1.607683000000
C	-0.918728000000	4.789269000000	3.600635000000
H	-1.657619000000	5.488131000000	3.975335000000
C	2.110623000000	5.684571000000	7.099291000000
C	2.247409000000	4.358505000000	7.882880000000
H	1.337562000000	4.072582000000	8.416808000000
C	0.997768000000	7.006269000000	5.438642000000
H	0.579808000000	6.800630000000	4.455880000000
H	1.988177000000	7.438644000000	5.316425000000
O	-1.771553000000	8.368720000000	4.283221000000
C	0.980920000000	10.246868000000	5.739920000000
H	1.952233000000	9.828961000000	5.462142000000
H	0.802837000000	11.107239000000	5.103017000000

N	-0.052294000000	9.233078000000	5.484367000000
F	-2.157429000000	10.510198000000	2.859890000000
F	-1.483408000000	11.642450000000	4.618323000000
C	1.021508000000	10.682064000000	7.184965000000
C	0.058583000000	11.556996000000	7.686800000000
H	-0.713488000000	11.937481000000	7.029944000000
C	0.084911000000	11.936993000000	9.021783000000
H	-0.663767000000	12.620634000000	9.401430000000
C	1.071327000000	11.442377000000	9.869951000000
H	1.089879000000	11.738798000000	10.911000000000
C	2.030580000000	10.566892000000	9.377082000000
H	2.797057000000	10.172184000000	10.031501000000
C	2.006232000000	10.189741000000	8.038652000000
H	2.745065000000	9.490862000000	7.663867000000
C	-1.023567000000	9.294557000000	4.540312000000
C	-1.160509000000	10.620706000000	3.756894000000
H	-0.250811000000	10.906585000000	3.222688000000
C	0.089364000000	7.972795000000	6.200848000000
H	0.507330000000	8.178424000000	7.183609000000
H	-0.901047000000	7.540426000000	6.323071000000

Compound 5

Method: B3LYP-D3(BJ)/def2-TZVP

5_SM

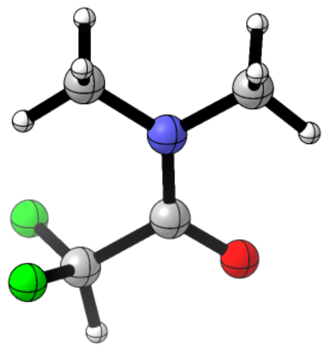

C	-1.578128000000	1.331456000000	-0.751848000000
N	-2.632789000000	0.390953000000	-0.408025000000
C	-3.968227000000	0.959878000000	-0.322503000000

H	-4.240127000000	1.407555000000	-1.282131000000
H	-4.708741000000	0.212503000000	-0.069018000000
H	-3.988290000000	1.743007000000	0.440148000000
C	-2.305682000000	-0.903439000000	-0.193080000000
O	-1.174772000000	-1.356766000000	-0.276096000000
C	-3.413063000000	-1.900912000000	0.191523000000
F	-4.034460000000	-1.541588000000	1.357052000000
H	-2.959995000000	-2.881601000000	0.325111000000
F	-4.377813000000	-1.992925000000	-0.775712000000
H	-1.775588000000	1.775843000000	-1.730890000000
H	-0.628630000000	0.806474000000	-0.775463000000
H	-1.535600000000	2.132707000000	-0.009290000000

5_TS1

Imaginary frequency: -129.89 cm^{-1}

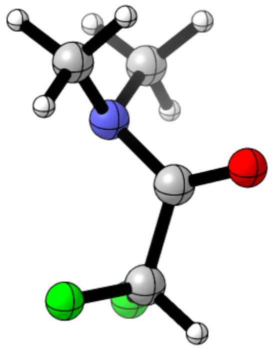

C	-1.829438000000	0.954123000000	-1.212362000000
N	-3.000398000000	0.354959000000	-0.563473000000
C	-3.843427000000	1.352036000000	0.104744000000
H	-4.202537000000	2.054438000000	-0.647504000000
H	-4.707611000000	0.860502000000	0.551183000000
H	-3.305900000000	1.907259000000	0.884459000000
C	-2.625106000000	-0.692298000000	0.326518000000
O	-1.950502000000	-0.572824000000	1.312879000000
C	-3.162251000000	-2.075235000000	-0.067379000000
F	-4.523927000000	-2.037144000000	-0.102298000000
H	-2.845051000000	-2.848267000000	0.633737000000
F	-2.715426000000	-2.393674000000	-1.314199000000
H	-2.180769000000	1.657186000000	-1.967703000000
H	-1.251457000000	0.177662000000	-1.713214000000
H	-1.178104000000	1.484421000000	-0.505611000000

5_TS2

Imaginary frequency: -130.59 cm^{-1}

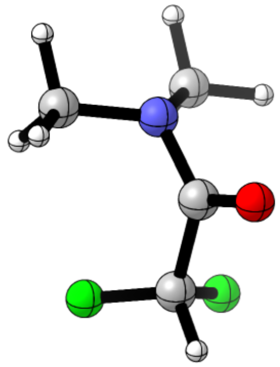

C	-3.067736000000	0.627316000000	-1.809176000000
N	-3.520283000000	0.494080000000	-0.425464000000
C	-2.888622000000	1.435580000000	0.497166000000
H	-3.136907000000	2.447962000000	0.173279000000
H	-3.288340000000	1.294065000000	1.500641000000
H	-1.795241000000	1.340644000000	0.534838000000
C	-3.656017000000	-0.828063000000	0.068547000000

O	-4.709799000000	-1.303726000000	0.388914000000
C	-2.399915000000	-1.709763000000	0.275486000000
F	-1.521678000000	-1.592112000000	-0.759741000000
H	-2.665948000000	-2.759627000000	0.400610000000
F	-1.759710000000	-1.280479000000	1.405861000000
H	-3.410832000000	1.593014000000	-2.186051000000
H	-3.522101000000	-0.149695000000	-2.423510000000
H	-1.978775000000	0.573951000000	-1.921626000000

5_SM_F_syn

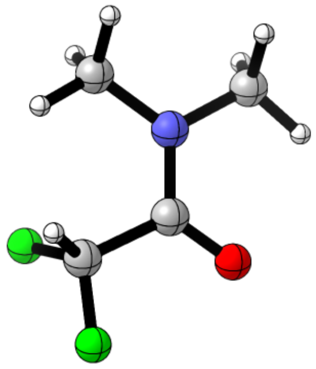

C	-1.588925000000	1.315279000000	-0.837522000000
N	-2.597537000000	0.396280000000	-0.332864000000
C	-3.834300000000	1.015441000000	0.112561000000
H	-4.397887000000	1.418777000000	-0.734091000000
H	-4.474669000000	0.314428000000	0.636332000000
H	-3.597612000000	1.837004000000	0.792007000000
C	-2.380149000000	-0.940418000000	-0.454776000000
O	-1.362896000000	-1.432186000000	-0.898594000000
C	-3.515767000000	-1.865422000000	0.046176000000
F	-4.639239000000	-1.682876000000	-0.725891000000
H	-3.792591000000	-1.690937000000	1.089408000000
F	-3.137197000000	-3.150183000000	-0.079334000000
H	-1.964700000000	1.852244000000	-1.713454000000
H	-0.704561000000	0.750649000000	-1.114631000000
H	-1.333877000000	2.045063000000	-0.065549000000

5_TS_F_syn

Imaginary frequency: -118.89 cm^{-1}

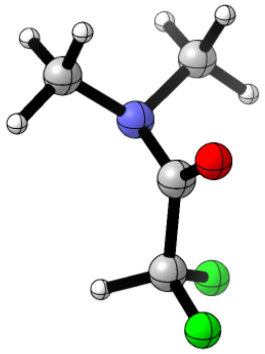

C	-1.851202000000	0.956900000000	-1.233386000000
N	-2.998837000000	0.340355000000	-0.558196000000
C	-3.835311000000	1.329276000000	0.130508000000
H	-4.228713000000	2.024118000000	-0.611458000000
H	-4.679494000000	0.831258000000	0.610001000000
H	-3.282560000000	1.892943000000	0.892864000000
C	-2.585051000000	-0.694113000000	0.340170000000
O	-1.889544000000	-0.552359000000	1.303235000000
C	-3.157479000000	-2.059592000000	-0.068166000000
F	-2.620510000000	-2.402588000000	-1.279980000000
H	-4.245196000000	-2.025900000000	-0.173349000000
F	-2.824972000000	-3.016476000000	0.825499000000
H	-2.228081000000	1.651163000000	-1.984597000000
H	-1.270273000000	0.186833000000	-1.740252000000
H	-1.196259000000	1.500121000000	-0.540711000000

11.References

(1) Rosenau, C. P.; Jelier, B. J.; Gossert, A. D.; Togni, A. Exposing the Origins of Irreproducibility in Fluorine NMR Spectroscopy. *Angewandte Chemie International Edition* **2018**, *57* (30), 9528–9533. https://doi.org/10.1002/anie.201802620.

(2) Combettes, L. E.; Clausen-Thue, P.; King, M. A.; Odell, B.; Thompson, A. L.; Gouverneur, V.; Claridge, T. D. W. Conformational Analysis of Fluorinated Pyrrolidines Using 19F-1H Scalar Couplings and Heteronuclear NOEs. *Chemistry - A European Journal* **2012**, *18* (41), 13133–13141. https://doi.org/10.1002/chem.201201577.

(3) Dewis, L.; Crouch, R.; Russell, D.; Butts, C. Improving the Accuracy of 1 H- 19 F Internuclear Distance Measurement Using 2D 1 H- 19 F HOESY. *Magnetic Resonance in Chemistry* **2019**, *57* (12), 1143–1149. https://doi.org/10.1002/mrc.4904.

(4) Smith, A. J. R.; York, R.; Uhrín, D.; Bell, N. G. A. 19 F-Centred NMR Analysis of Mono-Fluorinated Compounds. *RSC Advances* **2022**, *12* (16), 10062–10070. https://doi.org/10.1039/D1RA08046F.

(5) Contreras-García, J.; Johnson, E. R.; Keinan, S.; Chaudret, R.; Piquemal, J. P.; Beratan, D. N.; Yang, W. NCIPLOT: A Program for Plotting Noncovalent Interaction Regions. *Journal of Chemical Theory and Computation* **2011**, *7* (3), 625–632. https://doi.org/10.1021/ct100641a.

(6) Laplaza, R.; Peccati, F.; A. Boto, R.; Quan, C.; Carbone, A.; Piquemal, J. P.; Maday, Y.; Contreras-García, J. NCIPLOT and the Analysis of Noncovalent Interactions Using the Reduced Density Gradient. *Wiley Interdisciplinary Reviews: Computational Molecular Science* **2020**, No. March, 1–18. https://doi.org/10.1002/wcms.1497.

(7) Neese, F.; Wennmohs, F.; Becker, U.; Riplinger, C. The ORCA Quantum Chemistry Program Package. *The Journal of chemical physics* **2020**, *152* (22), 224108. https://doi.org/10.1063/5.0004608.

(8) Becke, A. D. Density-functional Thermochemistry. III. The Role of Exact Exchange. *The Journal of Chemical Physics* **1993**, *98* (7), 5648–5652. https://doi.org/10.1063/1.464913.

(9) Lee, C.; Yang, W.; Parr, R. G. Development of the Colle-Salvetti Correlation-Energy Formula into a Functional of the Electron Density. *Physical Review B* **1988**, *37* (2), 785–789. https://doi.org/10.1103/PhysRevB.37.785.

(10) Grimme, S. Density Functional Theory with London Dispersion Corrections. *Wiley Interdisciplinary Reviews: Computational Molecular Science* **2011**, *1* (2), 211–228. https://doi.org/10.1002/wcms.30.

(11) Weigend, F.; Ahlrichs, R. Balanced Basis Sets of Split Valence, Triple Zeta Valence and Quadruple Zeta Valence Quality for H to Rn: Design and Assessment of Accuracy. *Physical Chemistry Chemical Physics* **2005**, *7* (18), 3297–3305. https://doi.org/10.1039/b508541a.

(12) Grimme, S. Supramolecular Binding Thermodynamics by Dispersion-Corrected Density Functional Theory. *Chemistry - A European Journal* **2012**, *18* (32), 9955–9964.

https://doi.org/10.1002/chem.201200497.

(13) Grimme, S. Exploration of Chemical Compound, Conformer, and Reaction Space with Meta-Dynamics Simulations Based on Tight-Binding Quantum Chemical Calculations. *Journal of Chemical Theory and Computation* **2019**, *15* (5), 2847–2862. https://doi.org/10.1021/acs.jctc.9b00143.

(14) Marenich, A. V.; Cramer, C. J.; Truhlar, D. G. Universal Solvation Model Based on Solute Electron Density and on a Continuum Model of the Solvent Defined by the Bulk Dielectric Constant and Atomic Surface Tensions. *Journal of Physical Chemistry B* **2009**, *113* (18), 6378–6396. https://doi.org/10.1021/jp810292n.

(15) Liakos, D. G.; Sparta, M.; Kesharwani, M. K.; Martin, J. M. L.; Neese, F. Exploring the Accuracy Limits of Local Pair Natural Orbital Coupled-Cluster Theory. *Journal of Chemical Theory and Computation* **2015**, *11* (4), 1525–1539. https://doi.org/10.1021/ct501129s.

(16) Guo, Y.; Riplinger, C.; Becker, U.; Liakos, D. G.; Minenkov, Y.; Cavallo, L.; Neese, F. Communication: An Improved Linear Scaling Perturbative Triples Correction for the Domain Based Local Pair-Natural Orbital Based Singles and Doubles Coupled Cluster Method [DLPNO-CCSD(T)]. *Journal of Chemical Physics* **2018**, *148* (1). https://doi.org/10.1063/1.5011798.

(17) Kozuch, S.; Martin, J. M. L. DSD-PBEP86: In Search of the Best Double-Hybrid DFT with Spin-Component Scaled MP2 and Dispersion Corrections. *Physical Chemistry Chemical Physics* **2011**, *13* (45), 20104–20107. https://doi.org/10.1039/c1cp22592h.

(18) Stoychev, G. L.; Auer, A. A.; Neese, F. Efficient and Accurate Prediction of Nuclear Magnetic Resonance Shielding Tensors with Double-Hybrid Density Functional Theory. *Journal of Chemical Theory and Computation* **2018**, *14* (9), 4756–4771. https://doi.org/10.1021/acs.jctc.8b00624.

(19) Jensen, F. Segmented Contracted Basis Sets Optimized for Nuclear Magnetic Shielding. *Journal of Chemical Theory and Computation* **2015**, *11* (1), 132–138. https://doi.org/10.1021/ct5009526.
